# From the Design to the *In Vivo* Evaluation of Benzohomoadamantane-Derived Soluble Epoxide Hydrolase Inhibitors for the Treatment of Acute Pancreatitis

**DOI:** 10.1021/acs.jmedchem.0c01601

**Published:** 2021-05-04

**Authors:** Sandra Codony, Carla Calvó-Tusell, Elena Valverde, Sílvia Osuna, Christophe Morisseau, M. Isabel Loza, José Brea, Concepción Pérez, María Isabel Rodríguez-Franco, Javier Pizarro-Delgado, Rubén Corpas, Christian Griñán-Ferré, Mercè Pallàs, Coral Sanfeliu, Manuel Vázquez-Carrera, Bruce D. Hammock, Ferran Feixas, Santiago Vázquez

**Affiliations:** † Laboratori de Química Farmacèutica (Unitat Associada al CSIC), Departament de Farmacologia, Toxicologia i Química Terapèutica, Facultat de Farmàcia i Ciències de l’Alimentació, and Institute of Biomedicine (IBUB), Universitat de Barcelona, Av. Joan XXIII, 27-31, Barcelona 08028, Spain; ‡ CompBioLab Group, Departament de Química and Institut de Química Computacional i Catàlisi (IQCC), Universitat de Girona, C/ Maria Aurèlia Capmany 69, Girona 17003, Spain; § Institució Catalana de Recerca i Estudis Avançats (ICREA), Barcelona 08010, Spain; ∥ Department of Entomology and Nematology and Comprehensive Cancer Center, University of California Davis, One Shields Avenue, Davis 95616, California, United States; ⊥ Drug Screening Platform/Biofarma Research Group, CIMUS Research Center. Departamento de Farmacoloxía, Farmacia e Tecnoloxía Farmacéutica, University of Santiago de Compostela (USC), Santiago de Compostela 15782, Spain; # Institute of Medicinal Chemistry, Spanish National Research Council (CSIC), C/Juan de la Cierva 3, Madrid 28006, Spain; ¶ Pharmacology Section. Department of Pharmacology, Toxicology and Medicinal Chemistry, Faculty of Pharmacy and Food Sciences, and Institute of Biomedicine of the University of Barcelona (IBUB), University of Barcelona, Av. Joan XXIII, 27-31, Barcelona 08028, Spain; ∇ Spanish Biomedical Research Center in Diabetes and Associated Metabolic Diseases (CIBERDEM)-Instituto de Salud Carlos III, Madrid 28029, Spain; ○ Pediatric Research Institute-Hospital Sant Joan de Déu, Esplugues de Llobregat 08950, Spain; ⧫ Institute of Biomedical Research of Barcelona (IIBB), CSIC and IDIBAPS, Barcelona 08036, Spain; †† CIBER Epidemiology and Public Health (CIBERESP)-Instituto de Salud Carlos III, Madrid 28029, Spain; ‡‡ Pharmacology Section. Department of Pharmacology, Toxicology and Medicinal Chemistry, Faculty of Pharmacy and Food Sciences, and Institut de Neurociències, University of Barcelona, Av. Joan XXIII, 27-31, Barcelona 08028, Spain

## Abstract

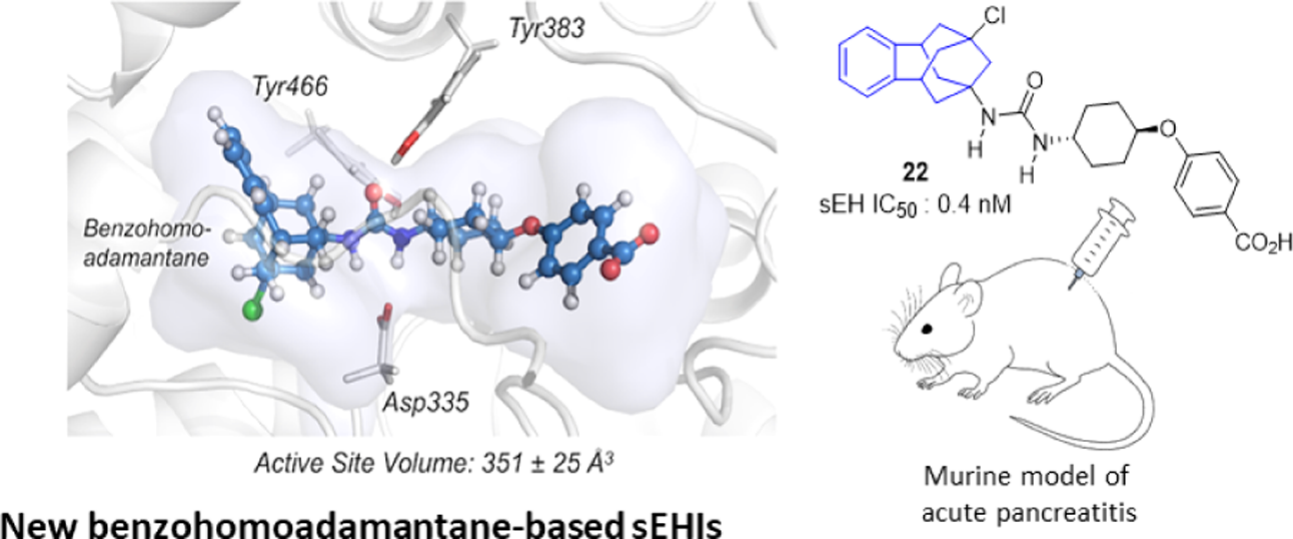

The pharmacological inhibition of soluble epoxide hydrolase (sEH) is efficient for the treatment of inflammatory and pain-related diseases. Numerous potent sEH inhibitors (sEHIs) present adamantyl or phenyl moieties, such as the clinical candidates AR9281 or EC5026. Herein, in a new series of sEHIs, these hydrophobic moieties have been merged in a benzohomoadamantane scaffold. Most of the new sEHIs have excellent inhibitory activities against sEH. Molecular dynamics simulations suggested that the addition of an aromatic ring into the adamantane scaffold produced conformational rearrangements in the enzyme to stabilize the aromatic ring of the benzohomoadamantane core. A screening cascade permitted us to select a candidate for an *in vivo* efficacy study in a murine model of cerulein-induced acute pancreatitis. The administration of **22** improved the health status of the animals and reduced pancreatic damage, demonstrating that the benzohomoadamantane unit is a promising scaffold for the design of novel sEHIs.

## 1 Introduction

In mammals, arachidonic acid, a polyunsaturated fatty acid, is metabolized by cyclooxygenases (COXs), lipoxygenases (LOXs), and cytochrome P450s (CYPs). The COX and LOX pathways lead mainly to the production of pro-inflammatory lipid mediators, such as prostaglandins and leukotrienes, and are pharmaceutically targeted.^1^ In contrast, the CYP pathway produces both pro- and anti-inflammatory lipid mediators, such as the pro-inflammatory 20-hydroxyeicosatetraenoic acid or the potent anti-inflammatory epoxyeicosatrienoic acids (EETs).^2^ However, the EETs are rapidly metabolized by the soluble epoxide hydrolase (sEH, *EPHX*2, EC 3.3.2.3) into the corresponding dihydroxyeicosatrienoic acids, which are less biologically active.^3,4^ The pharmacological inhibition of sEH *in vivo* stabilizes the concentration of EETs, reducing inflammatory and pain states, suggesting sEH as a pharmacological target for the treatment of inflammatory diseases.^5,6^

**Figure 1:**
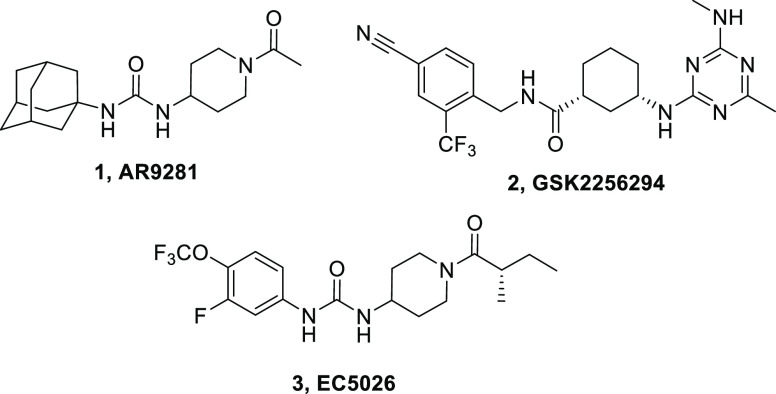
Structure of the three sEHIs that have entered human clinical trials.

X-ray crystallographic studies revealed that sEH has an L-shaped active pocket with the catalytic residues situated at the corner. Although each side of the pocket (10 and 15 Å long) accepts a variety of functional groups, the entire pocket is essentially hydrophobic.^7^ Indeed, a number of very potent sEH inhibitors (sEHIs) feature lipophilic moieties such as adamantyl or phenyl groups (Figure 1), limiting their usefulness.^5,6^

Nevertheless, several compounds have been tested in human. AR9281, which was developed by Arête Therapeutics for the treatment of hypertension in diabetic patients, failed in a phase II clinical trial largely due to its poor pharmacokinetic properties, likely related with its adamantyl unit.^8^ GSK2256294, developed for chronic obstructive pulmonary disease by GlaxoSmithKline, has entered clinical trials for obese smokers and other indications such as subarachnoidal hemorrhage or diabetic patients with insulin resistance.^9^ More recently, taking the AR9281 failure into account, EicOsis has recently replaced the adamantyl moiety of AR9281 by an aromatic ring for its drug candidate EC5026, which has recently completed human phase 1a clinical trials for the treatment of neuropathic pain.^10^

**Figure 2:**
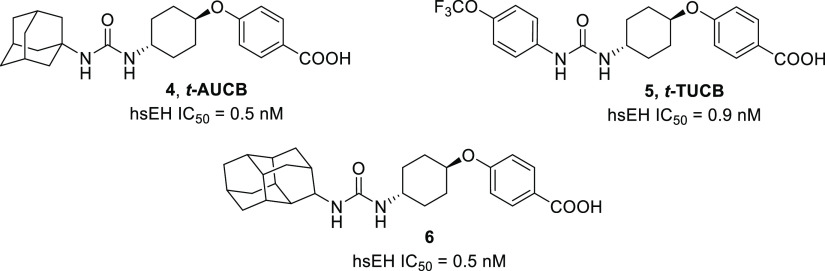
Structure and IC_50_ in hsEH of compounds **4–6**.

Using urea-based sEHIs with lipophilic units of very different sizes, we recently found that the pocket of the sEH can accommodate polycycles of a quite diverse volume and that the replacement of the adamantane moiety by larger polycyclic rings, such as the diamantane moiety, may be better than the replacement by smaller ones. Indeed, urea **6**, a diamantane analogue of the well-known sEHI **4**, *t*-AUCB, and **5**, *t*-TUCB, showed to be a subnanomolar inhibitor of the human sEH (hsEH) (Figure 2).^11^ Therefore, herein we are testing new sEH that merge the adamantyl and phenyl groups in a unique polycyclic scaffold.

## 2 Results and Discussion

### 2.1 Design and Synthesis of New sEHIs

**Figure 3:**
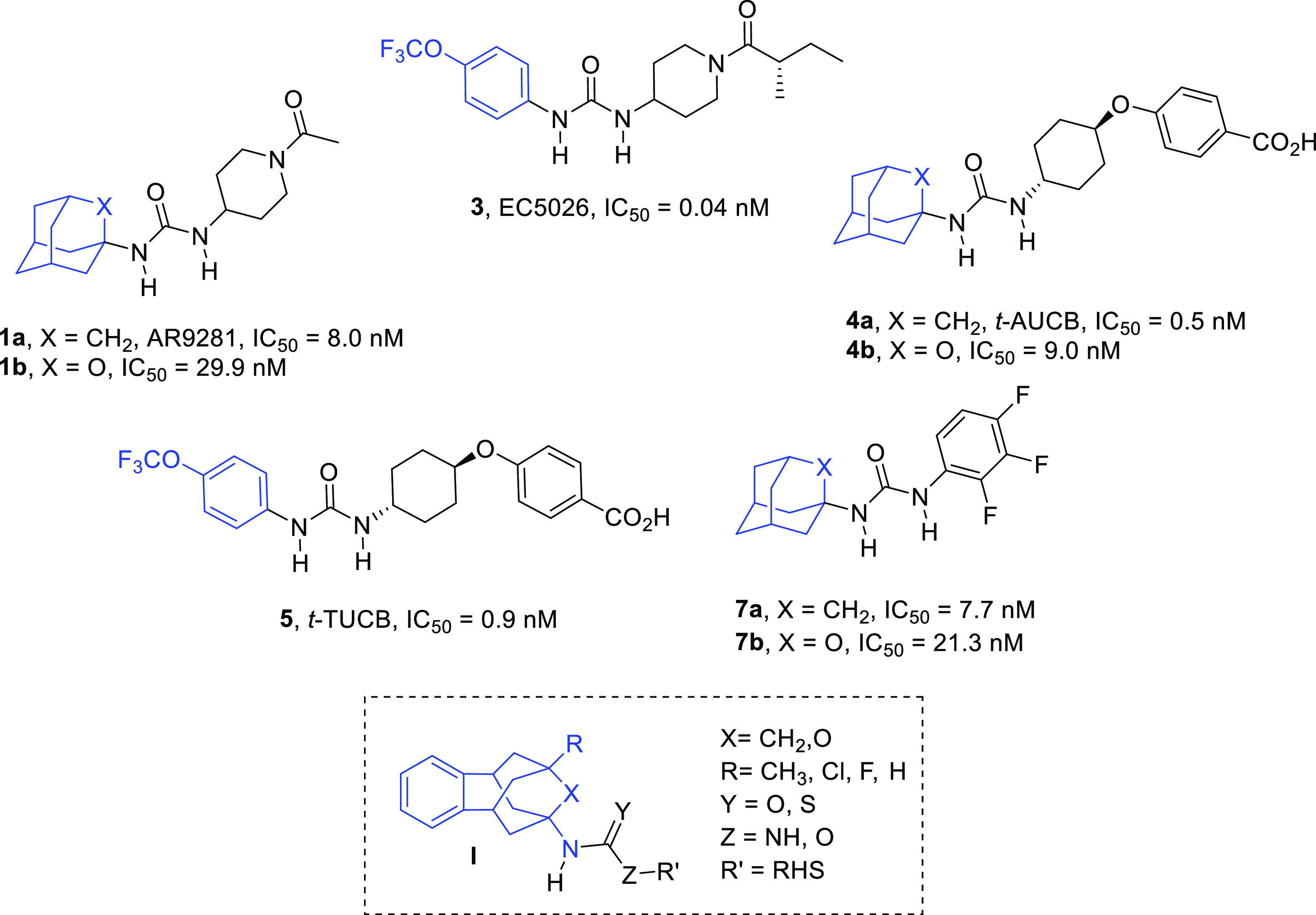
Known adamantyl- and phenyl-based sEHIs **1a**, **b**, **3**, **4a**, **b**, **5**, and **7a**, **b**, and general structure, **I**, of the new sEHI reported in this work. (see below). For simplicity, only IC_50_ values obtained for the hsEH are reported.

Taking into account that both adamantane and aromatic ring moieties fit very well in the hydrophobic pocket of the sEH and that the replacement of adamantane by larger polycyclic rings seems to be a promising strategy to obtain more potent sEHIs, a novel series of compounds bearing the very versatile benzohomoadamantane scaffold as the hydrophobic moiety were designed and synthesized. This polycyclic, readily accessible,^12−15^ system features a homoadamantane unit fused with an aromatic ring and permits several chemical derivatizations in its structure (Figure 3). Potent sEHIs and optimum drug-like properties could be achieved by modifying the substituents in the benzohomoadamantane unit and/or the right-hand side (RHS) of the molecule (Figure 3).

**Scheme 1:**
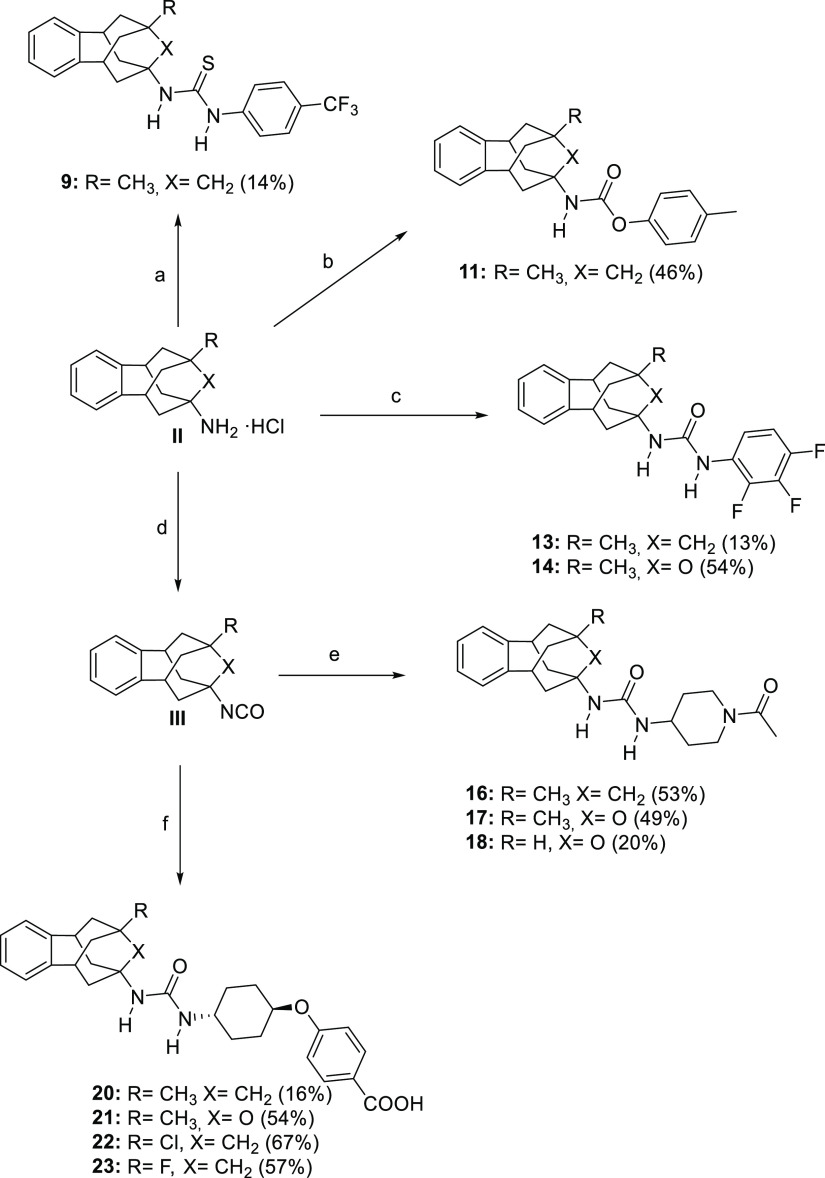
Synthesis of New Compounds **9**, **11**, **13**, **14**, **16–18**, and **20–23** Reagents and conditions: (a) 4-(trifluoromethyl)phenyl isothiocyanate (**8**), Et_3_N, DCM, overnight (b) *p*-tolyl chloroformate (**10**), Et_3_N, DCM, overnight; (c) 2,3,4-trifluorophenyl isocyanate (**12**), DCM, overnight; (d) Triphosgene, sat. NaHCO_3_, DCM, 30 min; (e) 1-acetyl-4-aminopiperidine (**15**), DCM, overnight; (f) 4-((*trans*-4-aminocyclohexyl)oxy)benzoic acid hydrochloride^20^ (**19**), Et_3_N, DMF, overnight. See the Experimental Section and the Supporting Information for further details.

Thioureas, carbamates and, particularly ureas are good pharmacophores for an sEHI.^6^ For this reason, thiourea **9**, carbamate **11**, and urea **13** were first synthesized in order to explore their relative potency as an sEHI and to select the more suitable pharmacophore for the novel polycyclic scaffold (Scheme 1). The three compounds were easily synthesized in low to moderate yields from known 9-methyl-5,6,8,9,10,11-hexahydro-7*H*-5,9:7,11-dimethanobenzo[9]annulen-7-amine^13^ (**II**, R = CH_3_, X = CH_2_) and 4-(trifluoromethyl)phenyl isothiocyanate, **8**, *p*-tolyl chloroformate, **10**, and 2,3,4-trifluorophenyl isocyanate, **12**, respectively (Scheme 1).

The inhibitory activities of the three compounds in hsEH were evaluated using a previously reported sensitive fluorescent-based assay (Table 1).^16^ While carbamate **11** was a very weak inhibitor (IC_50_ = 12.7 μM) and thiourea **9** displayed only a moderate inhibition (IC_50_ = 138 nM), urea **13** revealed as a very potent hsEHI (IC_50_ = 1 nM) (Table 1). The superior potency of the urea is in agreement with previous results in other series of sEHIs.^17,18^ For this reason, no further carbamates and thioureas derivatives were envisaged and the urea group was chosen as the main pharmacophore for the synthesis of further inhibitors.

**Table 1:** Inhibition of hsEH and Microsomal Stability Values of the New Benzohomoadamantane-Based sEHI

Cpd	hsEH^a^IC_50_ (nM)	microsomal stability^b^ (h/m/r)
**9**	138	ND^c^
**11**	>10,000	ND
**7**	7.7	79/77/81
**13**	1.0	7/0.2/ND
**14**	20	77/23/33
1, **AR9281**	8.0	72/100/87
**16**	3.1	1/0.5/ND
**17**	941	ND
**18**	>10,000	ND
4, t-AUCB	0.5	94/92/46
**20**	0.9	70/10/2
**21**	28	90/83/ND

a Reported IC_50_ values are the average of three replicates. The fluorescent assay as performed here has a standard error between 10 and 20% suggesting that differences of twofold or greater are significant. Because of the limitations of the assay, it is difficult to distinguish among potencies <0.5 nM.^16^

b Percentage of the remaining compound after 60 min of incubation with human, mice, and rat microsomes obtained from Tebu–Xenotech in the presence of NADP at 37 °C.

c ND: not determined.

Having found that this novel scaffold may successfully replace the adamantane and/or the phenyl group found in known sEHIs, a series of benzohomoadamantane derivatives related with the potent sEHI AR9281, *t*-AUCB, *t*-TUCB, EC5026, and **7** were synthesized in order to explore their potency and DMPK properties. Of note, a very recent work has described that the replacement of a methylene unit of the adamantane moiety by an oxygen atom led to more soluble compounds while only slightly reducing the inhibitory activity against the sEH (*e.g.*, **1b**, **4b**, and **7b** in Figure 3).^19^ In this sense, an oxygen atom was introduced in the benzohomoadamantane scaffold in order to explore whether a similar trend was also followed within this new family of sEHIs (Figure 3 and Scheme 1).

The synthesis of the new sEHI started from the suitably substituted benzohomoadamantane amines of general structure **II**.^12−15^ Thus, the synthesis of urea **14** involved the reaction of 5-methyl-1,5,6,7-tetrahydro-1,5:3,7-dimethanobenzo[*e*]oxonin-3(2*H*)-amine^12^ (**II**, R = CH_3_, X = O) with 2,3,4-trifluorophenylisocyanate, **12**, in dichloromethane (Scheme 1). For the obtention of the piperidine derivatives, we first prepared the isocyanate of the corresponding polycyclic amine **II** (**II**, R = CH_3_, X = CH_2_;^13^**II**, R = CH_3_, X = O;^12^**II**, R = H, X = O^12^) by reaction with triphosgene and saturated the aqueous solution of NaHCO_3_. Once the desired isocyanate of general structure **III** (**III**, R = CH_3_, X = CH_2_; **III**, R = CH_3_, X = O; **III**, R = H, X = O) was obtained, it was reacted with 1-acetyl-4-aminopiperidine **15** in dichloromethane to obtain ureas **16–18** in moderate overall yields (Scheme 1). Finally, the *t*-AUCB analogues **20–23** were obtained in low to moderate yields by the reaction, in the presence of triethylamine, of the corresponding isocyanate, obtained from **II** (**II**, R = CH_3_, X = CH_2_;^13^**II**, R = CH_3_, X = O;^12^**II**, R = Cl, X = CH_2_;^15^**II**, R = F, X = CH_2_^15^) and triphosgene, in DMF with 4-((*trans*-4-aminocyclohexyl)oxy)benzoic acid, **19**, prepared as previously reported^20^ (Scheme 1).

### 2.2 sEH Inhibition and DMPK Assays

The potency of the new compounds as inhibitors of the hsEH was tested using a previously reported sensitive fluorescent-based assay.^16^ Gratifyingly, the potency of the new benzohomoadamantane ureas was in the same range as that of diphosgene their corresponding adamantane-based analogues (compare **13***vs***7**, **16***vs* AR9281, and **20***vs t*-AUCB) (Table 1). The comparison of the compounds presenting a methylene unit in the polycyclic scaffold with their analogues featuring an oxygen atom (**13***vs***14**, **16***vs***17**, and **20***vs***21**, Scheme 1 and Table 1) showed that, in all cases, the compound bearing an oxygen atom was less potent; these results are in line with those previously found within the adamantane series of sEHIs.^19^ Interestingly, the AR9281 analogues **16** and **17** showed the largest difference (300-fold difference, Table 1). Also, the replacement of the methyl group at the R position of the polycyclic scaffold by a hydrogen atom in the AR9281 analogues produced a dramatic drop of the inhibitory activity (**17***vs***18**, >10-fold decrease in potency, Table 1).

Considering the metabolism liability of the adamantane and adamantane-related scaffolds,^21,22^ we evaluated the *in vitro* stability in human, mice, and rat microsomes of the new ureas bearing the benzohomoadamantane moiety (Table 1). Within the trifluorophenyl series, the substitution of the adamantane nucleus by the benzohomoadamantane scaffold showed an important decrease of the microsomal stability (**7***vs***13**, Table 1). By contrast, in the corresponding oxa-analogue, **14**, the stability seemed to be restored in human, but marginally in mice and rat microsomes (**13***vs***14**, Table 1). Moreover, the analogue of AR9281, **16**, presented very high metabolic liability in all three species, as less than 1% of the compound remains after being incubated with microsomes for 60 min (AR9281 *vs***16**, Table 1). Finally, within the *t*-AUCB series, the replacement of the adamantane moiety by the benzohomoadamantane scaffold led to similar stability in human microsomes but to lower stability in mice and rat microsomes. Interestingly, in the oxa-analogue **21**, the stability was also maintained in mice microsomes (**20** and **21***vs t*-AUCB, Table 1). Although it seems that the ureas presenting the oxa-benzohomoadamantane moiety were more stable in microsomes, taking into account that all these derivatives (**14**, **17**, **18**, and **21**) were considerably less potent, this oxa-polycyclic scaffold was abandoned and only the ureas featuring the benzohomoadamantane core were further evaluated.

Overall, the *t*-AUCB family of compounds presented the most favorable properties in terms of potency and microsomal stability, and this series was selected for further optimization. As the adamantane nucleus contributes to the high lipophilicity of the known sEHI that compromises the solubility of these compounds, we next measured the solubility of the selected *t*-AUCB series in a 1% DMSO: 99% PBS buffer solution. As expected, the solubility decreases from the adamantane-based *t*-AUCB to the benzohomoadamantane analogue **20** (Table 2) likely due to the increase of carbon atoms from the adamantane nucleus (10 atoms) to the new polycyclic scaffold (16 atoms). Taking this into account, novel substitutions in the R position of the benzohomoadamantane scaffold were explored toward improving solubility while maintaining or enhancing both potency and microsomal stability of **20**. Thus, the methyl group of **20** was replaced by chlorine and fluorine atoms, leading to compounds **22** and **23**, respectively. The potency, microsomal stability, solubility, and permeability of both compounds were assessed in order to explore their properties (Table 2).

**Table 2:** IC_50_ in Human, Murine, and Rat sEH, Microsomal Stability, Solubility, and Permeability Values of the *t*-AUCB Related Compounds

							permeability (Caco-2)				
	sEH IC_50_ (nM)^a^					Papp (nm/s)					
Cpd	human	murine	rat	microsomal stability^b^ (h/m/r)	solubility^c^ (μM)	A→B	B→A	ER^d^	LD_50_^e^(μM)	IC_50_ *h*LOX-5^f^(μM)	IC_50_ *h*COX-2^g^(μM)
**4**, ***t***-**AUCB**	0.5	1.7	8.0^h^	94/92/46	25	1.9	210.3	111	ND^i^	ND	ND
**20**	0.9	9.9	0.4	70/10/2	4	10	123.7	12.4	>100	>100	>10
**22**	0.4	0.4	0.4	89/29/52	13	21.5	46.6	2.1	>100	>100	>10
**23**	0.5	0.5	0.4	77/36/60	7	0.9	219.1	243.9	>100	>100	>10

a Reported IC_50_ values are the average of three replicates. The fluorescent assay as performed here has a standard error between 10 and 20%, suggesting that differences of twofold or greater are significant. Because of the limitations of the assay, it is difficult to distinguish among potencies <0.5 Nm.^16^

b Percentage of remaining compound after 60 min of incubation with human, mice, and rat microsomes obtained from Tebu–Xenotech in the presence of NADP at 37 °C.

c Solubility in a 1% DMSO: 99% PBS buffer solution, see the Experimental Section for details.

d The efflux ratio was calculated as ER = (Papp B → A)/(Papp A → B). See the Experimental Section for further details.

e sEHI cytotoxicity tested by propidium iodide staining after 24 h of incubation in SH-SY5Y cells. See the Experimental Section for further details.

f IC_50_ in human LOX-5 (*h*LOX-5). See the Experimental Section for further details.

g IC_50_ in human COX-2 (*h*COX-2) performed by Eurofins (catalogue reference 4186).

h Taken from ref (24).

i ND: Not determined.

Satisfactorily, the evaluation of the inhibition activity against the hsEH showed that both compounds **22** and **23** were slightly more potent than **20**, with IC_50_ values in the same range as *t*-AUCB. Of note, **22** and **23** presented the same potency inhibiting the human, murine, and rat enzymes, while *t*-AUCB was three- and twenty-fold less potent with the murine and the rat enzymes, respectively (Table 2).

Regarding metabolic stability, compounds **22** and **23** presented better stabilities than **20** (Table 2). Furthermore, the experimental solubility values of these new halogenated compounds were determined. In line with a previous work with adamantane derivatives,^23^ the solubility increases when the methyl group is replaced by a halogen atom (compare **20***vs***22** and **23**, Table 2), particularly for the chlorinated compound **22**.

The Caco-2 cell permeability model was used in order to evaluate the permeability of the compounds. Apparent permeability values (Papp) were determined from the amount permeated through the Caco-2 cell membranes at both apical-basolateral (A-B) and basolateral-apical (B-A) direction. Gratifyingly, compounds **20** and **22** presented higher permeability values than *t*-AUCB, **22** being the one that presented the best profile (Table 2). Moreover, the cytotoxicities of **20**, **22**, and **23** were evaluated in SH-SY5Y cells by propidium iodide staining after 24 h of incubation. None of the compounds showed to be cytotoxic at the highest concentration tested (100 μM).

Finally, inhibitors **20**, **22**, and **23** were tested for selectivity against *h*COX-2 and *h*LOX-5, two enzymes involved in the AA cascade. Neither **20** nor the halogenated analogues **22** and **23** significantly inhibited these enzymes (see Table 2).

Next, considering the best permeability of **20** and **22**, both compounds were selected for CYPs and hERG inhibition assays. Cytochromes P450 (CYP) inhibition was evaluated using human recombinant cytochrome P450 enzymes CYP1A2, CYP2C9, CYP2C19, CYP2D6, and CYP3A, through a fluorescence-detection method. These assays were of great interest not only for the detection of possible drug–drug interactions but also in terms of selectivity as EETs are formed by several cytochrome P450 isoforms, particularly CYP2C19. Satisfactorily, the tested compounds did not significantly inhibit the evaluated cytochromes. We considered acceptable IC_50_ values around 2 μM in CYP2C19 taking into account that both compounds presented 2000-fold more potency inhibiting the sEH.

Regarding the hERG inhibition assay, both compounds inhibited the channel only 4 and 44% at 10 μM, respectively (Table 3). Overall, both compounds presented high potency inhibiting human, murine, and rat enzymes and did not significantly inhibit either cytochromes or hERG. Considering the biological profiling of the new sEH inhibitors, compounds **20** and **22** were selected as the candidates for the *in vivo* studies.

**Table 3:** Inhibition (Expressed as % of Inhibition at 10 μM or IC_50_) of Recombinant Human Cytochromes P450 Enzymes and Inhibition of the hERG Channel (Expressed as % of Inhibition at 10 μM)

	cytochrome inhibition^a^						
	CYP 1A2	CYP 2C9	CYP 2C19 (μM)	CYP 2D6	CYP 3A4^b^		
Cpd					(BFC)	(DBF)	hERG channel inhibition (% at 10 μM)
**20**	1 ± 2	17 ± 3	1.9	1 ± 1	2 ± 2	14 ± 1	4
**22**	14 ± 4	31 ± 3	2.2	11 ± 3	1 ± 2	52 ± 4	44

a The cytochrome inhibition was tested at 10 μM. IC_50_ was calculated for those compounds that presented >50% of inhibition.

b For the study of CYP3A4, two different substrates were used: benzyloxytrifluoromethylcoumarin (BFC) and dibenzylfluorescein (DBF).

### 2.3 *In silico* Study: Molecular Basis of Benzohomoadamantane-Derived Soluble Epoxide Hydrolase Inhibitors

The incorporation of an aromatic ring into the adamantane scaffold can potentially impact the orientation and molecular interactions of benzohomodamantane sEHIs compared to adamantane derivatives. To unravel how bulky benzohomodamantane ureas are accommodated in the active site of sEH and to understand the molecular basis of their inhibitory mechanism, molecular dynamics (MD) simulations were performed for compounds *t*-AUCB, **20**, **22**, and **23**. The MD simulations revealed that the addition of an aromatic ring onto the adamantane scaffold of *t*-AUCB triggers conformational rearrangements in the active site and adjacent regions to stabilize the benzohomoadamantane scaffold. These interactions, together with a network of hydrogen bonds and hydrophobic interactions with the urea and benzoic acid moieties, are key for retaining the inhibitors in the active site.

**Figure 4:**
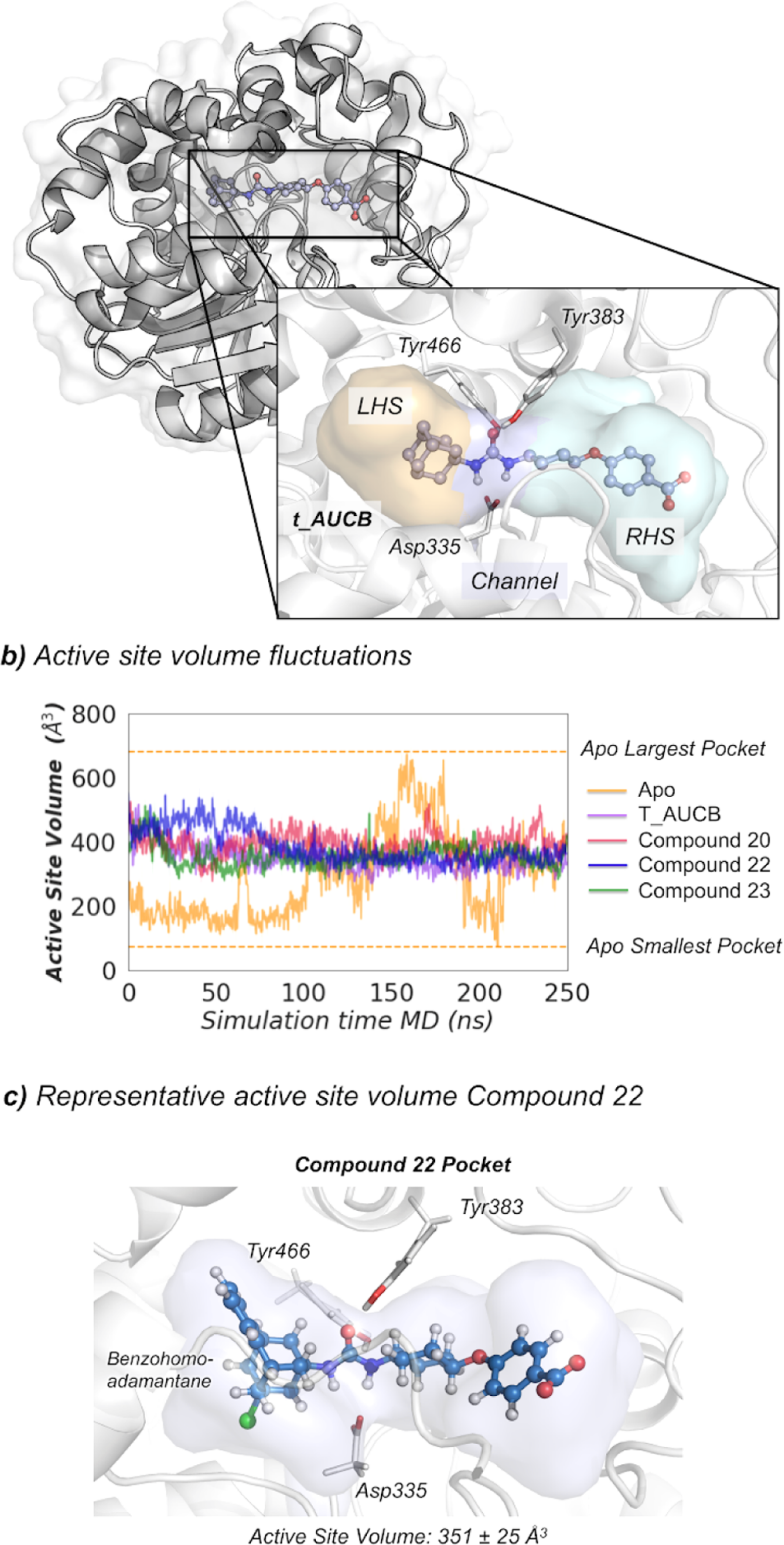
(a) Representation of the sEH structure (PDB: 5AM3), active site catalytic residues (nucleophilic Asp335, Tyr383, Tyr466), and *t*-AUCB inhibitor. The LHS pocket is colored in orange, the RHS pocket is colored in cyan, and the central channel in purple. (b) Plot of the fluctuations of the active site volume for the apo state (orange line, 290 ± 133 Å^3^), *t*-AUCB (purple line, 335 ± 33 Å^3^), compound **20** (red line, 396 ± 37 Å^3^), compound **22** (blue line, 351 ± 25 Å^3^), and compound **23** bound (green line, 356 ± 32 Å^3^) along a representative 250 ns MD simulation trajectory. The average volumes are calculated for the last 150 ns of the MD simulation. (c) Representative sEH structure with the active site volume obtained from MD simulations of compound **22**.

First, we explored the preferred binding mode of the selected sEHI and the flexibility of the active site of the sEH–inhibitor complex. The L-shaped active site pocket of sEH consists of three regions: the left-hand side (LHS) and the RHS pockets and a central narrow channel defined by catalytic residues Asp335, Tyr383, and Tyr466 that connects the LHS and RHS hydrophobic cavities (see Figure 4).^25^ Previously, we showed that the active site of EHs present high plasticity.^19,26^ Available X-ray structures of sEH in complex with adamantyl ureas indicate that the adamantane scaffold can occupy both LHS and RHS pockets.^27^ In the case of *t*-AUCB (PDB: 5AM3), the inhibitor is orientated with the benzoic acid group occupying the RHS, while adamantane sits in the LHS (see Figure 4). To corroborate that this is also the preferred orientation for benzohomoadamantane derivatives, we carried out molecular docking calculations for compounds **20**, **22**, and **23** (see the Experimental Section for details). All the binding poses featuring the urea moiety interacting with Asp335 oriented the benzohomoadamantane scaffold in the LHS and the benzoic acid in the RHS, as observed for *t*-AUCB. The LHS pocket presents enough space to accommodate the bulky benzohomoadamantane scaffold (see Figure 4). To evaluate the stability and molecular interactions of the inhibitor in the active site of sEH, we carried out three replicas of 250 ns MD simulations for *t*-AUCB, **20**, **22**, and **23**, starting from this orientation, that is the benzohomoadamantane occupying the LHS pocket. All inhibitors show considerable stability and no sign of unbinding or significant reorientations are observed along the MD simulation time. To evaluate the impact of the inhibitors on the active site conformational plasticity, we monitored the changes on the active site volume along the MD simulations (see Figures 4b and S1) using the POcket Volume MEasurer (POVME, see the Supporting Information for details).^28^ As observed previously, in the *apo* state, the total volume encompassing LHS, RHS, and the central channel displays wide fluctuations from 70 to 700 Å^3^ (average volume 290 ± 133 Å^3^).^19^ The size of the LHS pocket is mainly determined by flexible Ile363, Ile375, Met469, and Asn472 side chains, while the shape of the RHS pocket fluctuates with frequent side chain conformational changes of Leu408, Met419, and Phe497 residues (see Figure S1). When *t*-AUCB, **20**, **22**, and **23** compounds are bound in the active site, an expansion of the active site volume with respect to the average *apo* value is observed, which becomes stable at around 330–400 Å^3^ (see Figures 4b,c and S1). The average volumes determined for the last 150 ns of each MD simulation are 335 ± 33, 396 ± 37, 351 ± 25, and 356 ± 32 Å^3^ for *t*-AUCB, **20**, **22**, and **23**, respectively. As expected, benzohomadamantane inhibitors show wider active sites than *t*-AUCB,compound **20**, with a bulkier methyl group, being the one with the larger volume. The analysis of the root-mean-square fluctuation (RMSF) from the MD simulations shows that some active site residues become more rigid in the presence of inhibitors (see Figure S2). All inhibitors are able to restrict the conformational plasticity of the active site, indicating that they are tightly bound (see the population shift toward narrow distributions of active site volumes in Figure S1).

**Figure 5:**
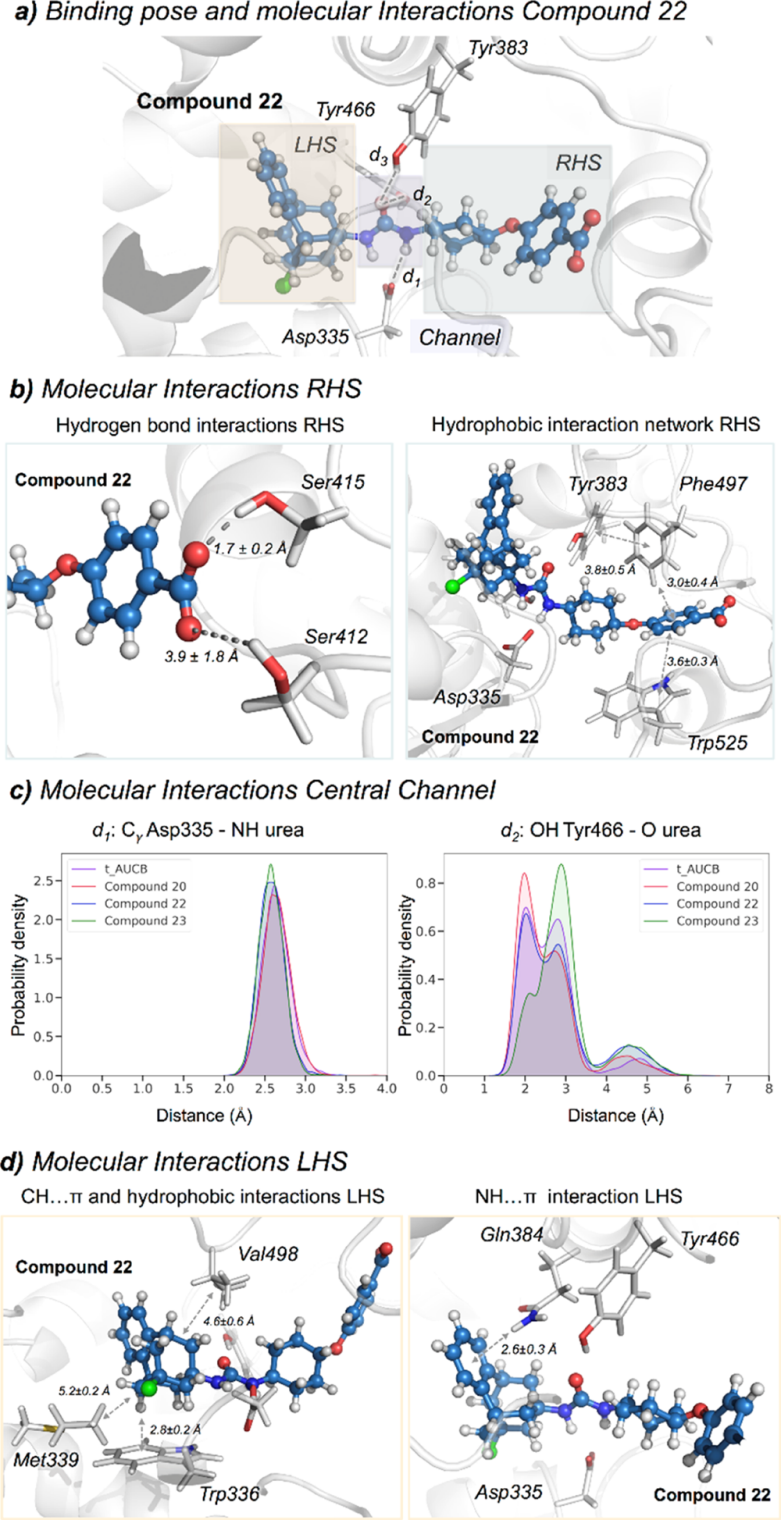
(a) Representative structure of **22** bound in the active site of sEH obtained from the most visited conformations along the MD simulations. The benzohomoadamantane moiety occupies the LHS pocket while the benzoic acid group lays in the RHS pocket. The central urea unit establishes hydrogen bonds with Asp335 (*d*_1_), Tyr466 (*d*_2_), and Tyr383 (*d*_3_). The PDB 5AM3 has been used as the starting point for all MD simulations. (b) Most relevant molecular interactions in the RHS. Average distances (in Å) obtained from the last 150 ns of MD simulations are represented. Hydrogen bonds between the oxygens of the carboxylate group of **22** and the hydrogen of the OH group of Ser412 and Ser415 are shown. The π–π stacking average distances are computed between the most proximal carbon atoms of each ring. The CH···π and hydrophobic interaction average distances are computed between the hydrogen atoms and the centroid of each aromatic ring (c) Histogram plots of the distance between the carboxylic group of the catalytic Asp335 and the amide groups of the inhibitor [*d*_1_(CγAsp335-NH_INH_)] and the distance between the carbonyl group of the urea inhibitor and the OH group of Tyr466 residue [*d*_2_(OHTyr466-O_INH_)] along the MD simulations of *t*-AUCB (purple), **20** (red), **22** (blue), and **23** (green). (d) Most relevant molecular interactions in the LHS. Average distances (in Å) obtained from the last 150 ns of MD simulations are represented. The CH···π interaction is calculated between the hydrogen of benzohomoadamantane unit and the centroid of the benzoid ring of Trp336. The NH···π interaction is monitored between the amide hydrogen of Gln384 and the center of the aromatic ring of the benzohomoadamantane scaffold.

To gain a deeper insight into the molecular basis of the inhibitory mechanism of benzohomoadamantane ureas, the non-covalent interactions between the selected inhibitors and the active site residues of sEH were analyzed with NCIplot on the most visited MD conformations (see Figures 5 and S3).^29^ First, we analyzed the interactions established in the RHS pocket and the central channel where all inhibitors share a common scaffold: the benzoic acid group. For *t*-AUCB, **20**, **22**, and **23** compounds, the carboxylate unit is stabilized by two hydrogen bonds with Ser412 and Ser415 that are located at the entrance of the RHS pocket, the interaction with Ser415 being more stable along the MD simulations (see Figure 5b and S3). The aromatic ring of the benzoic acid is further stabilized in the RHS pocket through CH···π interactions by the side chains of Trp525 and Phe497. The side chain of Phe497 transitions from the solvent to the active site to form a network of stable hydrophobic interactions that includes the benzoic acid group and the aromatic side chains of residues Trp525, Phe497, and catalytic Tyr383. Moreover, MD simulations show that four water molecules permanently occupy the RHS pocket in *t*-AUCB, **20**, **22**, and **23** (see Figures S4 and S5). These water molecules establish a network of interactions with the carboxylate group of the inhibitor and with residues Leu417, Ser407, Val416, Ser415, Ser412, and Lys495 that provides extra stabilization of the benzoic acid group in the RHS pocket. The urea moiety establishes hydrogen bonds with three catalytic residues: Asp335, Tyr383, and Tyr466 located in the central channel of the active site pocket. MD simulations show that the three hydrogen bonds remain significantly stable along the whole simulation time for all inhibitors with no significant differences (see Figure 5c). *t*-AUCB and **20** are able to retain a tighter hydrogen bond (below 3 Å) between the carbonyl of the urea and the OH of Tyr466 than halogenated compounds **22** and **23**. These results indicate that all inhibitors remain stable in the active site pocket, forming similar interactions consistent with reported IC_50_ values. Previously, we have shown that less potent inhibitors displayed fluctuations in the interactions between the urea motif and the catalytic residues, shifting the ensemble toward longer distances.^19^ All inhibitors share a common scaffold on the RHS of the urea and MD simulations reported a similar behavior in terms of interactions and conformational dynamics in the RHS and central channel regions. The network of hydrogen bonds and π–π stacking interactions is key to retain the inhibitor in the active site.

Significant differences were observed in the LHS pocket, where the benzohomoadamantane scaffold is placed. For *t*-AUCB, **20**, **22**, and **23**, the adamantane unit is mostly stabilized by the side chain of Trp336 through stable CH···π interactions (see Figure 5d and S3). In all cases, additional hydrophobic interactions with the side chains of Met339 and Val498 that wrap the adamantane in the LHS pocket are observed. The incorporation of an aromatic ring into the adamantane scaffold of *t*-AUCB induces a series of conformational rearrangements in the active site, which further stabilize both the adamantane and aromatic groups. In particular, the benzohomoadamantane scaffold is reoriented in the beginning of the MD simulations to position the aromatic ring toward the amide group of the side chain of Gln384 for establishing NH···π interactions that retain the benzohomoadamantane group fixed in the LHS pocket (see Figure 5d and S3). This interaction is observed in all MD simulations in the presence of **20**, **22**, and **23** inhibitors and, once formed, remains stable along the whole simulation time. In *t*-AUCB, the amide group of Gln384 forms a network of hydrogen bond interactions with the OH group of Tyr383 and the urea moiety that is partially disrupted in the presence of benzohomoadamantane. Additionally, Phe381 moves away from the LHS pocket to accommodate the aromatic rings of **20**, **22**, and **23**, establishing frequent π-stacking interactions. Met468, that in the *t*-AUCB X-ray structure is pointing toward the solvent, moves toward the active site to establish hydrophobic interactions with the benzohomoadamantane moiety. The hydrophobic nature of the LHS pocket prevents the permanence of water molecules in this cavity (see Figure S5). However, transient entrance of one or two water molecules is frequently observed along the MD simulations of **20**, **22**, and **23** compounds. Finally, the symmetric adamantyl unit in *t*-AUCB freely rotates inside the LHS pocket, while the asymmetry introduced in the benzohomoadamantane scaffold limits its rotation inside the active site (see Figure S6). The strong and stable hydrophobic and NH···π interactions significantly reduce the rotation of the benzohomoadamantane moiety inside the LHS pocket (see Figure S6). This limited flexibility can pose some impediments in the binding pathway of benzohomoadamante derivatives.

**Figure 6:**
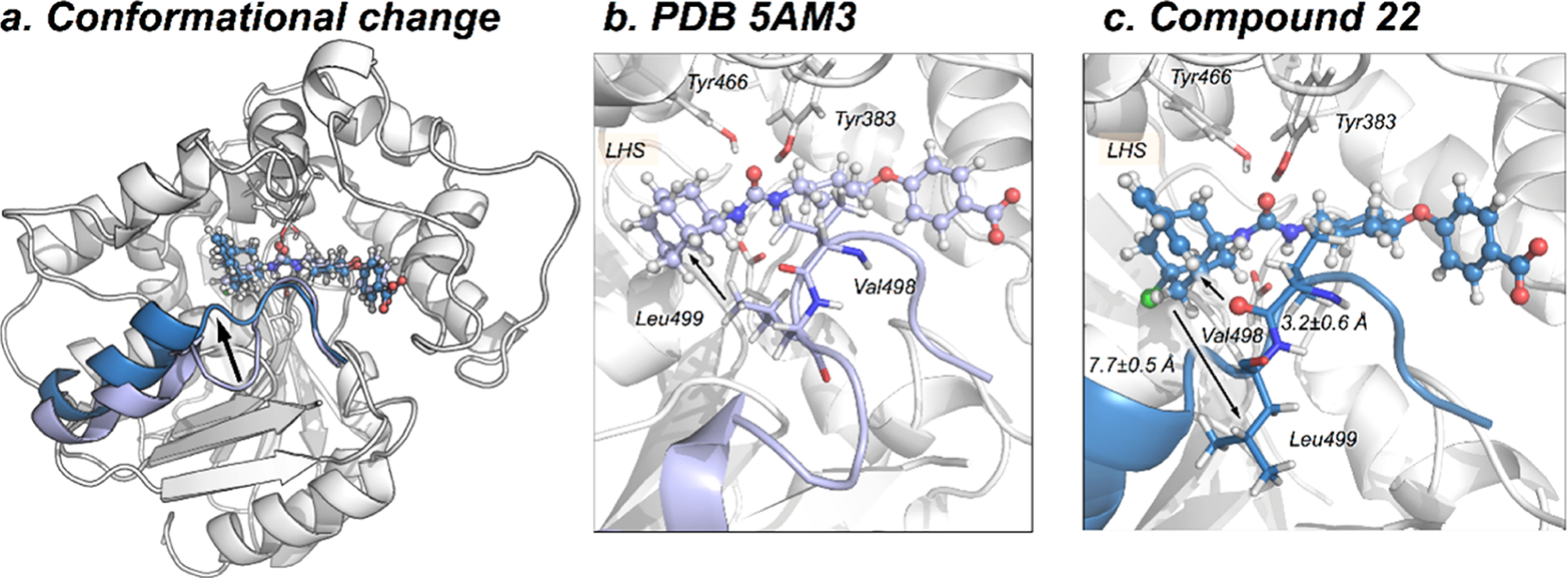
(a) Overlay of two representative structures of *t*-AUCB (purple, PDB 5AM3) and **22** (blue) bound in the active site of sEH. The image indicates the motion of the loop (493–500) containing Val498 and Leu499 with a black arrow. The loop is colored in purple for the X-ray *t*-AUCB conformation and in blue for the **22** conformation. The PDB 5AM3 have been used as the starting point for all MD simulations. (b) X-ray structure (PDB 5AM3), where Leu499 is pointing toward the adamantane moiety. (c) Most visited active site conformation with **22** bound, where Leu499 is displaced from the active site and Val498 carbonyl points toward the benzohomoadamantane moiety.

A significant conformational rearrangement is observed for compounds **22** and **23** in the course of the 250 ns of the MD simulation. The active site loop (493–500 residues) containing Leu499 is significantly displaced from the reference X-ray structure (see Figures 6 and RMSF S2). To explore whether this conformational change can take place in longer time-scales also for *t*-AUCB and **20**, we performed 500 ns of accelerated MD (aMD) simulations for all compounds.^30^ The analysis of aMD simulations confirms that this rearrangement occurs in the presence of all compounds (see Figure S7 and the Supporting Information for aMD details). This rearrangement includes the motion of the bulky Leu499 side chain that leaves the active site and the approximation of the carbonyl backbone of Val498 toward the benzohomoadamante moiety (see Figure 6b,c). Based on the analysis of NCIplot, the backbone of Val498 can establish dipole-induced dipole interactions generated by halogens F and Cl on the benzohomoadamantane scaffold. The computational mutagenesis of L499A and subsequent MD simulations on the L499A sEH variant showed that the loop is not perturbed along the simulation time, indicating that the displacement of the bulky side-chain of Leu499 can play a key role in accommodating the benzohomoadamantane scaffold. However, further investigations are required to explore the contribution of this conformational rearrangement in the thermodynamics and kinetics of inhibitor binding. Similar conformational changes in loops located at the vicinity of the active site have been described in other EH as key for substrate binding.^26^ The molecular insight gained from MD simulations paves the way toward the rational improvement of benzohomoadamantane scaffolds for enhanced inhibition.

### 2.4 Pharmacokinetic Studies of Selected Compounds **20** and **22**

The pharmacokinetic characterization of **20** and **22** was performed in male C57BL/6 mice by intraperitoneal administration of 3 mg/kg of each compound. As shown in Table 4, both compounds demonstrated good absorption and elimination characteristics. Notwithstanding, compound **22** presented a better profile than **20** considering its larger half-life (5.2 h), higher *C*_max_ (3583 ng/mL) and AUC (23,328.12 h*ng/mL), and lowest clearance (0.13 L/h/kg). Considering its superior pharmacokinetic profile and its better potency and solubility (Tables 2 and 4), **22** was selected for conducting the *in vivo* efficacy study in the well-known murine model of cerulein-induced acute pancreatitis (AP).^31−33^

**Table 4:** Pharmacokinetic Parameters in the Male C57BL/6 Mouse for Compounds **20** and **22** after 3 mg/kg IP Administration^a^

Cpd	dose (mg/Kg)	HL (h)	*T*_max_ (h)	*C*_max_ (ng/mL)	AUClast (h*ng/mL)	AUCINF (h*ng/mL)	*V*_d_ (L/Kg)	Cl (L/h/Kg)
**20**	3	1.17	0.50	1610	2260	2323	2.18	1.29
**22**	3	5.2	2	3583	22543	23328	0.96	0.13

a See the Experimental Section and Tables S2 and S3 and Figures S8 and S9 in the Supporting Information

### 2.5 *In vivo* Efficacy Study

AP is a potentially life-threatening gastrointestinal disease, and its incidence has been increasing over the last few decades. The onset of the disease is thought to be triggered by intra-acinar cell activation of digestive enzymes that results in interstitial edema, inflammation, and acinar cell death that often leads to a systemic inflammation response.^31−34^ The efficacy of the new sEHI **22** at 0.1 and 0.3 mg/kg was assessed in the cerulein-induced AP murine model. The experimental procedure for the *in vivo* efficacy study followed already published protocols.^35^

**Figure 7:**
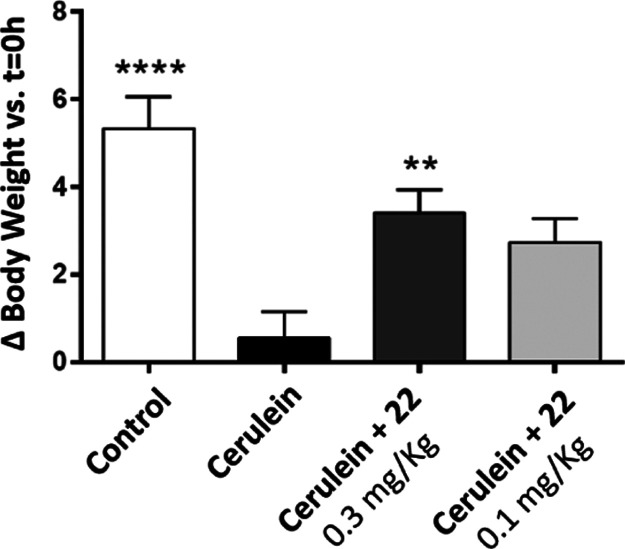
Percentage of body weight change at the end of the study *vs t* = 0 h. Effect of 12 consecutive administrations of cerulein (50 μg/kg, IP) and treatment with **22** (single dose, 0.3 or 0.1 mg/kg, IP) on C57BL/6 male mice body weight. Results are expressed as mean ± SEM (*n* = 3–9). *p < 0.05, **p < 0.01, ****p < 0.0001 *vs* Cerulein group (ANOVA-one way).

First, the health status of the animals was analyzed by monitoring their change in body weight along the experimental procedure. After food replacement (with the last cerulein injection), control animals gained some weight, and, as expected, it was not observed in animals receiving cerulein only. In contrast, animals treated with both doses (0.3 and 0.1 mg/kg) of **22** showed an increased body weight although only the group treated at 0.3 mg/kg reached statistical significance (*p* < 0.01 *vs* Cerulein group) (Figure 7).

In addition, the concentrations of **22** in plasma and pancreatic tissue were measured 10 h post-administration. Taking into account that **22** is a subnanomolar inhibitor of the murine sEH, we confirmed that the administration of both doses produced enough plasma levels of compound **22** to inhibit the sEH (175 nM for the dosage of 0.3 mg/kg and 14 nM for the dosage of 0.1 mg/kg). Moreover, the compound concentration in pancreas was also analyzed showing 29.9 ng/g for the dosage of 0.3 mg/kg, confirming that **22** was able to reach the pancreatic tissue. However, no amount of **22** was detected in pancreatic tissues with the 0.1 mg/kg dose.

**Figure 8:**
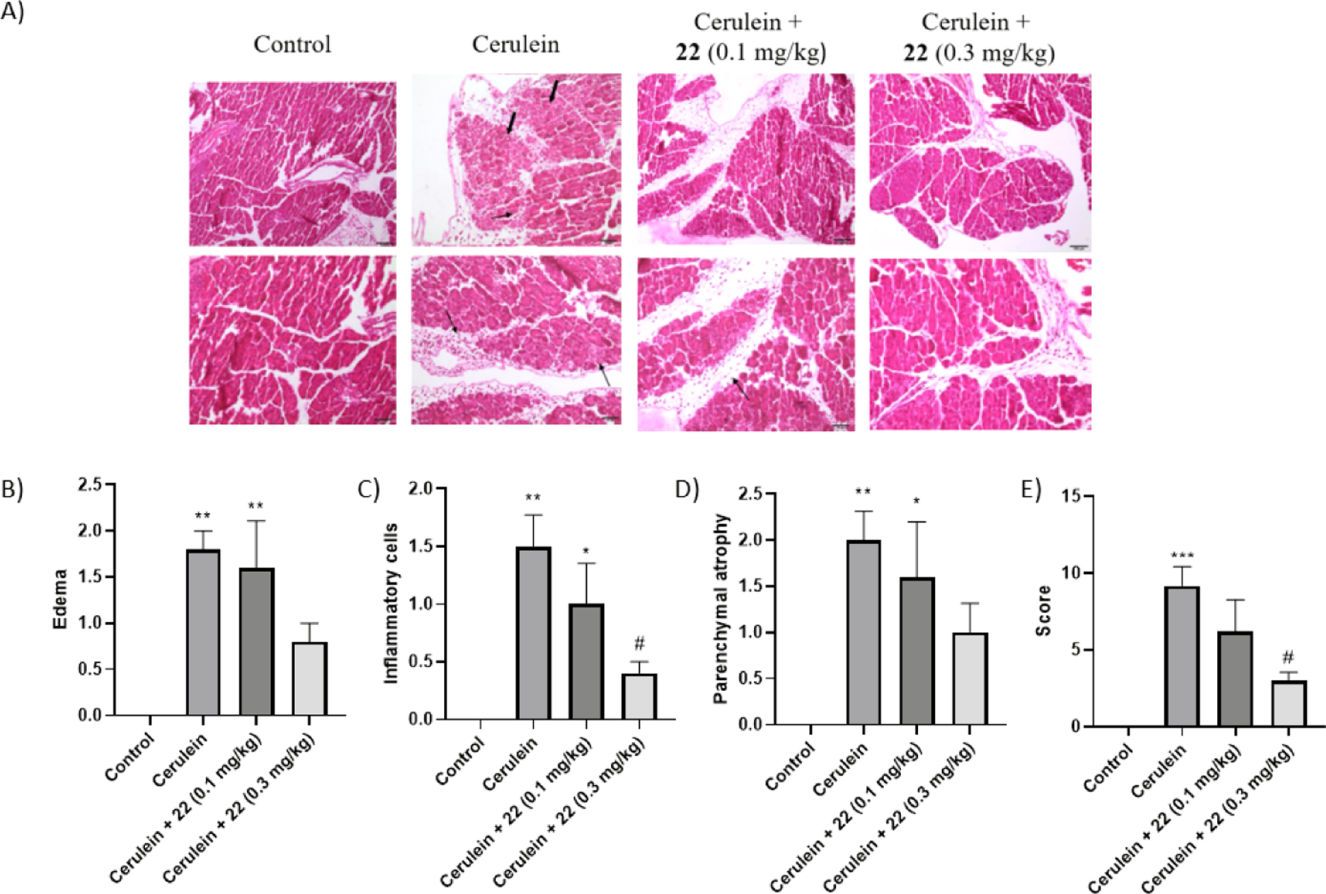
Results of the histologic analysis of pancreas from mice treated with vehicle (control), cerulein, and cerulein plus either 0.1 or 0.3 mg/kg of compound **22**. (A) Representative H&E-stained sections of the pancreas from the *in vivo* efficacy study. Arrows indicate inflammatory cells and edema. Bold arrows indicate intracellular vacuoles. (B–E) Histologic scoring of pancreatic tissues. (B), edema. (C), inflammatory cells (mononuclear and polymorphonuclear). (D), parenchymal atrophy. (E), total scoring (pancreatic parenchymal atrophy, vacuolar degeneration of cells, edema, hemorrhage, mononuclear inflammatory cells, mononuclear inflammatory cells, polimorfonuclear inflammatory cells and necrosis). *p < 0.05, **p < 0.01 and ***p < 0.001 *vs* control. ^#^*p* < 0.05 *vs* cerulein.

Finally, histologic analysis of pancreas was assessed in order to determine if treatment with **22** reduced the severity of the cerulein-induced pancreatitis. Pathologic changes were studied on H&E-stained pancreas sections (Figure 8A).

As expected, the cerulein control group presents pancreatic damage representative of AP, including edema (Figure 8B), infiltration of inflammatory cells (Figure 8C), and parenchymal atrophy (Figure 8D). By contrast, treatment with both doses of **22** ameliorated cerulein-induced effects. The higher dose (0.3 mg/kg) more efficiently reversed the pancreatic damage, edema, and neutrophil infiltration (Figure 8 and Tables S4–S7 in the Supporting Information).

## 3 Conclusions

The sEH has been identified as a suitable target for several inflammatory diseases. For this reason, many adamantane-based and aryl-based sEHIs have been designed and three selected compounds, AR9281, GSK2256294, and EC5026, have reached human clinical trials. In this work, we found that the aromatic and adamantane fragment can be merged, leading to the very versatile benzohomoadamantane scaffold. Therefore, three series of compounds replacing the adamantane moiety of AR9281, 7, and *t*-AUCB by this new polycycle have been synthesized and biologically evaluated. The *in vitro* profiling of these sEHIs (solubility, cytotoxicity, metabolic stability, CYP450s, *h*LOX-5, *h*COX-2 and hERG inhibition) allowed to select a suitable candidate for an *in vivo* efficacy study in a murine model of AP, for which sEHIs showed effectiveness at ameliorating this condition.^19,35,36^ The administration of **22** improved the general health status of cerulein-induced AP mice and significantly reduced pancreatic damage. Hence, the benzohomoadamantane moiety emerges as a suitable hydrophobic scaffold for the design of novel sEHIs. The molecular insight provided by MD simulations indicated that sEH reshapes the active site pocket to stabilize the aromatic ring of the benzohomoadamantane scaffold. Due to the promising results obtained with compound **22**, more research around benzohomoadamantane-based sEHIs for the treatment of inflammatory and pain-related diseases is currently ongoing.

## 4 Experimental Section

### 4.1 Chemical Synthesis

#### 4.1.1 General Methods

Commercially available reagents and solvents were used without further purification unless stated otherwise. Preparative normal phase chromatography was performed on a CombiFlash Rf 150 (Teledyne Isco) with pre-packed RediSep Rf silica gel cartridges. Thin-layer chromatography was performed with aluminum-backed sheets with silica gel 60 F254 (Merck, ref 1.05554), and spots were visualized with UV light and 1% aqueous solution of KMnO_4_. Melting points were determined in open capillary tubes with a MFB 595010M Gallenkamp. 400 MHz ^1^H and 100.6 MHz ^13^C NMR spectra were recorded on a Varian Mercury 400 or on a Bruker 400 AVANCE III spectrometers. 500 MHz ^1^H and 125.7 ^13^C NMR spectra were recorded on a Varian Inova 500 spectrometer. The chemical shifts are reported in ppm (δ scale) relative to internal tetramethylsilane, and coupling constants are reported in Hertz (Hz). Assignments given for the NMR spectra of selected new compounds have been carried out on the basis of DEPT, COSY ^1^H/^1^H (standard procedures), and COSY ^1^H/^13^C (gHSQC and gHMBC sequences) experiments. IR spectra were run on the PerkinElmer Spectrum RX I, PerkinElmer Spectrum TWO, or Nicolet Avatar 320 FT-IR spectrophotometers. Absorption values are expressed as wave-numbers (cm^–1^); only significant absorption bands are given. High-resolution mass spectrometry (HRMS) analyses were performed with an LC/MSD TOF Agilent Technologies spectrometer. The elemental analyses were carried out in a Flash 1112 series Thermofinnigan elemental microanalyzator (A5) to determine C, H, N, and S. The structure of all new compounds was confirmed by elemental analysis and/or accurate mass measurement, IR, ^1^H NMR and ^13^C NMR. The analytical samples of all the new compounds, which were subjected to pharmacological evaluation, possessed purity ≥95% as evidenced by their elemental analyses.

##### 4.1.1.1 1-(9-Methyl-5,6,8,9,10,11-hexahydro-7*H*-5,9:7,11-dimethanobenzo[9]annulen-7-yl)-3-(4-(trifluoromethyl)phenyl)thiourea (**9**)

To a solution of 9-methyl-5,6,8,9,10,11-hexahydro-7*H*-5,9:7,11-dimethanobenzo[9]annulen-7-amine hydrochloride (250 mg, 0.95 mmol) in DCM (2 mL), 1-isothiocyanato-4-(trifluoromethyl)benzene (193 mg, 0.95 mmol) and Et_3_N (287 mg, 2.84 mmol) were added. The reaction mixture was stirred at room temperature overnight and then the solvent was evaporated under vacuum. The residue was dissolved in EtOAc (30 mL) and water (20 mL) and the phases were separated. The aqueous phase was extracted with further EtOAc (2 × 30 mL). The combined organic phases were dried over anh. Na_2_SO_4_, filtered and concentrated under vacuum to obtain 369 mg of a yellow solid. The product was washed with Et_2_O to obtain thiourea **9** (188 mg, 46% yield) as a white solid, mp 158–159 °C. IR (NaCl disk): 3283, 2911, 2834, 1615, 1532, 1493, 1454, 1422, 1324, 120, 1166, 1124, 1067, 1015, 948, 909, 837, 759, 732, 697, 665 cm^–1^. ^1^H NMR (400 MHz, CDCl_3_): δ 0.95 (s, 3 H, C9–CH_3_), 1.57 [d, *J* = 13.6 Hz, 2 H, 10(13)-H_ax_], 1.67 (dd, *J* = 13.2 Hz, *J*′ = 6.0 Hz, 2 H, 10(13)-H_eq_], 2.00 (s, 2 H, 8-H), 2.31–2.40 [c.s., 4 H, 6(12)-H_2_], 3.12 [broad s, 2 H, 5(11)-H], 6.11 (s, 1 H, C7–NH), 7.05 [m, 2 H, 1(4)-H], 7.09 [m, 2 H, 2(3)-H], 7.28 [d, *J* = 8.2 Hz, 2 H, 2′(6′)-H], 7.64 [d, *J* = 8.2 Hz, 2 H, 3′(5′)-H], 7.81 (s, 1 H, C1′-NH). ^13^C NMR (100.5 MHz, CDCl_3_): δ 32.2 (CH_3_, C9–CH_3_), 33.9 (C, C9), 38.9 [CH_2_, C6(12)], 40.9 [CH, C5(11)], 41.1 [CH_2_, C10(13)], 47.5 (CH_2_, C8), 57.7 (C, C7), 123.7 (q, ^1^*J*_C-F_ = 272 Hz, C, CF_3_), 123.8 [CH, C2′(6′)], 126.5 [CH, C2(3)], 127.2 [q, ^3^*J*_C-F_ = 3.7 Hz, CH, C3′(5′)], 128.0 [CH, C1(4)], 128.1 (C, ^2^*J*_C-F_ = 33.1 Hz, CH, C4′) 140.1 (C, C1′), 145.8 [C, C4a(11a)], 178.2 (C, CS).

##### 4.1.1.2 *p*-Tolyl (9-Methyl-5,6,8,9,10,11-hexahydro-7*H*-5,9:7,11-dimethanobenzo[9]annulen-7-yl)carbamate (**11**)

To a solution of 9-methyl-5,6,8,9,10,11-hexahydro-7*H*-5,9:7,11-dimethanobenzo[9]annulen-7-amine hydrochloride (250 mg, 0.95 mmol) in DCM (2 mL), *p*-tolyl chloroformate (194 mg, 1.14 mmol) and Et_3_N (287 mg, 2.84 mmol) were added. The reaction mixture was stirred at room temperature overnight and then the solvent was evaporated under vacuum. The residue was dissolved in EtOAc (30 mL) and water (20 mL) and the phases were separated. The aqueous phase was extracted with further EtOAc (2 × 30 mL). The combined organic phases were dried over anh. Na_2_SO_4_, filtered, and concentrated under vacuum to obtain 300 mg of a yellow gum. Column chromatography (SiO_2_, hexane/ethyl acetate mixtures) gave carbamate **11** (46 mg, 14% yield) as a white solid, mp 114–115 °C. IR (NaCl disk): 3330, 3018, 2944, 2919, 2854, 1744, 1591, 1531, 1502, 1452, 1379, 1362, 1345, 1255, 1214, 1198, 1167, 1137, 1069, 1042, 1014, 987, 948, 900, 825, 757 cm^–1^. ^1^H NMR (400 MHz, CDCl_3_): δ 0.94 (s, 3 H, C9–CH_3_), 1.56 [d, *J* = 13.6 Hz, 2 H, 10(13)-H_ax_], 1.66 [dd, *J* = 13.6 Hz, *J*′ = 6.0 Hz, 2 H, 10(13)-H_eq_], 1.85 (s, 2 H, 8-H), 2.01 [d, *J* = 13.2 Hz, 2 H, 6(12)-H_ax_], 2.18 [dd, *J* = 13.2 Hz, *J*′ = 6.8 Hz, 2 H, 6(12)-H_eq_], 2.32 (s, 3 H, C4′-CH_3_), 3.10 [t, *J* = 5.6 Hz, 2 H, 5(11)-H], 4.92 (s, 1 H, NH), 6.97–7.00 [dm, *J* = 8.2 Hz, 2 H, 2′(6′)-H], 7.06 [cs, 2 H, 1(4)-H], 7.09 [c.s., 2 H, 2(3)-H], 7.12–7.14 [broad d, *J* = 8.2 Hz, 2 H, 3′(5′)-H]. ^13^C NMR (100.5 MHz, CDCl_3_): δ 20.8 (CH_3_, Ar-CH_3_), 32.2 (CH_3_, C9–CH_3_), 33.7 (C, C9), 39.3 [CH_2_, C6(12)], 40.9 [CH, C5(11)], 41.1 [CH_2_, C10(13)], 47.1 (CH_2_, C8), 53.8 (C, C7), 121.4 [CH, C2′(6′)], 126.3 [CH, C2(3)], 128.0 [CH, C1(4)], 129.6 [CH, C3′(5′)], 134.6 (C, C4′), 146.1 [C, C4a(11a)], 148.6 (C, C1′), 152.4 (C, CO).

##### 4.1.1.3 1-(9-Methyl-5,6,8,9,10,11-hexahydro-7*H*-5,9:7,11-dimethanobenzo[9]annulen-7-yl)-3-(2,3,4-trifluorophenyl)urea (**13**)

To a solution of 9-methyl-5,6,8,9,10,11-hexahydro-7*H*-5,9:7,11-dimethanobenzo[9]annulen-7-amine hydrochloride (193 mg, 0.73 mmol) in anh. DCM (6.5 mL) were added 2,3,4-trifluorophenyl isocyanate (105 mg, 0.61 mmol) and triethylamine (246 mg, 2.43 mmol) under a nitrogen atmosphere. The reaction mixture was stirred at room temperature overnight and the solvent was evaporated under vacuum. Column chromatography (SiO_2_, hexane/EtOAc mixture) of the crude and concentration under vacuum of the appropriate fractions gave urea **13** (38 mg, 13% yield) as a white solid, mp 206–207 °C. IR (ATR) ν: 3331, 2903, 2839, 1654, 1556, 1510, 1473, 1361, 1344, 1290, 1237, 1174, 1101, 1038, 1019, 1004, 800, 756, 690, 669, 625 cm^–1^. ^1^H NMR (500 MHz, CD_3_OD): δ 0.94 (s, 3 H, C9–CH_3_), 1.50 [d, *J* = 13.5 Hz, 2 H, 10(13)-H_ax_], 1.69 [m, 2 H, 10(13)-H_eq_], 1.77 (s, 2 H, 8-H), 2.10 [m, 2 H, 6(12)-H_eq_], 2.15 [d, *J* = 13 Hz, 2 H, 6(12)-H_ax_], 3.08 [tt, *J* = 6 Hz, *J’* = 1.5 Hz, 2 H, 5(11)-H], 6.98 (m, 1 H, 5′-H), 7.04 [broad s, 4 H, 1(4)-H and 2(3)-H], 7.66 (m, 1 H, 6′-H). ^13^C NMR (125.7 MHz, CD_3_OD): δ 32.9 (CH_3_, C9–CH_3_), 34.6 (C, C9), 40.6 [CH_2_, C6(12)], 42.5 [CH, C5(11)], 42.5 [CH_2_, C10(13)], 49.0 (CH_2_, C8), 54.5 (C, C7), 112.2 (CH, dd, ^2^*J*_C-F_ = 17.8 Hz, ^3^*J*_C-F_ = 3.9 Hz, C5′), 116.6 (CH, C6′), 127.0 (C, dd, ^2^*J*_C-F_ = 8.0 Hz, ^3^*J*_C-F_ = 2.4 Hz Ar-C1′), 127.4 [CH, C2(3)], 129.0 [CH, C1(4)], 141.0 (C, dt, ^1^*J*_C-F_ = 247.8 Hz, ^2^*J*_C-F_ = 14.9 Hz, Ar-C3′), 143.6 (C, dd, ^1^*J*_C-F_ = 245.7 Hz, ^2^*J*_C-F_ = 12.8 Hz, Ar-C4′), 147.3 (C, dd, ^1^*J*_C-F_ = 242.6 Hz, ^2^*J*_C-F_ = 10.3 Hz, Ar-C2′), 147.6 [C, C4a(C11a)], 156.1(C, CO). MS (DIP), *m*/*z* (%); significant ions: 400 (M^+^, <1), 253 (19), 228 (14), 211 [(C_16_H_19_)^+^, 16], 172 (23), 155 (54), 149 (56), 148 (100), 147 (52), 143 (22), 141 (20), 129 (21), 128 (18), 115 (16).

##### 4.1.1.4 1-(5-Methyl-1,5,6,7-hexahydro-1,5:3,7-dimethanobenzo[*e*]oxonin-3(2*H*)-yl)-3-(2,3,4-trifluorophenyl)urea (**14**)

To a solution of 5-methyl-1,5,6,7-tetrahydro-1,5:3,7-dimethanobenzo[*e*]oxonin-3(2*H*)-amine hydrochloride (250 mg, 0.94 mmol) in anh. DCM (8.5 mL) were added 2,3,4-trifluorophenyl isocyanate (135 mg, 0.78 mmol) and triethylamine (316 mg, 3.13 mmol) under a nitrogen atmosphere. The reaction mixture was stirred at room temperature overnight and the solvent was evaporated under vacuo to furnish pure urea **14** as a white solid (205 mg, 54% yield), mp 257–259 °C. IR (ATR) ν: 3295, 3241, 3118, 2916, 2173, 1693, 1620, 1564, 1510, 1493, 1468, 1462, 1356, 1345, 1320, 1302, 1286, 1273, 1254, 1229, 1210, 1181, 1167, 1111, 1091, 1074, 1049, 1035, 1008, 999, 958, 906, 820, 812, 763, 646 cm^–1^. ^1^H NMR (400 MHz, DMSO-*d*_6_): δ 1.18 (s, 3 H, C5–CH_3_), 1.56 [d, *J* = 13.6 Hz, 2 H, 6(13)-H_b_], 1.84 [m, 2 H, 6(13)-H_a_], 1.97 [d, *J* = 13.2 Hz, 2 H, 2(12)-H_b_], 2.20 [m, 2 H, 2(12)-H_a_], 3.16 [t, *J* = 5.5 Hz, 2 H, 1(7)-H], 4.06 (s, 1 H, C3–NH), 7.14 (complex signal, 5 H, 8(11)-H, 9(10)-H, 5′-H), 7.84 (m, 1 H, 6′-H), 8.52 (broad s, 1 H, C1′-NH). ^13^C NMR (100.6 MHz, DMSO-*d*_6_): δ 31.1 (CH_3_, C5–CH_3_), 37.4 [CH_2_, C2(12)], 38.1 [CH_2_, C6(13)], 38.2 [CH, C1(7)], 73.4 (C, C5), 82.7 (C, C3), 111.6 (CH, dd, ^2^*J*_C-F_ = 17.2 Hz, ^3^*J*_C-F_ = 3.5 Hz, C5′), 114.3 (CH, broad s, C6′), 126.0 (C, dd, ^2^*J*_C-F_ = 7.8 Hz, ^3^*J*_C-F_ = 3.0 Hz, C1′), 126.5 [CH, C9(10)], 128.2 [CH, C8(11)], 139.0 (C, dd, ^1^*J*_C-F_ = 246 Hz, ^2^*J*_C-F_ = 15 Hz, C3′), 141.0 (C, dd, ^1^*J*_C-F_ = 248 Hz, ^2^*J*_C-F_ = 12 Hz, C4′), 144.7 (C, dd, ^1^*J*_C-F_ = 241 Hz, ^2^*J*_C-F_ = 11 Hz, C2′),145.5 [C, C7a(C11a)], 152.3 (C, CO). MS (DIP), *m*/*z* (%); significant ions: 402 (M^+^, 48), 171 (13), 170 (34), 169 (21), 157 (20), 156 (18), 155 (53), 154 (14), 153 (11), 148 (18), 147 [(C_6_H_4_F_3_N)^+^, 100], 146 (53), 145 (15), 143 (25), 142 (21), 141 (23), 131 (12), 130 (15), 129 (65), 128 (46), 127 (22), 116 (12), 115 (55), 91 (17), 84 (19), 83 (28), 71 (15), 70 (16), 69 (21). HRMS-ESI^+^*m*/*z*: [*M* + H]^+^ calcd for [C_22_H_21_F_3_N_2_O_2_+H]^+^, 403.1633; found, 403.1631.

##### 4.1.1.5 1-(1-Acetylpiperidin-4-yl)-3-(9-methyl-5,6,8,9,10,11-hexahydro-7*H*-5,9:7,11-dimethanobenzo[9]annulen-7-yl)urea (**16**)

To a solution of 9-methyl-5,6,8,9,10,11-hexahydro-7*H*-5,9:7,11-dimethanobenzo[9]annulen-7-amine hydrochloride (180 mg, 0.68 mmol) in DCM (3 mL) and saturated aqueous NaHCO_3_ solution (2 mL), triphosgene (102 mg, 0.34 mmol) was added. The biphasic mixture was stirred at room temperature for 30 min and then the two phases were separated and the organic layer was washed with brine (5 mL), dried over anh. Na_2_SO_4_, filtered, and evaporated under vacuum to obtain 1–2 mL of a solution of isocyanate in DCM. To this solution were added 1-(4-aminopiperidin-1-yl)ethan-1-one hydrochloride (122 mg, 0.68 mmol) and Et_3_N (138 mg, 1.36 mmol). The mixture was stirred overnight at room temperature, diluted with further DCM (10 mL), and washed with 2N NaOH solution (2 × 10 mL). Organics were dried over anh. Na_2_SO_4_, filtered, and concentrated under vacuum to obtain a yellow oil (232 mg). Column chromatography (SiO_2_, DCM/methanol mixtures) gave urea **16** as a white solid (143 mg, 53% yield). The analytical sample was obtained by crystallization from hot EtOAc (113 mg), mp 206–207 °C. IR (NaCl disk): 3359, 3065, 3016, 2938, 2906, 2860, 1644, 1620, 1555, 1493, 1452, 1360, 1344, 1319, 1267, 1228, 1212, 1136, 1090, 1049 cm^–1^. ^1^H NMR (400 MHz, CDCl_3_): δ 0.90 (s, 3 H, C9–CH_3_), 1.13 (dq, *J* = 12.0 Hz, *J*′ = 4.0 Hz, 1 H, 3′-H_ax_ or 5′-H_ax_), 1.20 (dq, *J* = 12.0 Hz, *J*′ = 4.0 Hz, 1 H, 5′-H_ax_ or 3′-H_ax_), 1.52 [d, *J* = 13.2 Hz, 2 H, 10(13)-H_ax_], 1.62 [dd, *J* = 6 Hz, *J*′ = 12.8 Hz, 2 H, 10(13)-H_eq_], 1.80 (s, 2 H, 8-H), 1.85 (m, 1 H, 3′-H_eq_ or 5′-H_eq_), 1.93 [d, *J* = 12.8 Hz, 2 H, 6(12)-H_ax_], 2.01 (m, 1 H, 5′-H_eq_ or 3′-H_eq_), 2.06 (s, 3 H, COCH_3_), 2.12 [dd, *J* = 12.8 Hz, *J*′ = 6.0 Hz, 2 H, 6(12)-H_eq_], 2.70 (m, 1 H, 6′-H_ax_ or 2′-H_ax_), 3.02–3.14 [complex signal, 3 H, 5(11)-H, 2′-H_ax_ or 6′-H_ax_], 3.68–3.78 (complex signal, 2 H, 4′-H, 2′-H_eq_ or 6′-H_eq_), 4.41 (dm, *J* = 13.6 Hz, 1 H, 6′-H_eq_ or 2′-H_eq_), 4.62–4.68 (complex signal, 2 H, C7–NH and C4′-NH), 7.02 [m, 2 H, 1(4)-H], 7.06 [m, 2 H, 2(3)-H]. ^13^C NMR (100.5 MHz, CDCl_3_): δ 21.4 (CH_3_, COCH_3_), 32.3 (CH_3_, C9–CH_3_), 32.4 (CH_2_, C3′ or C5′), 33.6 (CH_2_, C5′ or C3′), 33.7 (C, C9), 39.9 [CH_2_, C6(12)], 40.7 (CH_2_, C6′ or C2′), 41.1 [CH, C5(11)], 41.2 [CH_2_, C10(13)], 45.4 (CH_2_, C2′ or C6′), 46.7 (CH, C4′), 48.0 (CH_2_, C8), 53.4 (C, C7), 126.2 [CH, C2(3)], 128.0 [CH, C1(4)], 146.3 [C, C4a(11a)], 156.4 (C, NHCONH), 169.0 (C, COCH_3_).

##### 4.1.1.6 1-(1-Acetylpiperidin-4-yl)-3-(5-methyl-1,5,6,7-tetrahydro-1,5:3,7-dimethanobenzo[*e*]oxonin-3(2*H*)-yl)urea (17)

To a solution of 5-methyl-1,5,6,7-tetrahydro-1,5:3,7-dimethanobenzo[*e*]oxonin-3(2*H*)-amine hydrochloride (180 mg, 0.68 mmol) in DCM (3 mL) and saturated aqueous NaHCO_3_ solution (2 mL), triphosgene (102 mg, 0.34 mmol) was added. The biphasic mixture was stirred at room temperature for 30 min and then the two phases were separated and the organic one was washed with brine (5 mL), dried over anh. Na_2_SO_4_, filtered, and evaporated under vacuum to obtain 1–2 mL of a solution of isocyanate in DCM. To this solution were added 1-(4-aminopiperidin-1-yl)ethan-1-one hydrochloride (122 mg, 0.68 mmol) and Et_3_N (139 mg, 1.37 mmol). The mixture was stirred overnight at room temperature, diluted with further DCM (10 mL), and washed with 2N NaOH solution (2 × 10 mL). The organic layer was dried over anh. Na_2_SO_4_, filtered, and concentrated under vacuum to obtain a yellow residue (206 mg). Column chromatography (SiO_2_, DCM/methanol mixtures) furnished urea **17** as a white solid (135 mg, 49% yield). The analytical sample was obtained by crystallization from hot EtOAc (112 mg), mp 208–209 °C. IR (NaCl disk): 3357, 3054, 3012, 2969, 2926, 2853, 1646, 1611, 1546, 1492, 1450, 1358, 1324, 1268, 1222, 1156, 1101, 1088, 1035, 1212, 991, 947, 918, 900, 866, 829, 760, 733, 699 cm^–1^. ^1^H NMR (400 MHz, CDCl_3_): δ 1.26 (s, 3H, C9–CH_3_), 1.32–1.42 (complex signal, 2 H, 3′-H_ax,_ 5′-H_ax_), 1.66–1.67 [complex signal, 4 H, 2-H_ax_, 12-H_ax_, 6(13)-H_ax_], 1.85–1.91 [m, 2 H, 6(13)-H_eq_], 1.97 (m, 1 H, 3′-H_eq_ or 5′-H_eq_), 2.08 (m, 1 H, 5′-H_eq_ or 3′-H_eq_), 2.10 (s, 3 H, COCH_3_), 2.24–2.34 (complex signal, 2 H, 2-H_eq_, 12-H_eq_), 2.95 (ddd, *J* = 3.2 Hz, *J*′ = 10.8 Hz, 1 H, 2′-Hax or 6′-Hax), 3.16–3.26 [complex signal, 3 H, 6′-H_ax_ or 2′-H_ax_, 1(7)-H], 3.72 (m, 1 H, 2′-H_eq_ or 6′-H_eq_), 3.89 (m, 1 H, 4′-H), 4.34 (m, 1 H, 2′-H_eq_ or 6′-H_eq_), 4.78 (s, 1 H, C3–NH), 6.34 (d, *J* = 7.5 Hz, 1 H, 4′-NH), 7.09–7.15 (complex signal, 4 H, 8-H, 9-H, 10-H, 11-H). ^13^C NMR (100.5 MHz, CDCl_3_): δ 21.4 (CH_3_, COCH_3_), 31.5 (CH_3_, C5–CH_3_), 32.1 (CH_2_, C3′or 5′), 33.2 (CH_2_, C5′ or C3′), 37.4 (CH_2_, C2 or C12), 37.6 (CH_2_, C12 o C2), 38.4 [CH_2_, C6 (13)], 38.8 [CH, C1(7)], 40.2 (CH_2_, C6′ or C2′), 45.0 (CH_2_, C2′ or C6′), 46.5 (CH, C4′), 74.6 (C, C5), 82.6 (C, C3), 126.94 (CH, C9 or C10), 126.96 (CH, C10 or C9), 128.38 (CH, C8 or C11), 128.43 (CH, C11 or C8), 144.70 (C, C7a or C11a), 144.75 (C, C11a or C7a), 156.6 (CO, NHCONH), 168.9 (C, COCH_3_).

##### 4.1.1.7 1-(1-Acetylpiperidin-4-yl)-3-(1,5,6,7-tetrahydro-1,5:3,7-dimethanobenzo[*e*]oxonin-3(2*H*)-yl)urea (18)

To a solution of 1,5,6,7-tetrahydro-1,5:3,7-dimethanobenzo[*e*]oxonin-3(2*H*)-amine hydrochloride (300 mg, 1.19 mmol) in DCM (6.5 mL) and saturated aqueous NaHCO_3_ solution (6.3 mL), triphosgene (131 mg, 0.44 mmol) was added. The biphasic mixture was stirred at room temperature for 30 min and then the two phases were separated and the organic one was washed with brine (5 mL), dried over anh. Na_2_SO_4_, filtered, and evaporated under vacuum to obtain 1–2 mL of a solution of isocyanate in DCM. To this solution was added 1-(4-aminopiperidin-1-yl)ethan-1-one (203 mg, 1.43 mmol). The mixture was stirred overnight at room temperature and the solvent was evaporated under vacuum. Column chromatography (SiO_2,_ DCM/methanol mixtures) gave urea **18** as a white solid (90 mg, 20% yield). The analytical sample was obtained by a crystallization from a hot ethyl acetate/mixture, mp 120–121 °C. IR (ATR): 3340, 2921, 1856, 1730, 1632, 1552, 1493, 1453, 1356, 1327, 1299, 1274, 1244, 1204, 1122, 1088, 1047, 1025, 993, 947, 970, 907, 801, 760, 729, 643 cm^–1^. ^1^H NMR (400 MHz, CDCl_3_): δ 1.33–1.45 (complex signal, 2 H, 3′-H_ax_, 5′-H_ax_), 1.68–1.84 [complex signal, 4 H, 2(12)-H_ax_, 6(13)-H_ax_], 1.97 (m, 1 H, 3′-H_eq_ or 5′-H_eq_), 2.05–2.13 (complex signal, 4 H, 5′-H_eq_ or 3′-H_eq_, COCH_3_), 2.21 [m, 2 H, 6(13)-H_eq_], 2.40 [m, 2 H, 2(12)-H_eq_], 2.87 (ddd, *J* = 11.2 Hz, *J*′ = 3.2 Hz, 1 H, 2′-H_ax_ or 6′ H-_ax_), 3.13–3.25 [complex signal, 3 H, 6′-H_ax_ or 2′-H_ax_, 1(7)-H], 3.74 (dm, *J* = 13.6 Hz, 1 H, 6′-H_eq_ or 2′-H_eq_), 3.90 (m, 1 H, 4′-H), 4.40 (dm, *J* = 13.2 Hz, 2′-H_eq_ or 6′-H_eq_), 4.52 (t, *J* = 5.6 Hz, 1 H, 5-H), 4.78 (s, 1 H, 3-NH), 6.14 (d, *J* = 7.6 Hz, 1 H, 4′-NH), 7.08–7.16 [complex signal, 4 H, 8-H, 9-H, 10-H, 11-H]. ^13^C NMR (100.5 MHz, CDCl_3_): δ 21.4 (CH_3_, COCH_3_), 32.2 (CH_2_, C3′ or C5′), 32.5 [CH_2_, C6(13)], 33.2 (CH_2_, C5′ or C3′), 38.0 (CH_2_, C2 or C12), 38.3 (CH_2_, C12 or C2), 38.65 (CH, C7 or C1), 38.69 (CH, C1 or C7), 40.4 (CH_2_, C2′ or C6′), 45.2 (CH_2_, C6′ or C2′), 46.7 (CH, C4′), 71.7 (CH, C5), 80.9 (C, C3), 126.91 (CH, C9 or C10), 126.93 (CH, C10 or C9), 128.47 (CH, C8 or C11), 128.52 (CH, C11 or C8), 145.0 (C, C7a or C11a), 145.1 (C11a or C7a), 156.5 (CO, NHCONH), 168.9 (CO, COCH_3_). HRMS: Calcd for [C_25_H_31_ClFN_3_O_2_+H]^+^; 460.2162; found, 460.2165.

##### 4.1.1.8 4-[((1*r*,4*r*)-4-(3-(9-Methyl-5,6,8,9,10,11-Hexahydro-7*H*-5,9:7,11-dimethanobenzo[9]annulen-7-yl)ureido)cyclohexyl)oxy]benzoic Acid (**20**)

To a solution of 9-methyl-5,6,8,9,10,11-hexahydro-7*H*-5,9:7,11-dimethanobenzo[9]annulen-7-amine hydrochloride (200 mg, 0.76 mmol) in DCM (3.5 mL) and saturated aqueous NaHCO_3_ solution (2.2 mL) was added triphosgene (113 mg, 0.38 mmol). The biphasic mixture was stirred at room temperature for 30 min and then the two phases were separated and the organic layer was washed with brine (5 mL), dried over anh. Na_2_SO_4_, filtered, and evaporated under vacuum to obtain 1–2 mL of a solution of isocyanate in DCM. To this solution were added 4-[((1*r*,4*r*)-4-aminocyclohexyl)oxy]benzoic acid hydrochloride (206 mg, 0.76 mg), Et_3_N (153 mg, 1.51 mmol), and DMF (5 mL). The mixture was stirred overnight at room temperature. The resulting suspension was evaporated, and the residue was suspended in DCM (20 mL) and washed with 2N HCl solution (2 × 10 mL). The resulting organic suspension was filtered, and the filtrate was dried over anh. Na_2_SO_4_, filtered, and concentrated under vacuum to give a white gum. Crystallization from hot EtOAc provided benzoic acid **20** as a white solid (55 mg, 16% yield), mp 182–183 °C. IR (NaCl disk): 3335, 2921, 2855, 1692, 1681, 1642, 1632, 1602, 1564, 1537, 1504, 1494, 1469, 1453, 1419, 1360, 1307, 1248, 1163, 1122, 1096, 1969 cm^–1^. ^1^H NMR (400 MHz, MeOD): δ 0.91 (s, 3 H, C9–CH_3_), 1.31 [m, 2 H, 3′(5′)-H_ax_], 1.47 [broad d, *J* = 13.2 Hz, 2 H, 10″(13″)-H_ax_], 1.53 [m, 2 H, 2′(6′)-H_ax_], 1.66 [dd, *J* = 12.8 Hz, *J′* = 6.4 Hz, 2 H, 10’’(13″)-H_eq_], 1.70 (s, 2 H, 8″-H), 1.96 [m, 2 H, 3′(5′)-H_eq_], 2.00–2.11 [complex signal, 6 H, 2′(6′)-H_eq_, 6″(12″)-H_2_], 3.05 [t, *J* = 5.6 Hz, 2 H, 5″(11″)-H], 3.48 (m, 1 H, 4′-H), 4.38 (m, 1 H, 1′-H), 6.95 [m, 2 H, 3(5)-H], 7.02–7.03 [complex signal, 4 H, 1’’(2′)-H, 3″(4″)-H], 7.93 [m, 2 H, 2(6)-H]. ^13^C NMR (100.5 MHz, MeOD): δ 31.2 [CH_2_, C2′(6′)], 31.7 [CH_2_, C3′(5′)], 32.9 (CH_3_, C9″-CH_3_), 34.5 (C, C9″), 41.0 [CH_2_, C6″(12″)], 42.6 [CH, C5″(11″)], 42.6 [CH_2_, C10″(13″) or CH, C5″(11″)], 48.7 (CH, C4′), 49.3 (CH_2_, C8″), 54.1 (C, C7″), 76.0 (CH, C1′), 116.1 [CH, C3(5)], 123.8 (C, C1), 127.3 [CH, C2″(3″)], 128.9 [CH, C1″(4″)], 132.9 [CH, C2(6)], 147.7 [C, C4a″(11a″)], 159.5 (C, NHCONH), 163.3 (C, C4), 169.8 (C, CO_2_H).

##### 4.1.1.9 4-[((1*r*,4*r*)-4-(3-(5-Methyl-1,5,6,7-tetrahydro-1,5:3,7-Dimethanobenzo[*e*]oxonin-3(2*H*)-yl)ureido)cyclohexyl)oxy]benzoic Acid (**21**)

To a solution of 5-methyl-1,5,6,7-tetrahydro-1,5:3,7-dimethanobenzo[*e*]oxonin-3(2*H*)-amine hydrochloride (200 mg, 0.75 mmol) in DCM (3.5 mL) and saturated aqueous NaHCO_3_ solution (2.2 mL) was added triphosgene (113 mg, 0.38 mmol). The biphasic mixture was stirred at room temperature for 30 min and then the two phases were separated and the organic layer was washed with brine (5 mL), dried over anh. Na_2_SO_4_, filtered, and evaporated under vacuum to obtain 1–2 mL of a solution of isocyanate in DCM. To this solution were added 4-[((1*r*,4*r*)-4-aminocyclohexyl)oxy]benzoic acid hydrochloride (206 mg, 0.76 mg), Et_3_N (153 mg, 1.52 mmol), and DMF (5 mL). The mixture was stirred overnight at room temperature. The resulting suspension was evaporated to obtain a white solid, which was suspended in DCM (20 mL) and washed with 2N HCl solution (2 × 10 mL). The resulting organic suspension was filtered to afford benzoic acid **21** as a white solid (200 mg, 54% yield), mp 220–222 °C. IR (NaCl disk): 3352, 2626, 1678, 1601, 1558, 1506, 1454, 1373, 1343, 1312, 1288, 1247, 1221, 1161, 1104, 1029, 997, 953, 776 cm^–1^. ^1^H NMR (400 MHz, DMSO): δ 1.14 (s, 3 H, C5–CH_3_), 1.30 [q, *J* = 11.6 Hz, 2 H, 3′(5′)-H_ax_], 1.41–1.54 (complex signal, 4 H, 2′(6′)-H_ax_, 6″(13″)-H_ax_], 1.76–1.87 [complex signal, 6 H, 3′(5′)-H_eq_, 2″(12″)-H_ax_, 6″(13″)-H_eq_], 2.01 [d, *J* = 9.2 Hz, 2 H, 2′(6′)-H_eq_], 2.19 [dd, *J* = 6 Hz, *J′* = 12.8 Hz, 2 H, 2″(12″)-H_eq_], 3.12 [t, *J* = 6.0 Hz, 1″(7″)-H], 3.41 (m, 1 H, 4′-H), 4.45 (m, 1 H, 1′-H), 5.97 (d, *J* = 7.6 Hz, 1 H, 4′-NH), 6.11 (s, 1 H, 3″-NH), 7.01 [d, *J* = 8.4 Hz, 2 H, 3(5)-H], 7.08–7.14 [complex signal, 4 H, 8″(11″)-H, 9″(10″)-H], 7.85 [d, *J* = 8.4 Hz, 2 H, 2(6)-H], 12.55 (broad s, 1 H, COOH). ^13^C NMR (100.5 MHz, DMSO): δ 29.5 [CH_2_, C2′(6′)], 30.1 [CH_2_, C3′(5′)], 31.2 (CH_3_, C5–CH_3_), 37.5 [CH_2_, C2″(12″)], 38.3 [2 signals, CH_2_, C6″(13″), and CH, C1″(7″)], 46.6 (CH, C4′), 73.0 (C, C5″), 74.2 (CH, C1′), 82.2 (C, C3″), 115.1 [CH, C3(5)], 122.6 (C, C1), 126.4 [CH, C9″(10″)], 128.1 [CH, C8″(11″)], 131.4 [CH, C2(6)], 145.6 (C, C7a″(11a″)], 155.8 (C, NHCONH), 161.1 (C, C4), 167.0 (C, CO_2_H). HRMS calcd for [C_29_H_35_N_2_O_5_+H]^+^; 491.254, found; 491.254.

##### 4.1.1.10 4-[((1*r*,4*r*)-4-(3-(9-Chloro-5,6,8,9,10,11-hexahydro-7*H*-5,9:7,11-dimethanobenzo[9]annulen-7-yl)ureido)cyclohexyl)oxy]benzoic Acid (**22**)

To a solution of 9-chloro-5,6,8,9,10,11-hexahydro-7*H*-5,9:7,11-dimethanobenzo[9]annulen-7-amine hydrochloride (180 mg, 0.63 mmol) in DCM (3 mL) and saturated aqueous NaHCO_3_ solution (2 mL), triphosgene (69 mg, 0.23 mmol) was added. The biphasic mixture was stirred at room temperature for 30 min and then the two phases were separated and the organic one was washed with brine (3 mL), dried over anh. Na_2_SO_4_, filtered, and evaporated under vacuum to obtain 1–2 mL of a solution of isocyanate in DCM. To this solution were added DMF (4 mL), 4-(((1*r*,4*r*)-4-aminocyclohexyl)oxy)benzoic acid hydrochloride (171 mg, 0.63 mmol), and Et_3_N (127 mg, 1.26 mmol). The mixture was stirred overnight at room temperature and the solvent was then evaporated. The residue was dissolved in DCM (5 mL) and washed with 2N HCl (3 mL). The organic phase was dried over anh. Na_2_SO_4_, filtered, and evaporated under vacuum to obtain benzoic acid **22** (217 mg, 67% yield) as a yellow residue. The analytical sample was obtained by crystallization from hot ethyl acetate/pentane mixtures, mp 201–202 °C. IR (ATR): 3355, 3299, 2932, 2856, 1697, 1682, 1631, 1605, 1555, 1498, 1469, 1452, 1428, 1406, 1373, 1357, 1322, 1301, 1253, 1163, 1100, 1077, 1041, 1027, 1013, 977, 946, 905, 844, 804, 772, 753, 695, 643, 634, 608 cm^–1^. ^1^H NMR (400 MHz, MeOD): δ 1.32 [m, 2 H, 3′(5′)-H_ax_], 1.54 [m, 2 H, 2′(6′)-H_ax_], 1.93–2.02 [complex signal, 4 H, 3′(5′)-H_eq_, 6″(12″)-H_ax_], 2.03–2.15 [complex signal, 4 H, 2′(6′)-H_eq_, 10″(13″)-H_ax_], 2.15–2.24 [m, 2 H, 6″(12″)-H_eq_], 2.35–2.41 [m, 2 H, 10″(13″)-H_eq_], 2.43 (s, 2 H, 8″-H), 3.17 [t, *J* = 6.0 Hz, 2 H, 5″(11″)-H], 3.49 (m, 1 H, 4′-H), 4.37 (m, 1 H, 1′-H), 6.95 [d, *J* = 8.6 Hz, 2 H, 3(5)-H], 7.04–7.12 [complex signal, 4 H, 1″(4″)-H, 2″(3″)-H], 7.94 [d, *J* = 8.6 Hz, 2 H, 2(6)-H]. ^13^C NMR (100.5 MHz, MeOD): δ 31.1 [CH_2_, C2′(6′)], 31.6 [CH_2_, C3′(5′)], 40.0 [CH_2_, C6″(12″)], 42.7 [CH, C5″(11″)], 46.0 [CH_2_, C10″(13″)], 48.7 (CH, C4″), 52.1 (CH, C8″), 56.3 (C, C7″), 70.5 (C, C9″), 76.0 (CH, C1′), 116.1 [CH, C5(3)], 123.9 (C, C1), 127.9 [CH, C2″(3″)], 129.1 [CH, C1″(4″)], 132.9 [CH, C2(6)], 146.3 [C, C4a″(11a″)], 159.2 (C, NHCONH), 163.2 (C, C4), the signal from CO_2_H was not observed. HRMS: Calcd for [C_29_H_33_ClN_2_O_4_–H]^−^; 507.2056, found; 507.2057.

##### 4.1.1.11 4-[((1*r*,4*r*)-4-(3-(9-Fluoro-5,6,8,9,10,11-hexahydro-7*H*-5,9:7,11-dimethanobenzo[9]annulen-7-yl)ureido)cyclohexyl)oxy]benzoic Acid (**23**)

To a solution of 9-fluoro-5,6,8,9,10,11-hexahydro-7*H*-5,9:7,11-dimethanobenzo[9]annulen-7-amine hydrochloride (180 mg, 0.67 mmol) in DCM (3 mL) and saturated aqueous NaHCO_3_ solution (2 mL), triphosgene (74 mg, 0.25 mmol) was added. The biphasic mixture was stirred at room temperature for 30 min and then the two phases were separated and the organic one was washed with brine (3 mL), dried over anh. Na_2_SO_4_, filtered, and evaporated under vacuum to obtain 1–2 mL of a solution of isocyanate in DCM. To this solution were added DMF (4 mL), 4-[((1*r*,4*r*)-4-aminocyclohexyl)oxy]benzoic acid hydrochloride (182 mg, 0.67 mmol), and Et_3_N (136 mg, 1.34 mmol). The mixture was stirred overnight at room temperature and the solvent was then evaporated. The residue was dissolved in DCM (5 mL) and washed with 2N HCl (3 mL). The organic phase was dried over anh. Na_2_SO_4_, filtered, and evaporated under vacuum to obtain benzoic acid **23** (240 mg, 72% yield) as a yellow residue. The analytical sample was obtained by crystallization from hot ethyl acetate/pentane mixtures, mp 253–254 °C. IR (ATR): 3325, 2929, 2859, 1682, 1629, 1606, 1558, 1511, 1424, 1359, 1317, 1282, 1251, 1221, 1165, 1104, 1090, 1003, 938, 851, 772, 697, 642 cm^–1^. ^1^H NMR (400 MHz, MeOD): δ 1.31 [dq, *J* = 3.2 Hz, *J*′ = 13.2 Hz, 2 H, 3′(5′)-H_ax_], 1.54 [dq, *J* = 3.2 Hz, *J′* = 12.8 Hz, 2 H, 2′(6′)-H_ax_], 1.81 [d, *J* = 12 Hz, 2 H, 10″(13″)-H_ax_)], 1.93–2.02 [complex signal, 4 H, 3′(5′)-H_eq_, 6″(12″)-H_ax_], 2.07–2.19 [complex signal, 8 H, 2′(6′)-H_eq_, 4-H, 6″(12″)-H_eq_, 10″(13″)-H_eq_], 3.23 [t, 2 H, *J* = 7.2 Hz, 5″(11″)-H], 3.49 (m, 1 H, 4′-H), 4.37 (m, 1 H, 1′-H), 6.95 [d, *J* = 8.8 Hz, 2 H, 3(5)-H], 7.09 [broad s, 4 H, 1″(4″)-H, 2″(3″)-H], 7.94 [d, *J* = 8.8 Hz, 2 H, 2(6)-H]. ^13^C NMR (100.5 MHz, MeOD): δ 31.1 [CH_2_, C2′(6′)], 31.6 [CH_2_, C3′(5′)], 40.4 [CH_2_, d, ^4^*J*_C-F_ = 1.9 Hz, C6″(12″)], 41.1 [CH, d, ^3^*J*_C-F_ = 13.3 Hz, C5″(11″)], 41.4 [CH_2_, d, ^2^*J*_C-F_ = 20 Hz, C10″(13″)], 47.9 (CH_2_, d, ^2^*J*_C-F_ = 17.9, C8″), 48.7 (CH, C4′), 57.6 (C, d, ^3^*J*_C-F_ = 11.2 Hz, C7″), 76.0 (CH, C1′), 95.4 (C, d, ^1^*J*_C-F_ = 177 Hz, C9″), 116.1 [CH, C3(5)], 123.9 (C, C1), 127.9 [CH, C2″(3″)], 129.1 [CH, C1″(C4″)], 132.8 [CH, C2(6)], 146.4 [C, C4a″(11a″)], 159.2 (C, NHCONH), 163.2 (C, C4), 169.8 (C, CO_2_H). HRMS: calcd for [C_29_H_33_FN_2_O_4_–H]^−^; 491.2352; found, 491.2334.

#### 4.1.2 Determination of IC_50_ sEHIs in Human, Murine, and Rat Purified sEH

IC_50_ is the concentration of a compound that reduces the sEH activity by 50%. The IC_50_ values reported herein were determined using a fluorescent-based assay (CMNPC as substrate).^16^ The fluorescent assay was used with purified recombinant human, mouse, or rat sEH proteins. The enzymes were incubated at 30 °C with the inhibitors ([I]_final_ = 0.4–100,000 nM) for 5 min in 100 mM sodium phosphate buffer (200 μL, pH 7.4) containing 0.1 mg/mL of BSA and 1% of DMSO. The substrate (CMNPC) was then added ([S]_final_ = 5 μM). Activity was assessed by measuring the appearance of the fluorescent 6-methoxynaphthaldehyde product (λ_ex_ = 330 nm, λ_em_ = 465 nm) every 30 s for 10 min at 30 °C on a SpectraMax M2 (Molecular Devices). Results were obtained by regression analysis from a linear region of the curve. All measurements were performed in triplicate and the mean is reported. *t*-TUCB, a classic sEHI, was run in parallel and the obtained IC_50_s were corroborated with reported literature values^37^ to validate the experimental results.

### 4.2 In silico Study

#### 4.2.1 MD Simulation Details

The parameters for *t*-AUCB, **20**, **22**, and **23** for the MD simulations were generated within the ANTECHAMBER module of AMBER 18^38^ using the general AMBER force field (GAFF),^39^ with partial charges set to fit the electrostatic potential generated at the HF/6-31G(d) level by the RESP model.^40^ The charges were calculated according to the Merz–Singh–Kollman scheme^41,42^ using Gaussian 09.^43^

MD simulations of sEH were carried out using PDB 5AM3 (crystallized with *t*-AUCB)^27^ as a starting point. For the MD simulations in the *apo* state, the *t*-AUCB was removed from the active site. The benzohomoadamanatane derivatives corresponding to **20**, **22**, and **23** were manually prepared using the *t*-AUCB structure as a starting point. Molecular docking calculations using the standard parameters of the SwissDock web server were carried out to assess the preferred orientation of **20**, **22**, and **23**.^44,45^ The coordinates of *t*-AUCB in PDB 5AM3 were a reference for placing compounds **20**, **22**, and **23** in molecular docking calculations. From these coordinates, conventional MD simulations were used to explore the conformational plasticity of sEH in the *apo* state and in the presence of *t*-AUCB, **20**, **22**, and **23** bound in the active site. All simulations were performed using the AMBER ff14SB force field.^46^ Amino acid protonation states were predicted using the H++ server (http://biophysics.cs.vt.edu/H++). The MD simulations have been carried with the following protonation of histidine residues: HIE146, HIE239, HIP251, HID265, HIP334, HIE420, HIE506, HIE513, HIE518, and HIP524.

Each system was immersed in a pre-equilibrated truncated octahedral box of water molecules with an internal offset distance of 10 Å. All systems were neutralized with explicit counterions (Na^+^ or Cl^–^). A two-stage geometry optimization approach was performed. First, a short minimization of the positions of water molecules with positional restraints on the solute by a harmonic potential with a force constant of 500 kcal mol^–1^ Å^–2^ was done. The second stage was an unrestrained minimization of all the atoms in the simulation cell. Then, the systems were gently heated in six 50 ps steps, increasing the temperature by 50 K each step (0–300 K) under constant-volume, periodic-boundary conditions, and the particle-mesh Ewald approach^47^ to introduce long-range electrostatic effects. For these steps, a 10 Å cutoff was applied to Lennard-Jones and electrostatic interactions. Bonds involving hydrogen were constrained with the SHAKE algorithm.^48^ Harmonic restraints of 10 kcal mol^–1^ were applied to the solute, and the Langevin equilibration scheme was used to control and equalize the temperature.^49^ The time step was kept at 2 fs during the heating stages, allowing potential inhomogeneities to self-adjust. Each system was then equilibrated for 2 ns with a 2 fs timestep at a constant pressure of 1 atm (NPT ensemble). Finally, conventional MD trajectories at a constant volume and temperature (300 K) were collected. In total, there were three replicas of 250 ns MD simulations for sEH in the apo state and in the presence of t-AUCB, **20**, **22**, and **23**, gathering a total of 3.75 μs of MD simulation time. Each MD simulation was clusterized based on active site residues, and the structures corresponding to the most populated clusters were used in the non-covalent interactions analysis. We monitored the presence of water molecules using the *watershell* function of the cpptraj MD analysis program.^50^

#### 4.2.2 Microsomal Stability

The human, rat, and mice recombinant microsomes employed were purchased from Tebu–Xenotech. The compound was incubated at 37 °C with the microsomes in a 50 mM phosphate buffer (pH = 7.4) containing 3 mM MgCl_2_, 1 mM NADP, 10 mM glucose-6-phosphate, and 1 U/mL glucose-6-phosphate-dehydrogenase. Samples (75 μL) were taken from each well at 0, 10, 20, 40, and 60 min and transferred to a plate containing 4 °C 75 μL of acetonitrile and 30 μL of 0.5% formic acid in water were added for improving the chromatographic conditions. The plate was centrifuged (46,000*g*, 30 min) and supernatants were taken and analyzed by an ultraperformance liquid chromatograph–tandem mass spectrometer (Xevo-TQD, Waters) by employing a BEH C18 column and an isocratic gradient of 0.1% formic acid in water: 0.1% formic acid acetonitrile (60:40). The metabolic stability of the compounds was calculated from the logarithm of the remaining compounds at each of the time points studied.

#### 4.2.3 Solubility

A 10 mM stock solution of the compound was serially diluted in 100% DMSO and 1 μL of this solution was added to a 384-well UV-transparent plate (Greiner) containing 99 μL of PBS. The plate was incubated at 37 °C for 2 h and the light scattering was measured in a Nephelostar Plus reader (BMG LABTECH). The data were fitted to a segmented linear regression for measuring the compound solubility.

#### 4.2.4 Permeability

The Caco-2 cells were cultured to confluency, trypsinized, and seeded onto a 96-filter transwell insert (Corning) at a density of ∼10,000 cells/well in DMEM cell culture medium supplemented with 10% fetal bovine serum, 2 mM l-glutamine, and 1% penicillin/streptomycin. Confluent Caco-2 cells were subcultured at passages 58–62 and grown in a humidified atmosphere of 5% CO_2_ at 37 °C. Following an overnight attachment period (24 h after seeding), the cell medium was replaced with fresh medium in both the apical and basolateral compartments every other day. The cell monolayers were used for transport studies 21 days post seeding. The monolayer integrity was checked by measuring the transepithelial electrical resistance (TEER), obtaining values ≥500 Ω/cm.^2^ On the day of the study, after the TEER measurement, the medium was removed and the cells were washed twice with pre-warmed (37 °C) Hank’s balanced salt solution (HBSS) buffer to remove traces of medium. Stock solutions were made in dimethyl sulfoxide (DMSO) and further diluted in HBSS (final DMSO concentration 1%). Each compound and reference compounds (Colchicine, E3S) were all tested at a final concentration of 10 μM. For A → B directional transport, the donor working solution was added to the apical (A) compartment and the transport media as receiver working solution was added to the basolateral (B) compartment. For B → A directional transport, the donor working was added to the basolateral (B) compartment and transport media as receiver working solution was added to the apical (A) compartment. The cells were incubated at 37 °C for 2 h with gentle stirring.

At the end of the incubation, samples were taken from both donor and receiver compartments and transferred into 384-well plates and analyzed by ultraperformance liquid chromatography–tandem mass spectrometry (UPLC–MS/MS). The detection was performed using an ACQUITY UPLC/Xevo TQD System. After the assay, Lucifer yellow was used to further validate the cell monolayer integrity, cells were incubated with LY 10 μM in HBSS for 1 h at 37 °C, obtaining permeability (Papp) values for LY of ≤10 nm/s, confirming the well-established Caco-2 monolayer.

#### 4.2.5 Cytotoxicity in SH-SY5Y Cells

Cytotoxicity was evaluated in the human neuroblastoma SH-SY5Y cell line (ATCC number: CRL-2266). Cells were cultured in minimum essential medium/Ham’s-F12 (1:1, v/v) medium, supplemented with non-essential amino acids, 10% fetal bovine serum, 1 mM glutamine, and 50 μg/mL gentamycin (all reagents from Gibco, Invitrogen). For experiments, the cells were seeded at 3 × 105 cells/mL (100 μL/well) in 96-well plates (Nunc). After 24 h, the testing compounds were added concentrated to triplicate wells to obtain the final different concentrations up to 100 μM. Compounds were incubated for further 24 h. At termination, cytotoxicity was analyzed by the propidium iodide (PI) fluorescence stain assay. All compounds were tested in three independent experiments using different cell passages. The PI assay measures cell death. PI enters into the cells with damaged membranes and greatly increases the fluorescence by binding to DNA. PI reagent (Molecular Probes) at the final concentration of 7.5 μg/mL was added to the cells and incubated for 1 h. The resulting fluorescence was measured by a Gemini XPS Microplate reader (Millipore) at 530 nm excitation and 645 nm emission. The percentage of cell death induced by the treatments was calculated from the fluorescence of treated cells (*F*_t_) relative to that of control cells (*F*_min_) and cells incubated with Triton X100 (*F*_max_) as the 0 and 100% cell death, respectively [% = ((*F*_t_ – *F*_min_)/(*F*_max_ – *F*_min_)) × 100].

#### 4.2.6 Inhibition of hLOX-5

AA and 2′,7′-dichlorodihydrofluorescein diacetate (H_2_DCFDA) were obtained from Sigma. Human recombinant LOX-5 was purchased from Cayman Chemical. For the determination of *h*LOX-5 activity, the method described by Pufahl *et al.* was followed.^51^ The assay solution consisted of 50 mM Tris (pH 7.5), 2 mM EDTA, 2 mM CaCl_2_, 3 μM AA, 10 μM ATP, 10 μM H_2_DCFDA, and 100 mU/well *h*LOX-5. For the enzyme inhibition studies, the compounds to be tested were added to the assay solution prior to AA and ATP and were preincubated for a period of 10 min at room temperature, after which AA and ATP were added. The enzymatic reaction was carried out for 20 min and terminated by the addition of 40 μL of acetonitrile. The fluorescence measurement, 485 nm excitation and 520 nm emission, was performed on a FLUOstar OPTIMA (BMG LABTECH, Offenburg, Germany.). The IC_50_ is defined as the concentration of compound that inhibits enzymatic activity by 50% over the untreated enzyme control.

#### 4.2.7 Cytochrome P450 Inhibition Assay

The objective of this study was to screen the inhibition potential of the compounds using recombinant human cytochrome P450 enzymes CYP3A4 (BFC and DBF substrates) and probe substrates with fluorescent detection. Incubations were conducted in a 200 μL volume in 96-well microtiter plates (COSTAR 3915). The addition of the mixture buffer-cofactor (KH_2_PO_4_ buffer, 1.3 mM NADP, 3.3 mM MgCl_2_, 0.4 U/mL glucose-6-phosphate dehydrogenase), control supersomes, the Standard inhibitor Ketoconazole (Sigma K1003), and previously diluted compounds to plates was carried out by a liquid handling station (Zephyr Caliper). The plate was then preincubated at 37 °C for 5 min in a 100 μL volume, and reaction was initiated by the addition of a prewarmed enzyme/substrate (E/S) mix. The E/S mix contained buffer (KH_2_PO_4_), enzyme (CYP), substrate 7-benzyloxytrifluoromethyl coumarin (7-BFC), and dibenzylfluorescein (DBF) in a reaction volume of 200 μL. Reactions were terminated after various times depending on the substrate by addition of STOP solution (ACN/TrisHCl 0.5 M 80:20 (BFC) or 2 N NaOH for CYP3A4 (DBF). Fluorescence per well was measured using a fluorescence plate reader (Tecan M1000 pro) and percentage of inhibition was calculated.

#### 4.2.8 hERG Inhibition Assay

The assay was carried out at a CHO cell line transfected with the hERG potassium channel. 72 h before the assay, 2500 cells were seeded on a 384-well black plate (Greiner 781091). The cell line was maintained at 37 °C in a 5% CO_2_ atmosphere for 24 h and at 30 °C in a 5% CO_2_ atmosphere for 48 h plus. hERG activity was measured by using the FluxorTM Potassium Ion Chanel Assay Kit (Thermo Fisher F10016). Medium was replaced for 20 μL of loading buffer and the cells were incubated for 60 min at RT, protected from direct light. After incubation, the loading buffer was replaced for the assay buffer and the compounds were incubated for 30 min at RT. 5 μL of a stimulus buffer was added to each well and the fluorescence was read (λ_ex_ = 490 nm, λ_em_ = 525 nm) using an imaging plate reader system (FDSS7000EX, Hamamatsu) every second after the establishment of a baseline line.

### 4.3 Pharmacokinetic Study

24 male C57BL/6 mice (21 g approx.) were supplied by Envigo (Barcelona, Spain) (Ref 15131). During the experimental procedure, animals were identified with a permanent marker (tail code numbers). Plasma samples were obtained at 0, 0.25, 0.5, 1, 2, 4, 6, and 24 h post-dosing. Upon arrival, animals were housed in groups of 3 animals/cage in polycarbonate maintenance cages (type IIL; 365 × 207 × 140 mm, with a surface area of 530 cm^2^) with absorbent bedding (Lignocel, JRS). Animals were kept in an environmentally controlled room (ventilation of 10–15 air changes per hour, temperature of 22 ± 3 °C, and humidity of 35–70%) on a 12 h light/dark cycle. They underwent a period of at least 5 days of observation and acclimatization between the date of arrival and the start of the procedure. During this period, the animals were observed to check their general health state. A maintenance diet was supplied by Harlan Interfauna Ibérica S.L. (2014 Harlan Teklad Global Diets) and was provided to the animals *ad libitum*. Diet was analyzed by the manufacturer to detect possible contaminants. Tap water was supplied by CASSA (Servei d’Aigües de Sabadell) and was provided to the animals by bottles *ad libitum*. The animals were maintained in accordance with the European Directive for the Protection of Vertebrate Animals Used for Experimental and other Scientific Purposes (86/609/EU). Decree 214/1997 of 30th July. Ministry of Agriculture, Livestock, and Fishing of the autonomous government of Catalonia, Spain. Royal Decree 53/2013 of 1st February (Spain) Animal care including environmental and housing conditions conformed to the applicable standard operating procedures regarding laboratory animals of Draconis Pharma S.L. All the experimental procedures were approved by the Animal Experimentation Ethical Committee of Universitat Autònoma de Barcelona (procedure number: 3718) and by the Animal Experimentation Commission of the Generalitat de Catalunya (Catalan Government) (DAAM:9590). Formulations were prepared on the day of the study. Vehicle was 10% of 2-hydroxypropyl-β-cyclodextrin, (CAS 128446-35-5) Sigma-Aldrich (Ref.332607-25G). 21 mice were intraperitoneally administered with a single dose of 3 mg/kg of **22**. The volume of administration was 10 mL/kg. Animals were weighed before each administration to adjust the required volume. Blood samples were collected at different times post administration: 0, 0.25, 0.5, 1, 2, 4, 6, and 24 h. Three mice were not administered and referred as *t* = 0. Blood samples (0.5–0.8 mL) were collected from anesthetized animals with isoflurane by cava vein puncture in tubes containing K2-EDTA 5%. Blood samples were centrifuged at 10,000 rpm for 5 min to obtain plasma that was stored at −20 °C until analysis. Analytical measurements were performed by LC–MS/MS. Pharmacokinetics parameters were calculated with Phoenix 64 (WinNonlin).

### 4.4 *In vivo* Efficacy Study

Forty-one male C57BL/6 mice (8-week-old; approximately 24 g) were supplied by Envigo (Barcelona, Spain) (Ref 16512). During the experimental procedure, the animals were identified with a permanent marker (tail code numbers). Upon arrival, the animals were housed in groups of 8–9 animals/cage in polysulfone maintenance cages (480 × 265 × 210 mm, with a surface area of 940 cm^2^) with wire tops and wood chip bedding. The animals were kept in an environmentally controlled room (ventilation, temperature 22 ± 2 °C, and humidity 35–65%) on a 12 h light/dark cycle. They underwent a period of 7 days of acclimatization between the date of arrival and the start of the procedure. During this period, the animals were observed to check their general health state. The maintenance diet was supplied by Harlan Interfauna Ibérica S.L. (2018 Harlan Teklad Global Diets). Diet was provided to the animals *ad libitum*, but they were fasted overnight before the first cerulein injection, and food was replaced after the last cerulein injection. Tap water was supplied by CASSA (Servei d’Aigües de Sabadell) *ad libitum*. The animals were maintained in accordance with the European Directive for the Protection of Vertebrate Animals Used for Experimental and other Scientific Purposes (86/609/EU). Decree 214/1997 of 30th July. Ministry of agriculture, livestock and fishing of the Autonomous Government of Catalonia, Spain. Royal Decree 53/2013 of 1st February (Spain). All the experimental procedures were approved by the Ethical Committee on human and animal experimentation (CEEAH) of Universitat Autònoma de Barcelona (UAB) (procedure number: 4107) and by the Animal Experimentation Commission of the Generalitat de Catalunya (Catalan Government) (DAAM: 10146). The test item was dissolved in vehicle 10% 2-hydroxypropyl-β-cyclodextrin (CAS 128446-35-5) Sigma-Aldrich (Ref 332607). The vehicle was prepared the day before and kept at 4 °C. Pancreatitis induction: Mice (*n* = 41) were weighed, identified by a distinct number at the base of the tail, and fasted overnight. Cerulein (cerulein and cerulein + **22** groups) (50 μg/kg, prepared in 0.9% NaCl) or vehicle (0.9% NaCl) (control group) were intraperitoneally injected (*V* = 5 mL/kg) 12 consecutive times, at 1 h intervals (*h* = 0–11). Food was replaced after the last injection. A satellite experiment was designed where animals (*n* = 3) were distributed in control, cerulein, and cerulein + **22**-treated groups. Pancreatitis was induced by seven injections of cerulein (or vehicle in the control group) at 1 h intervals (*h* = 0–6). Treatments: the test item was administered intraperitoneally in one injection to **22**_03 (0.3 mg/kg) and **22**_01 (0.1 mg/kg) groups at 14 h after the first cerulein injection. Animals from the control and cerulein groups received vehicle administration (10% 2-hydroxypropyl-β-cyclodextrin) (*V* = 10 mL/kg). Extra groups were treated 2 h after the first cerulein injection: **22** (0.3 mg/kg), control, and cerulein groups (10% 2-hydroxypropyl-β-cyclodextrin). Study end: 24 h after the first cerulein injection, animals were weighed and anesthetized with isoflurane. Blood was collected from the vena cava in an Eppendorf containing K2-EDTA and centrifuged at 10,000 rpm for 5 min for plasma collection. Plasma was stored at −80 °C until analysis. The mice were sacrificed by cervical dislocation and the pancreases were dissected and weighed. Pancreases from three animals were frozen in liquid N_2_ and stored at −80 °C until analysis. Pancreases from five mice were sectioned and one part was placed in 10% formalin and sent to Anapath (Granada, Spain) for histology analysis and the other was immediately placed in RNAse-free eppendorfs, frozen in N_2_, and stored at −80 °C for gene expression assays.

#### 4.4.1 Histologic Analysis

Pancreatic samples were treated with increasing grade alcohols and two xylol baths and embedded in paraffin. They were subsequently cut using a microtome and processed for staining. For the deparaffinization of the samples, two xylene baths (10 min) and three alcohols were used in decreasing solutions (100%, 90 and 70%) (5 min) and subsequently stained with hematoxylin (5 min) and eosin (5 min). During the dehydration process after staining with eosin, alcohols in increasing solution (70, 96, and 100%) and xylene were used again. Finally, the samples were mounted with DPX.

Histologic scoring of pancreatic sections was performed to grade the extent of pancreatic parenchymal atrophy, vacuolar degeneration of cells, edema, hemorrhage, mononuclear inflammatory cells, mononuclear inflammatory cells, polymorphonuclear inflammatory cells, and necrosis. The assigned scores were the following: 0 (no changes): when no lesions were observed or the observed changes were within normality; 1 (minimal): when changes were few but exceeded those considered normal; 2 (light): lesions were identifiable but with moderate severity; 3 (moderate): important injuries but they can still increase in severity; 4 (very serious): the lesions are very serious and occupy most of the analyzed tissue. The lesions were evaluated in the most affected lobes of all the pancreases. In the case of assessment of atrophy, it was determined based on the percentage of atrophied tissue as: 0 without atrophy; 1: 0–25% of atrophic parenchyma; 2: between 25 and 50%; 3: between 50 and 75% and 4: between 75 and 100%.

## Supporting Information Available

^1^H and ^13^C NMR spectra and elemental analysis data of the new compounds, HPLC trace for compound **22**, representation of the active site volume fluctuations, non-covalent interactions between the inhibitors and the active site residues of sEH, analysis of active site water molecules in MD simulations, plots of the dihedral angle that describes the rotation of the benzohomoadamantane and adamantane moieties in the left-hand-side pocket of the sEH active site along the MD simulations, overlay of the X-ray and representative snapshot from the apo state and inhibitor-bound aMD simulations, pharmacokinetic data of compounds **20** and **22**, and histologic scoring of pancreatic tissues of mice treated with **22** (PDF).
Molecular formula strings and data (CSV)

## Notes

S.C., E.V., and S.V. are inventors of the Universitat de Barcelona patent application on sEH inhibitors WO2019/243414. C.M. and B.D.H. are inventors of the University of California patents on sEH inhibitors licensed to EicOsis. None of the other authors has any disclosures to declare.

## Abbreviations USED

AP: acute pancreatitis
ATR: attenuated total reflectance
BEH: ethylene bridged hybrid
BFC: benzyloxytrifluoromethylcoumarin
CER: cerulein
COXs: cyclooxygenases
CYPs: cytochrome P450s
DBF: dibenzylfluorescein
DMPK: drug metabolism pharmacokinetics
EETs: epoxyeicosatrienoic acids
EtOAc: ethyl acetate
H&E: hematoxylin and eosin
hsEH: human soluble epoxide hydrolase
LHS: left-hand side
LOXs: lipoxygenases
ND: not determined
RHS: right-hand side
sEH: soluble epoxide hydrolase
sEHI: soluble epoxide hydrolase inhibitors.
