# Supplementary material for: From the Design to the In Vivo Evaluation of Benzohomoadamantane-Derived Soluble Epoxide Hydrolase Inhibitors for the Treatment of Acute Pancreatitis
Source: J Med Chem. 2021 May 4;64(9):5429–46. doi: 10.1021/acs.jmedchem.0c01601 (PMC8634379; doi:10.1021/acs.jmedchem.0c01601)
Supplement: Supplementary file 1 — jm0c01601_si_001.pdf [file jm0c01601_si_001.pdf]

SUPPORTING INFORMATION FOR

From the design to the *in vivo* evaluation of benzohomoadamantane-derived soluble epoxide hydrolase inhibitors for the treatment of acute pancreatitis

Sandra Codony[1], Carla Calvó-Tusell[2], Elena Valverde[1], Sílvia Osuna[2,3], Christophe Morisseau[4], *M. Isabel Loza[5], José Brea[5], Concepción Pérez[6], María Isabel Rodríguez-Franco[6],* Javier *Pizarro-Delgado[7,8,9], Rubén Corpas[10,11], Christian Griñán-Ferré[12], Mercè Pallàs[12], Coral Sanfeliu[10,11], Manuel Vázquez-Carrera[7,8,9], Bruce D. Hammock[4], Ferran Feixas[2], Santiago Vázquez[1*]*

[1]Laboratori de Química Farmacèutica (Unitat Associada al CSIC), Departament de Farmacologia, Toxicologia i Química Terapèutica, Facultat de Farmàcia i Ciències de l'Alimentació, and Institute of Biomedicine (IBUB), Universitat de Barcelona, Av. Joan XXIII, 27-31, 08028 Barcelona, Spain.

[2]CompBioLab Group, Departament de Química and Institut de Química Computacional i Catàlisi (IQCC), Universitat de Girona, C/ Maria Aurèlia Capmany 69, 17003 Girona, Spain.

[3]Institució Catalana de Recerca i Estudis Avançats (ICREA), 08010 Barcelona, Spain.

[4]Department of Entomology and Nematology and Comprehensive Cancer Center, University of California, Davis, CA 95616, USA.

[5]Drug Screening Platform/Biofarma Research Group, CIMUS Research Center. Departamento de Farmacoloxía, Farmacia e Tecnoloxía Farmacéutica. University of Santiago de Compostela (USC), 15782 Santiago de Compostela, Spain.

[6]Institute of Medicinal Chemistry, Spanish National Research Council (CSIC), C/Juan de la Cierva 3, 28006 Madrid, Spain.

[7]Pharmacology Section. Department of Pharmacology, Toxicology and Medicinal Chemistry, Faculty of Pharmacy and Food Sciences, University of Barcelona, and Institute of Biomedicine of the University of Barcelona (IBUB), Av. Joan XXIII, 27-31, 08028 Barcelona, Spain.

[8]Spanish Biomedical Research Center in Diabetes and Associated Metabolic Diseases (CIBERDEM)-Instituto de Salud Carlos III, 28029 Madrid, Spain.

[9]Pediatric Research Institute-Hospital Sant Joan de Déu, 08950 Esplugues de Llobregat, Spain.

[10]Institute of Biomedical Research of Barcelona (IIBB), CSIC and IDIBAPS, 08036 Barcelona, Spain.

[11]CIBER Epidemiology and Public Health (CIBERESP)-Instituto de Salud Carlos III, 28029, Madrid, Spain.

[12]Pharmacology Section. Department of Pharmacology, Toxicology and Medicinal Chemistry, Faculty of Pharmacy and Food Sciences, and Institut de Neurociències, University of Barcelona, Av. Joan XXIII, 27-31, 08028 Barcelona, Spain.

* Corresponding author. Tel.: +34 934024533; *E-mail address:* svazquez@ub.edu *(S. Vázquez).*

Table of contents

^{1}H and ^{13}C NMR spectra of compound **9**	Page S6
^{1}H and ^{13}C NMR spectra of compound **11**	Page S7
^{1}H and ^{13}C NMR spectra of compound **13**	Page S8
^{1}H and ^{13}C NMR spectra of compound **14**	Page S9
^{1}H and ^{13}C NMR spectra of compound **16**	Page S10
^{1}H and ^{13}C NMR spectra of compound **17**	Page S11
^{1}H and ^{13}C NMR spectra of compound **18**	Page S12
^{1}H and ^{13}C NMR spectra of compound **20**	Page S13
^{1}H and ^{13}C NMR spectra of compound **21**	Page S14
^{1}H and ^{13}C NMR spectra of compound **22**	Page S15
^{1}H and ^{13}C NMR spectra of compound **23**	Page S16
Table S1: Elemental analysis data	Page S17
HPLC trace for compound **22**	Page S18
Figure S1: Active site volume fluctuations	Page S19
Figure S2: Root-mean-square fluctuation of the C-terminal region of sEH	Page S20
Figure S3: Non-covalent interactions between the inhibitors and the active site residues of sEH	Page S21
Figure S4: Analysis of active site water molecules in MD simulations	Page S22
Figure S5: Water occupation in the sEH active site and water distribution for compounds **20**, **22** and **23**	Page S23
Figure S6: Rotation of the benzohomoadamantane and adamantane moieties in the pocket of the sEH active site	Page S24
Figure S7: Apo state aMD simulations with compounds *t*-AUCB, **20**, **22** and **23**	Page S25
Table S2: Mean of concentrations of **20** in mouse plasma	Page S26
Table S3: Mean of concentrations of **22** in mouse plasma	Page S27
Figure S8: Concentrations of **20** in mouse plasma after ip administration	Page S28

Figure S9: Concentrations of **22** in mouse plasma after ip administration Page S28

Table S4: Histologic scoring of pancreatic tissues of control group Page S29

Table S5: Histologic scoring of pancreatic tissues of mice treated with cerulein Page S29

Table S6: Histologic scoring of pancreatic tissues of mice treated with cerulein and **22** at 0.1 mg/kg Page S29

Table S7: Histologic scoring of pancreatic tissues of mice treated with cerulein and **22** at 0.3 mg/kg Page S29

References Page S30

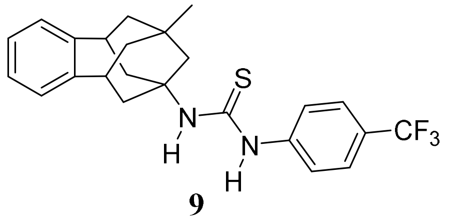
S
N
H
N
H
CF3

9

,

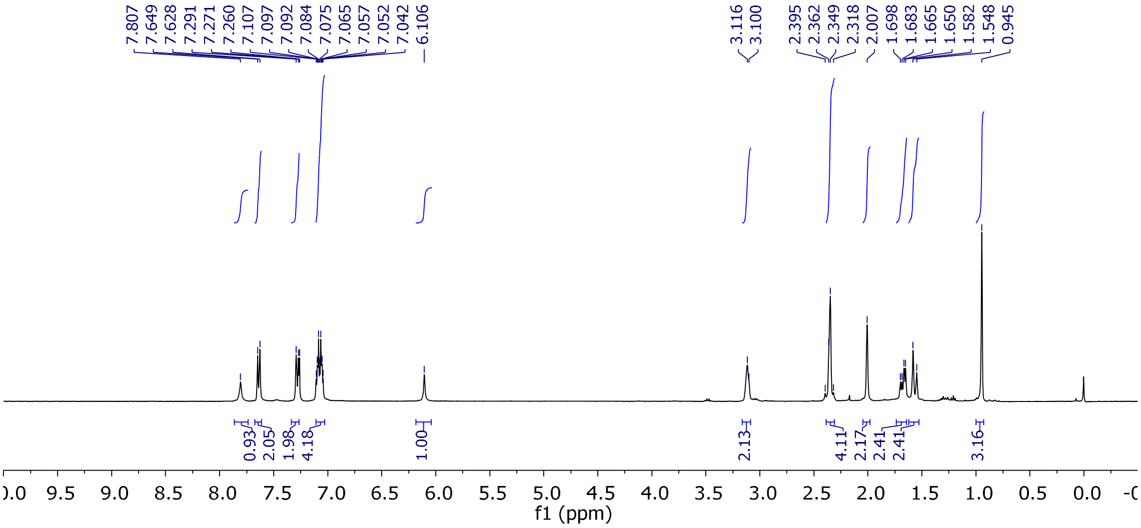
7.807
7.649
7.628
7.291
7.271
7.260
7.107
7.097
7.092
7.084
7.075
7.065
7.057
7.052
7.042
6.106
3.116
3.100
2.395
2.362
2.349
2.318
2.007
1.698
1.683
1.665
1.650
1.582
1.548
0.945
0.93
2.05
1.98
4.18
1.00
2.13
4.11
2.17
2.41
2.41
3.16
f1 (ppm)

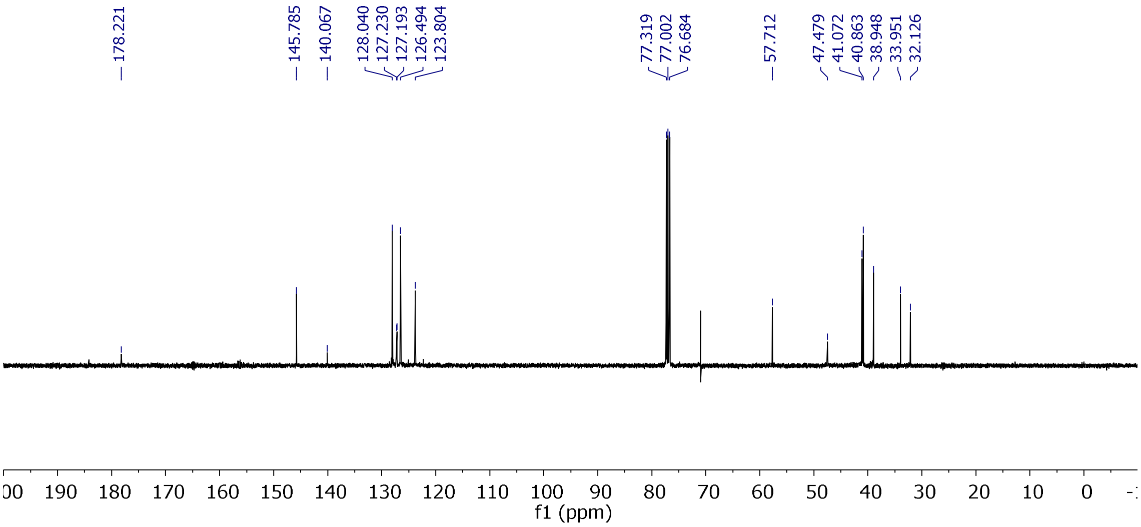
178.221
145.785
140.067
128.040
127.230
127.193
126.494
123.804
77.319
77.002
76.684
57.712
47.479
41.072
40.863
38.948
33.951
32.126
f1 (ppm)

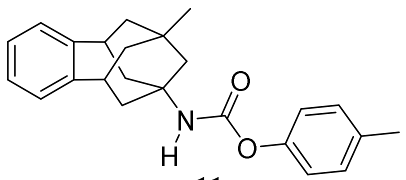

11

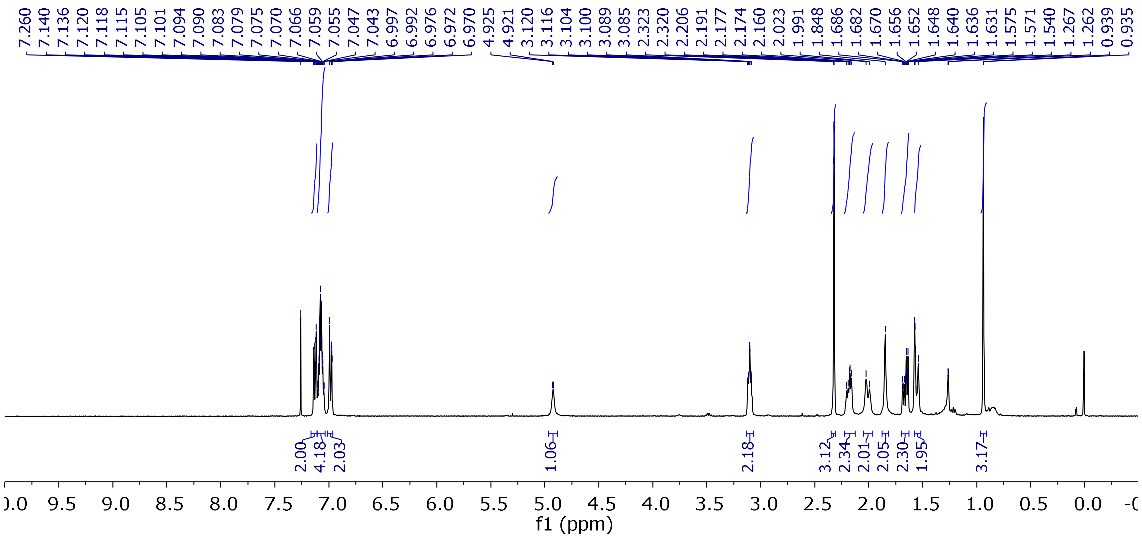

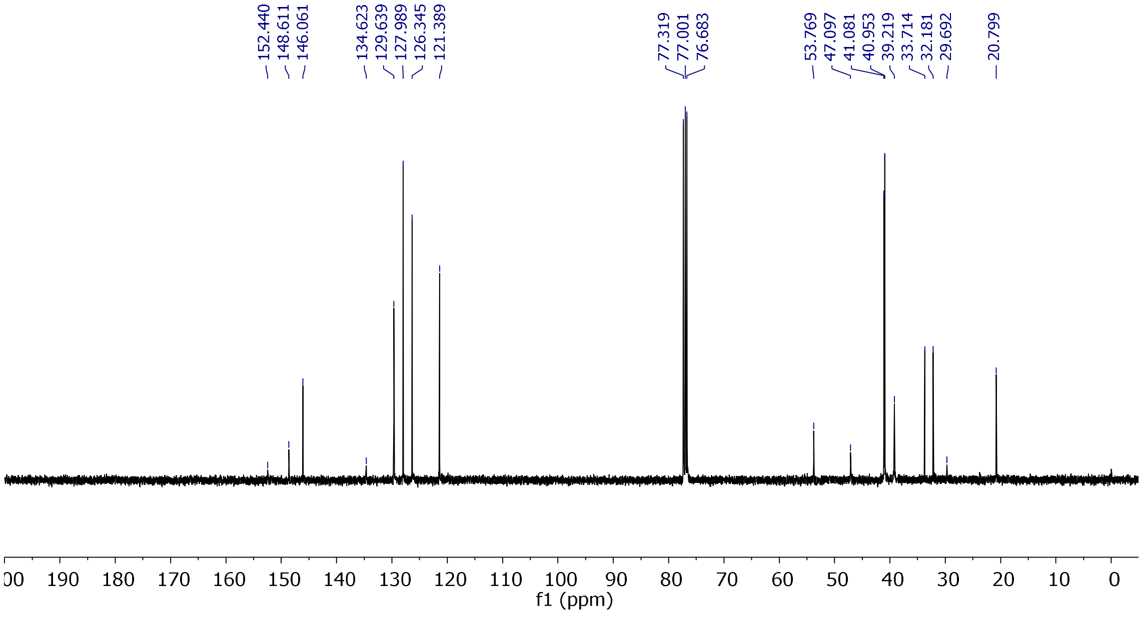

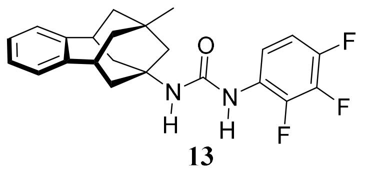
F
O
F
N
N
H
H
F

13

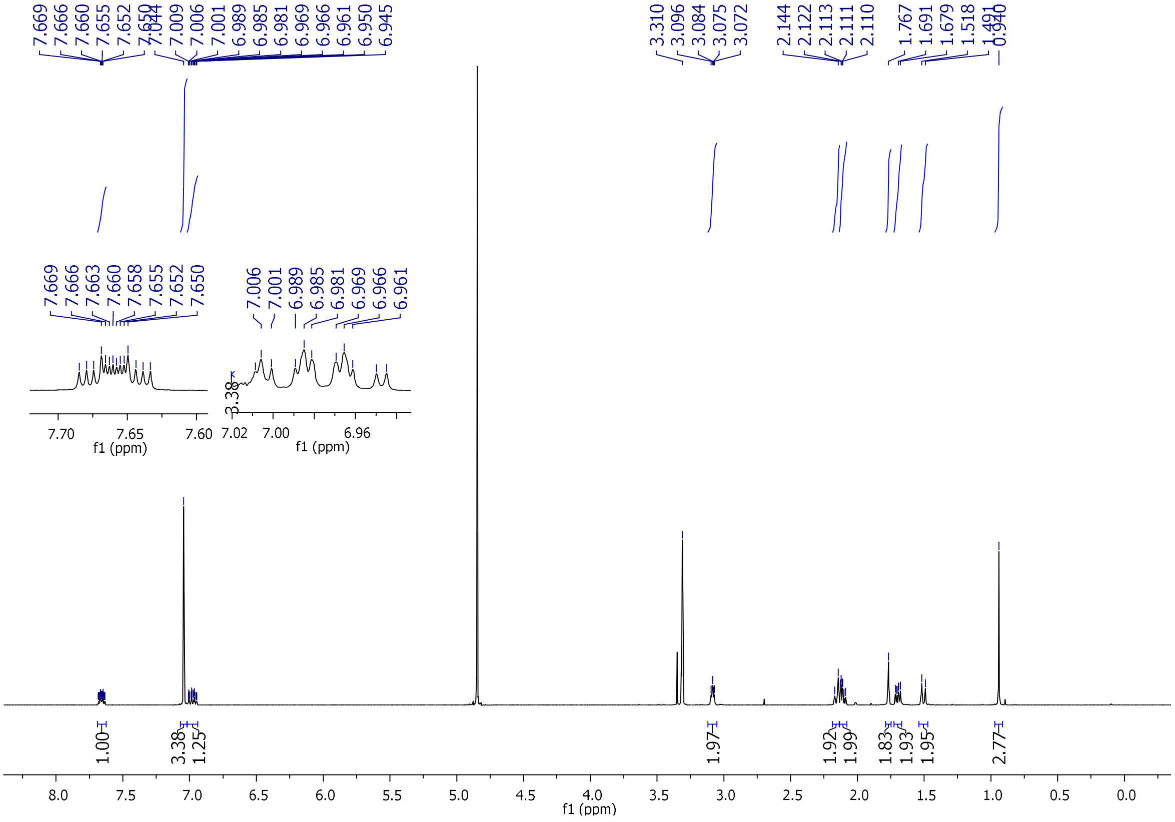
7.669
7.666
7.660
7.655
7.652
7.650
7.644
7.009
7.006
7.001
6.989
6.985
6.981
6.969
6.966
6.961
6.950
6.945
3.310
3.096
3.084
3.075
3.072
2.144
2.122
2.113
2.111
2.110
1.767
1.691
1.679
1.518
1.491
0.940
7.669
7.666
7.663
7.660
7.658
7.655
7.652
7.650
7.70
7.65
7.60
f1 (ppm)
7.006
7.001
6.989
6.985
6.981
6.969
6.966
6.961
3.38
7.02
7.00
6.96
f1 (ppm)
1.00
3.38
1.25
1.97
1.92
1.99
1.83
1.93
1.95
2.77
8.0
7.5
7.0
6.5
6.0
5.5
5.0
4.5
4.0
3.5
3.0
2.5
2.0
1.5
1.0
0.5
0.0
f1 (ppm)

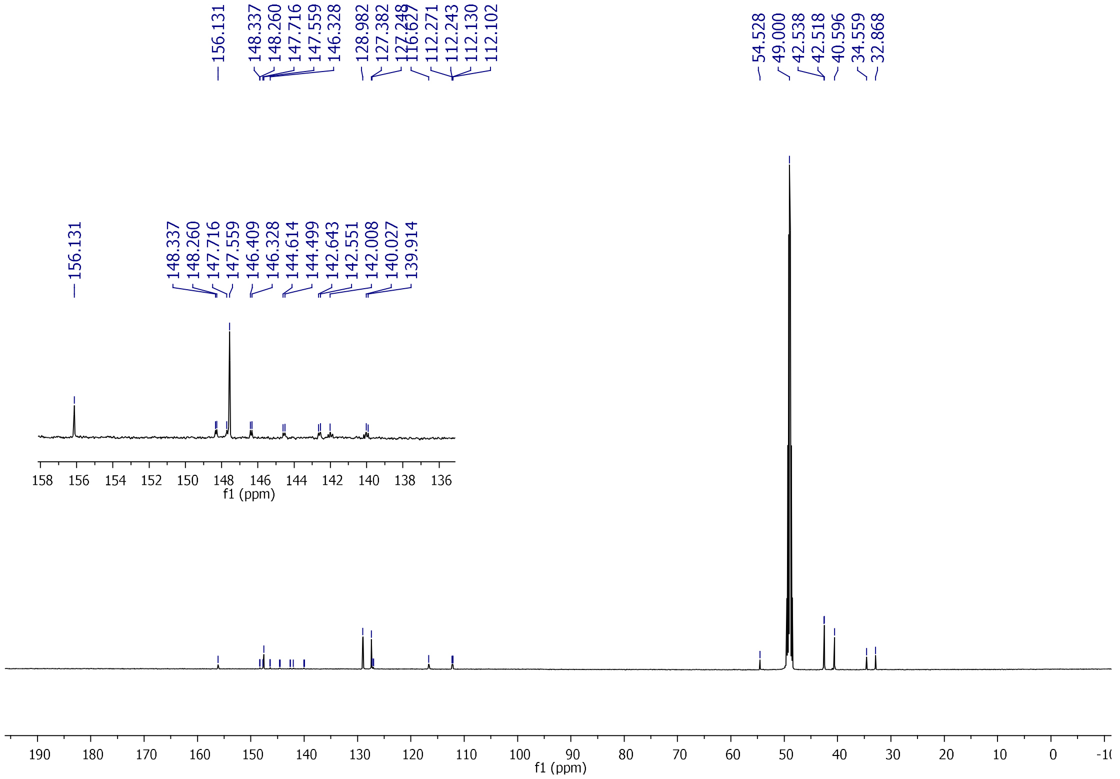
156.131
148.337
148.260
147.716
147.559
146.328
128.982
127.382
127.248
116.627
112.271
112.243
112.130
112.102
54.528
49.000
42.538
42.518
40.596
34.559
32.868
156.131
148.337
148.260
147.716
147.559
146.409
146.328
144.614
144.499
142.643
142.551
142.008
140.027
139.914
158
156
154
152
150
148
146
144
142
140
138
136
f1 (ppm)
190
180
170
160
150
140
130
120
110
100
90
80
70
60
50
40
30
20
10
0
f1 (ppm)

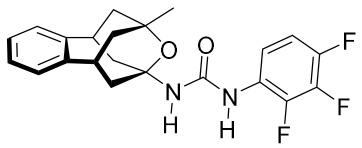

14

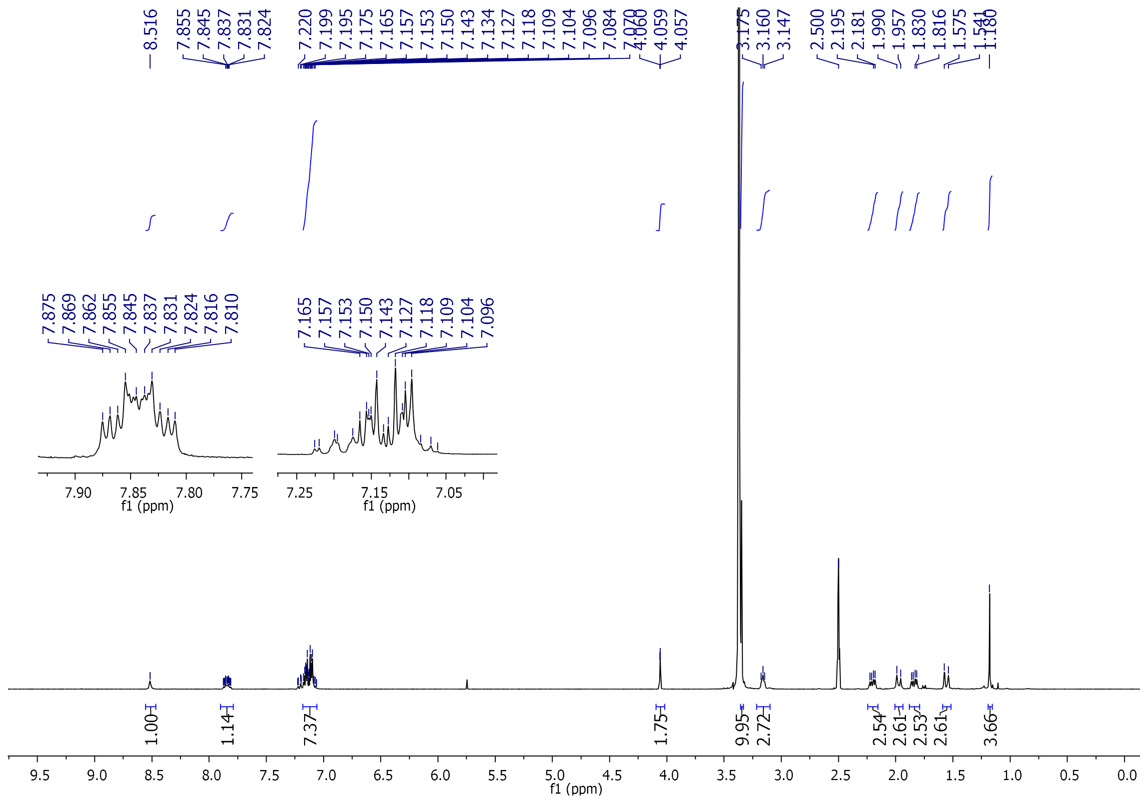

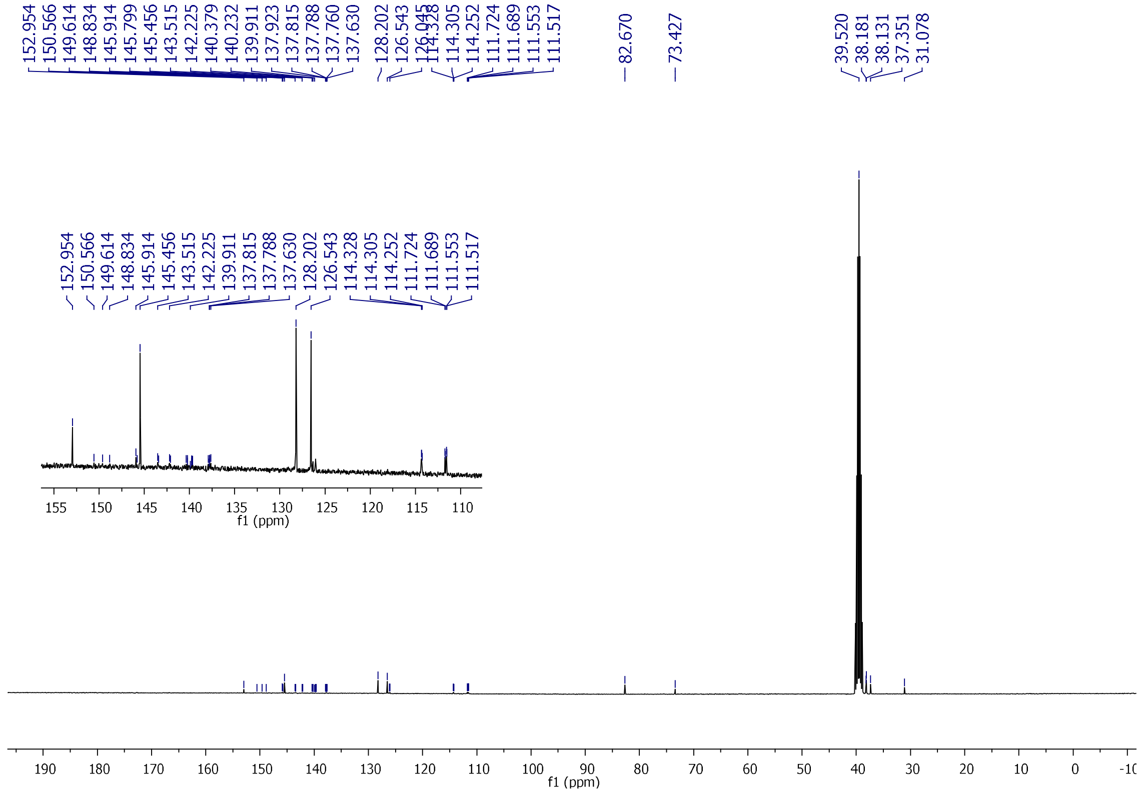

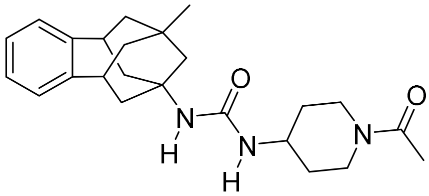

16

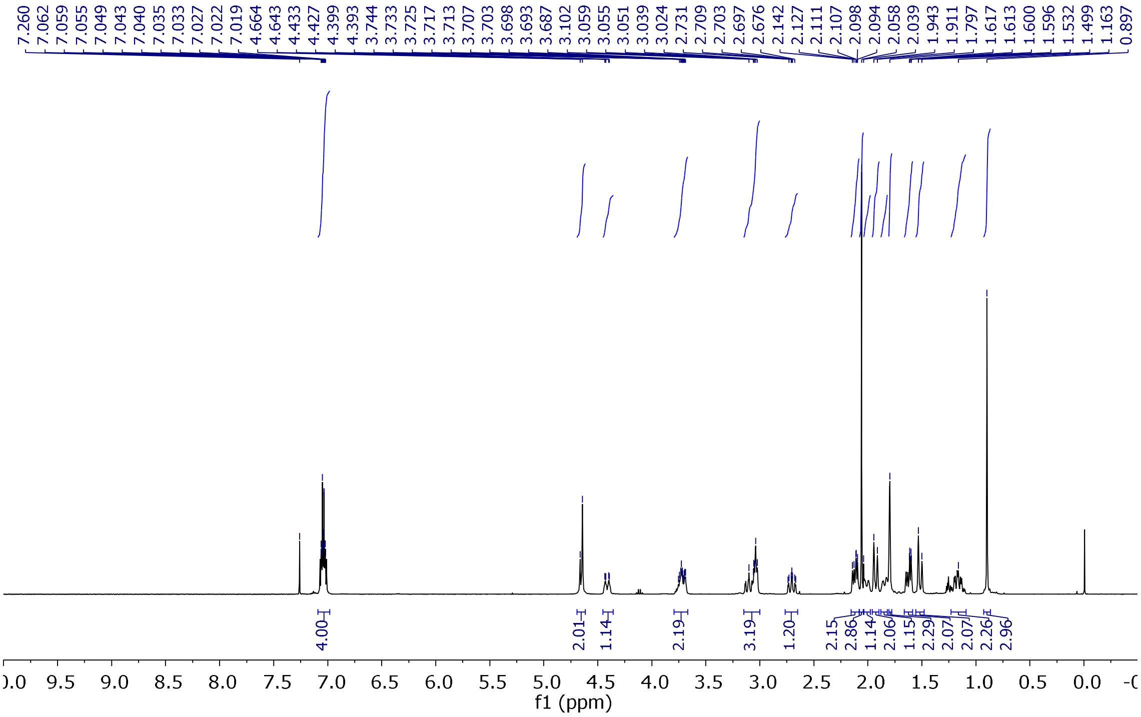
7.260 7.062 7.059 7.055 7.049 7.043 7.040 7.035 7.033 7.027 7.022 7.019 4.664 4.643 4.433 4.427 4.399 4.393 3.744 3.733 3.725 3.717 3.713 3.707 3.703 3.698 3.693 3.687 3.102 3.059 3.055 3.051 3.039 3.024 2.731 2.709 2.703 2.697 2.676 2.142 2.127 2.111 2.107 2.098 2.094 2.058 2.039 1.943 1.911 1.797 1.617 1.613 1.600 1.596 1.532 1.499 1.163 0.897
4.00 2.01 1.14 2.19 3.19 1.20 2.15 2.86 1.14 2.06 1.15 2.29 2.07 2.07 2.26 2.96
f1 (ppm)

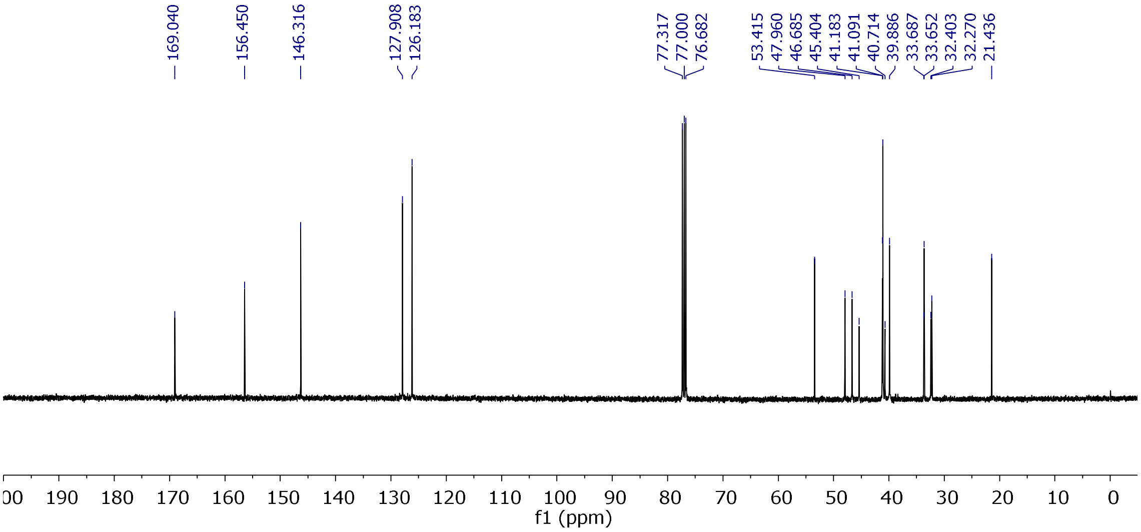
169.040 156.450 146.316 127.908 126.183 77.317 77.000 76.682 53.415 47.960 46.685 45.404 41.183 41.091 40.714 39.886 33.687 33.652 32.403 32.270 21.436
f1 (ppm)

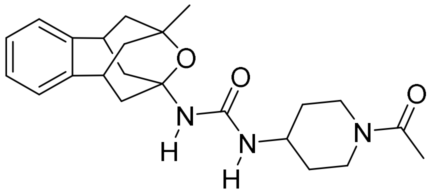

17

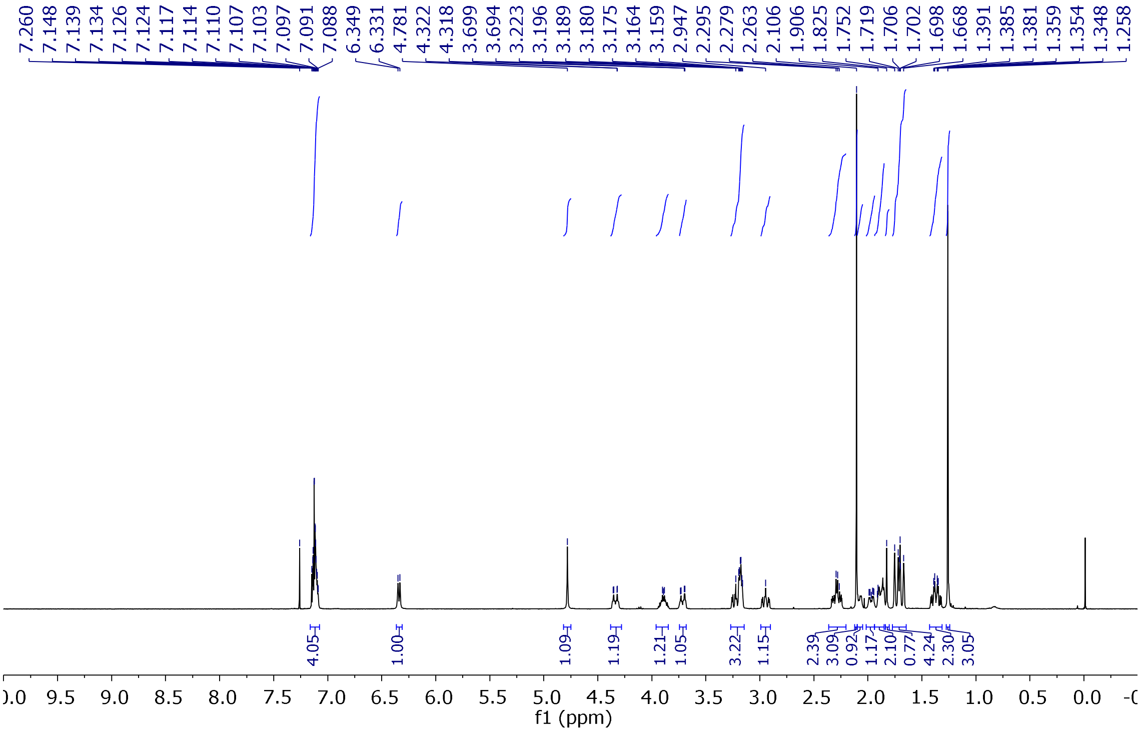

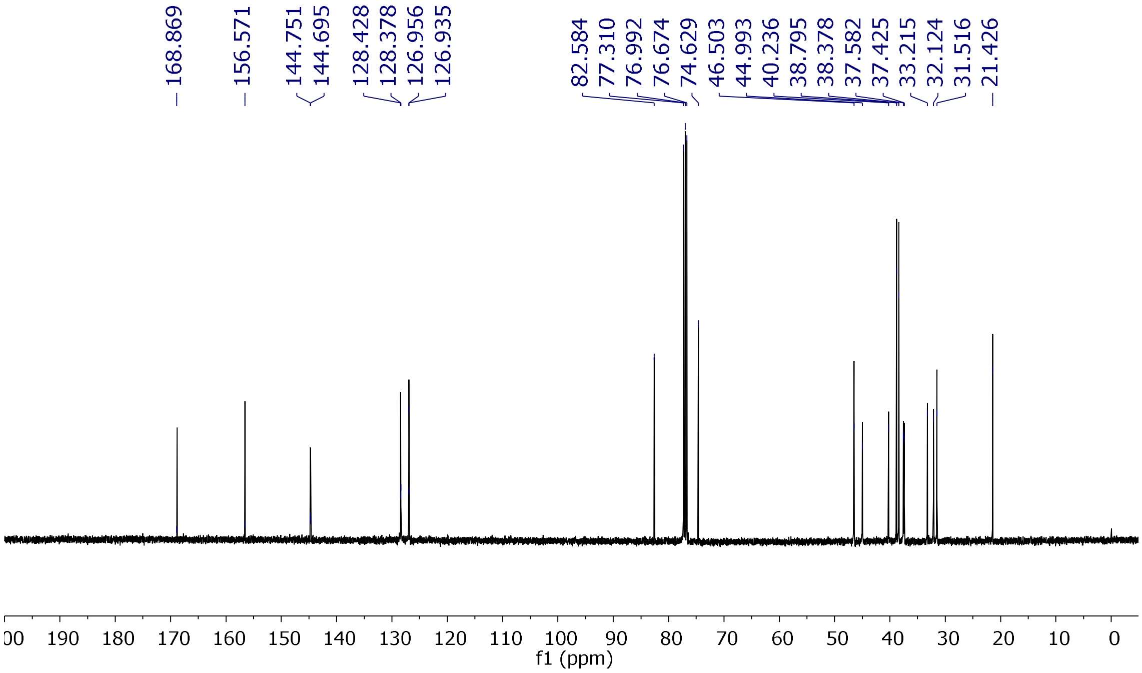

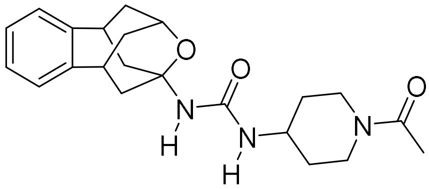

18

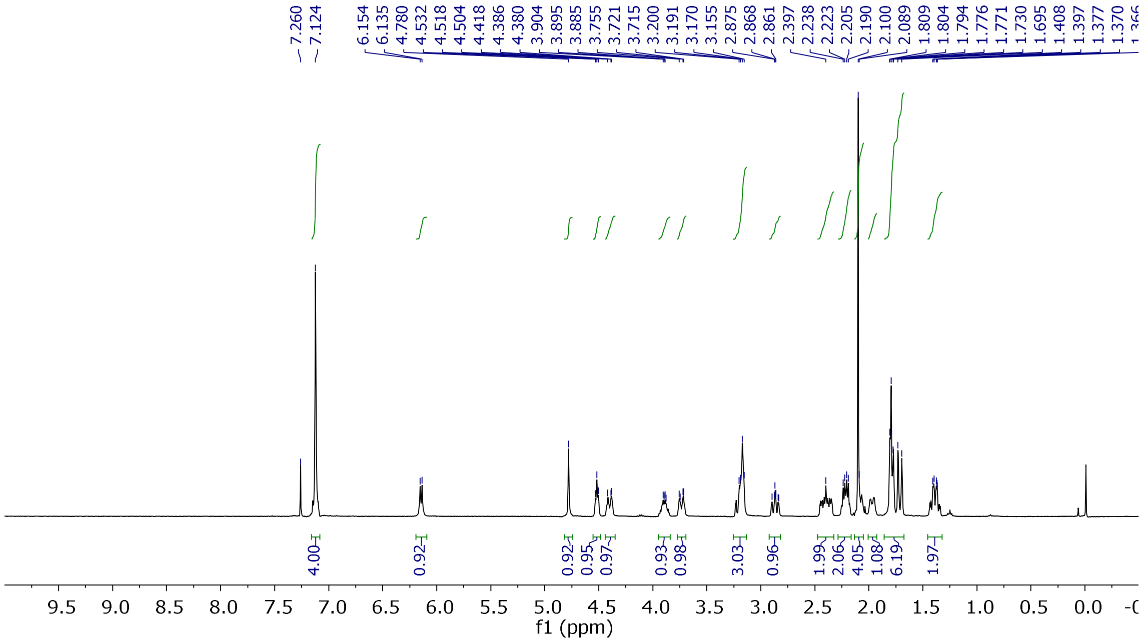
7.260
7.124
6.154
6.135
4.780
4.532
4.518
4.504
4.418
4.386
4.380
3.904
3.895
3.885
3.755
3.721
3.715
3.200
3.191
3.170
3.155
2.875
2.868
2.861
2.397
2.238
2.223
2.205
2.190
2.100
2.089
1.809
1.804
1.794
1.776
1.771
1.730
1.695
1.408
1.397
1.377
1.370
4.00
0.92
0.92
0.95
0.97
0.93
0.98
3.03
0.96
1.99
2.06
4.05
1.08
6.19
1.97
9.5
9.0
8.5
8.0
7.5
7.0
6.5
6.0
5.5
5.0
4.5
4.0
3.5
3.0
2.5
2.0
1.5
1.0
0.5
0.0
f1 (ppm)

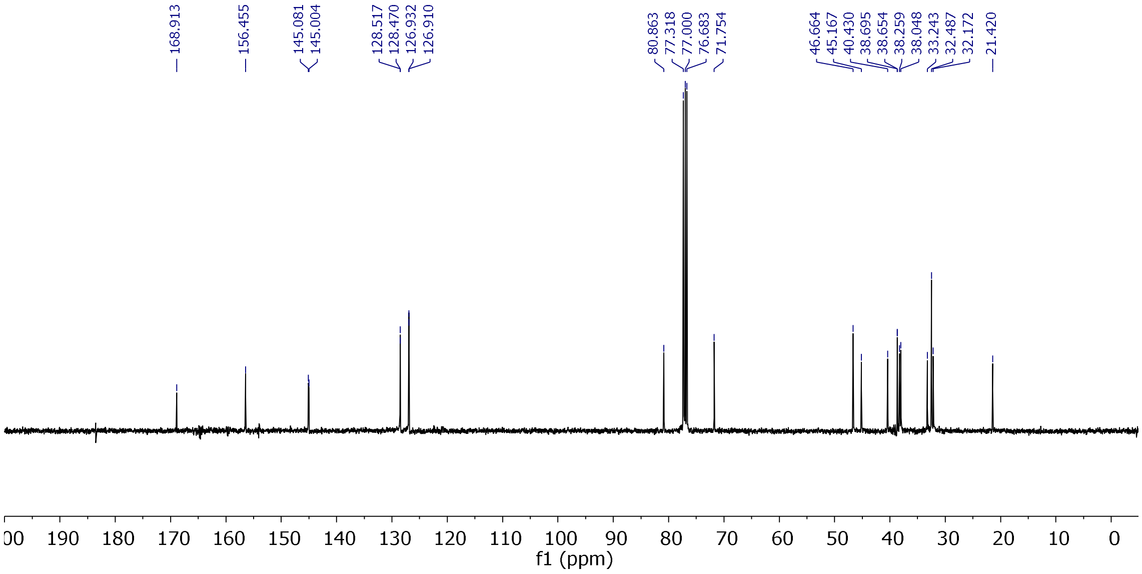
168.913
156.455
145.081
145.004
128.517
128.470
126.932
126.910
80.863
77.318
77.000
76.683
71.754
46.664
45.167
40.430
38.695
38.654
38.259
38.048
33.243
32.487
32.172
21.420
190
180
170
160
150
140
130
120
110
100
90
80
70
60
50
40
30
20
10
0
f1 (ppm)

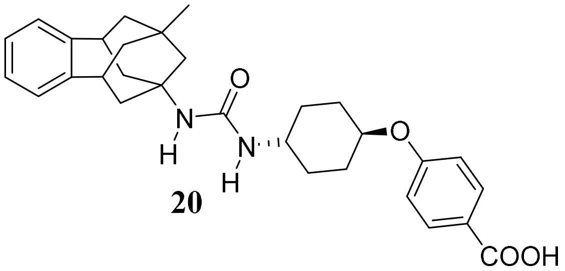
O
N
H
N
H
O
COOH

20

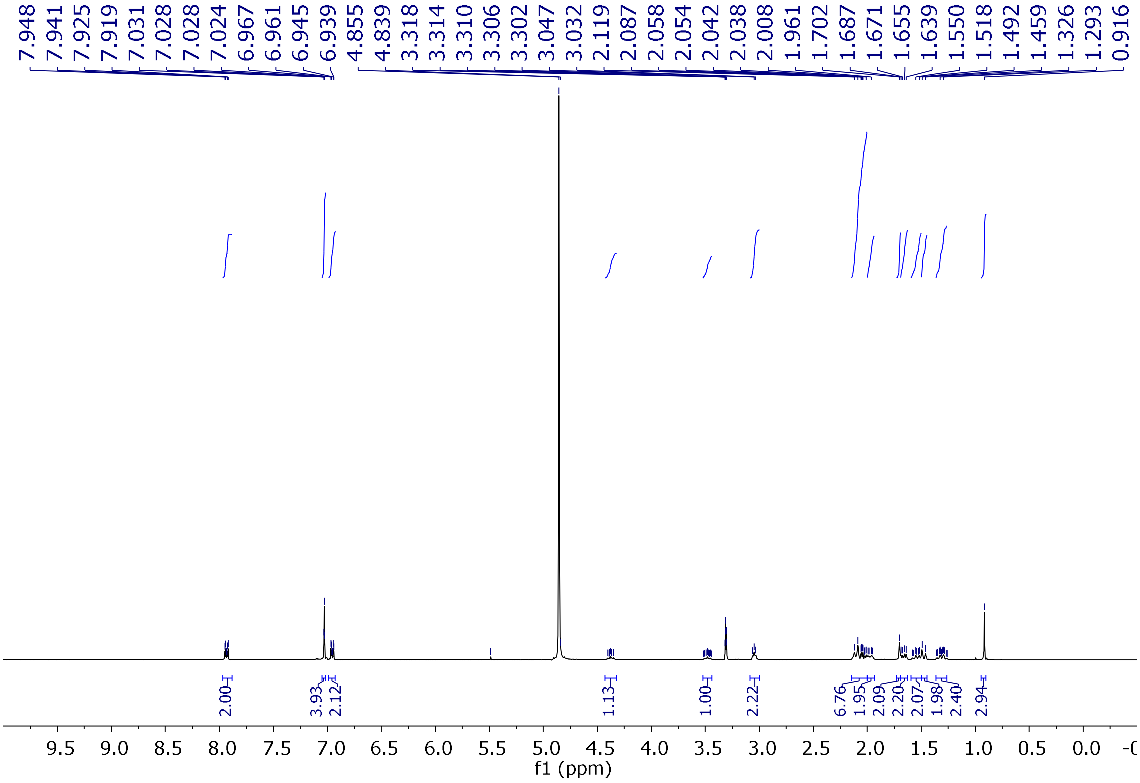
7.948 7.941 7.925 7.919 7.031 7.028 7.028 7.024 6.967 6.961 6.945 6.939 4.855 4.839 3.318 3.314 3.310 3.306 3.302 3.047 3.032 2.119 2.087 2.058 2.054 2.042 2.038 2.008 1.961 1.702 1.687 1.671 1.655 1.639 1.550 1.518 1.492 1.459 1.326 1.293 0.916
2.00 3.93 2.12 1.13 1.00 2.22 6.76 1.95 2.09 2.20 2.07 1.98 2.40 2.94
9.5 9.0 8.5 8.0 7.5 7.0 6.5 6.0 5.5 5.0 4.5 4.0 3.5 3.0 2.5 2.0 1.5 1.0 0.5 0.0
f1 (ppm)

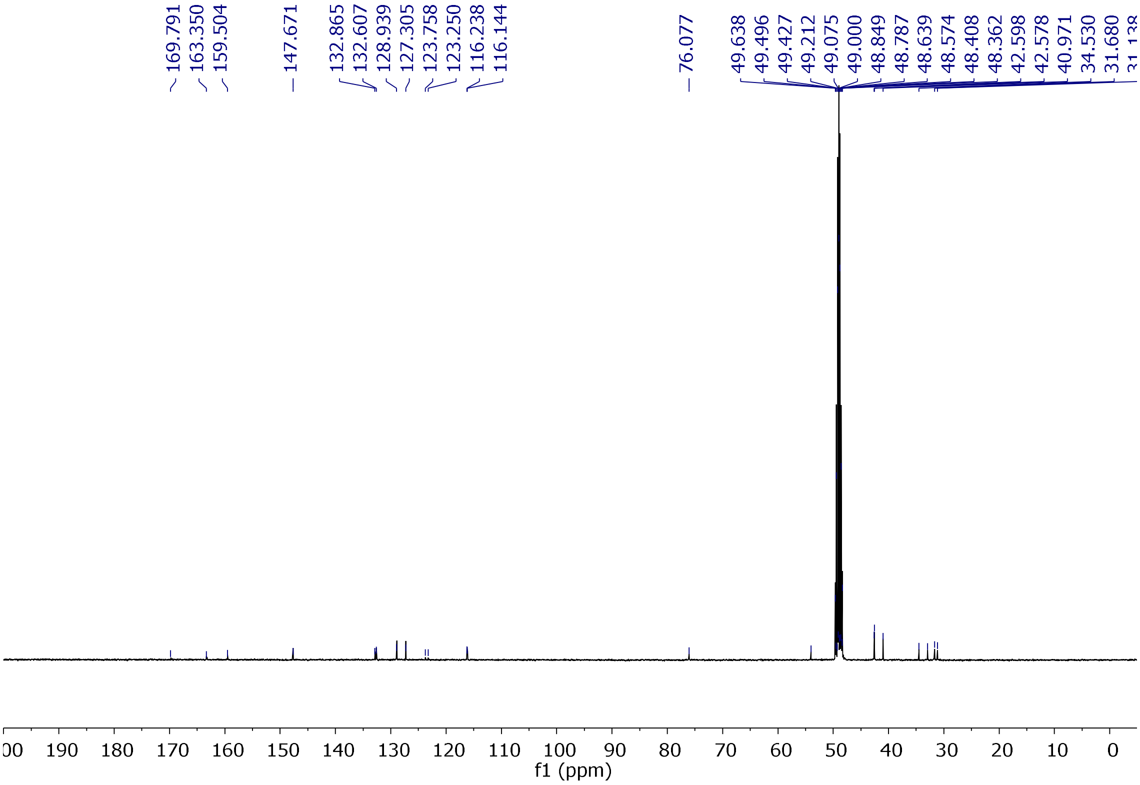
169.791 163.350 159.504 147.671 132.865 132.607 128.939 127.305 123.758 123.250 116.238 116.144 76.077 49.638 49.496 49.427 49.212 49.075 49.000 48.849 48.787 48.639 48.574 48.408 48.362 42.598 42.578 40.971 34.530 31.680
190 180 170 160 150 140 130 120 110 100 90 80 70 60 50 40 30 20 10 0
f1 (ppm)

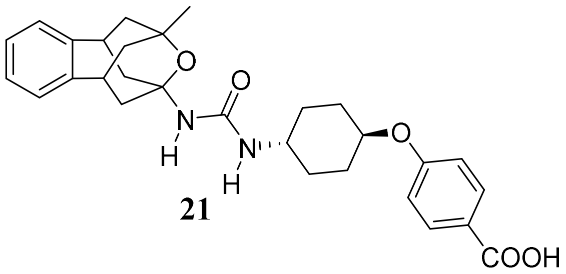
O
N
H
N
H
O
O
COOH

21

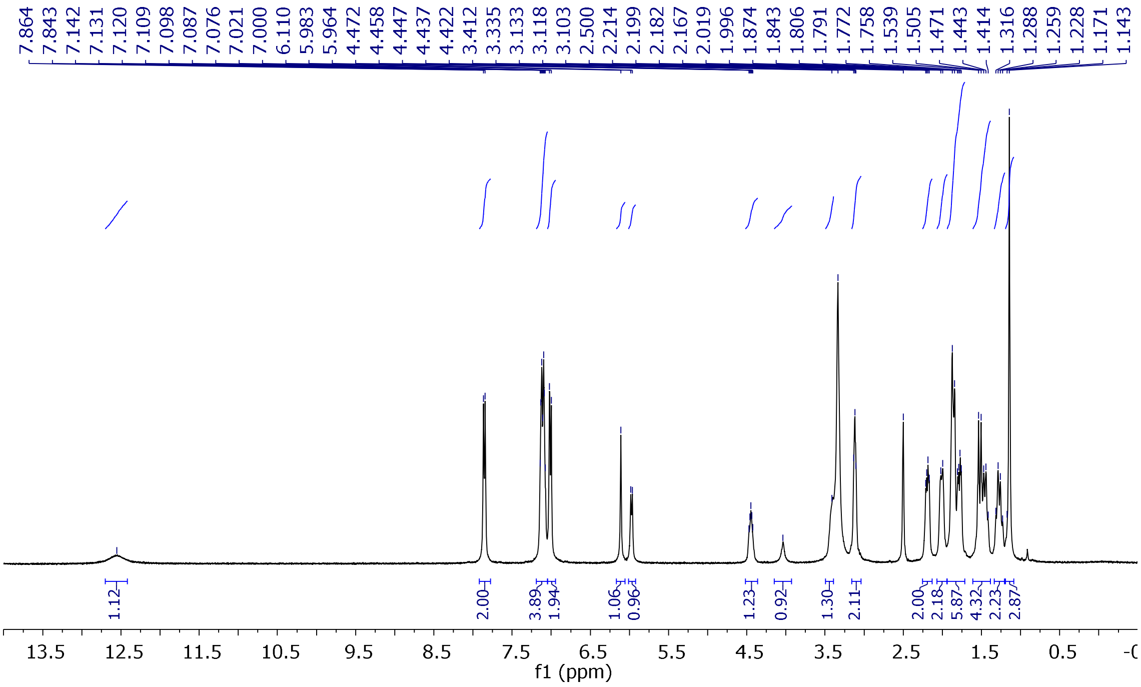
7.864
7.843
7.142
7.131
7.120
7.109
7.098
7.087
7.076
7.021
7.000
6.110
5.983
5.964
4.472
4.458
4.447
4.437
4.422
3.412
3.335
3.133
3.118
3.103
2.500
2.214
2.199
2.182
2.167
2.019
1.996
1.874
1.843
1.806
1.791
1.772
1.758
1.539
1.505
1.471
1.443
1.414
1.316
1.288
1.259
1.228
1.171
1.143
1.12
2.00
3.89
1.94
1.06
0.96
1.23
0.92
1.30
2.11
2.00
2.18
5.87
4.32
2.23
2.87
13.5
12.5
11.5
10.5
9.5
8.5
7.5
6.5
5.5
4.5
3.5
2.5
1.5
0.5
f1 (ppm)

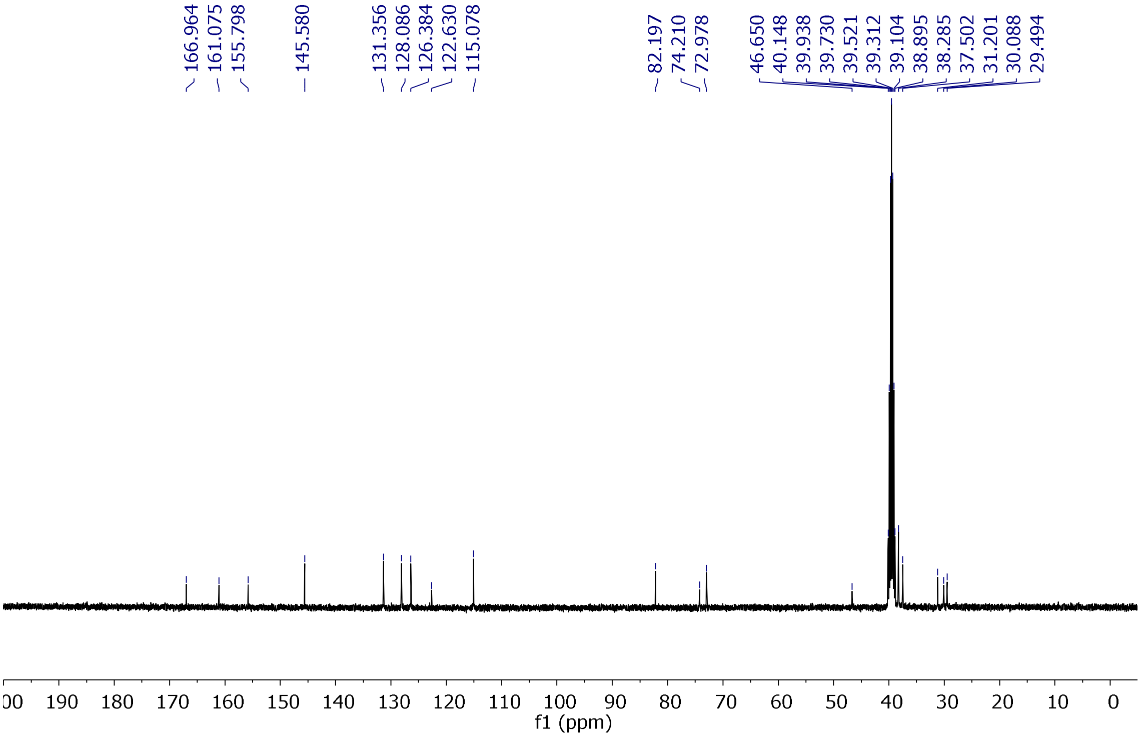
166.964
161.075
155.798
145.580
131.356
128.086
126.384
122.630
115.078
82.197
74.210
72.978
46.650
40.148
39.938
39.730
39.521
39.312
39.104
38.895
38.285
37.502
31.201
30.088
29.494
190
180
170
160
150
140
130
120
110
100
90
80
70
60
50
40
30
20
10
0
f1 (ppm)

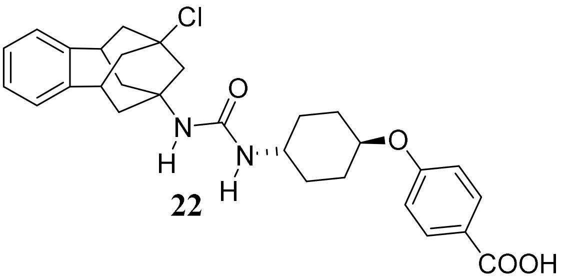

22

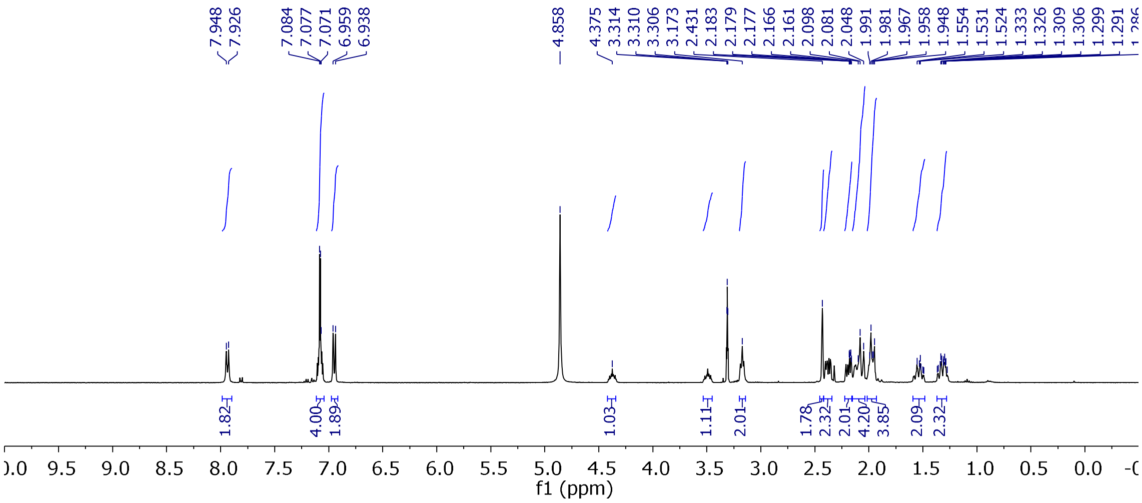
7.948
7.926
7.084
7.077
7.071
6.959
6.938
4.858
4.375
3.314
3.310
3.306
3.173
2.431
2.183
2.179
2.177
2.166
2.161
2.098
2.081
2.048
1.991
1.981
1.967
1.958
1.948
1.554
1.531
1.524
1.333
1.326
1.309
1.306
1.299
1.291
[illegible]
1.82
4.00
1.89
1.03
1.11
2.01
1.78
2.32
2.01
4.20
3.85
2.09
2.32
f1 (ppm)

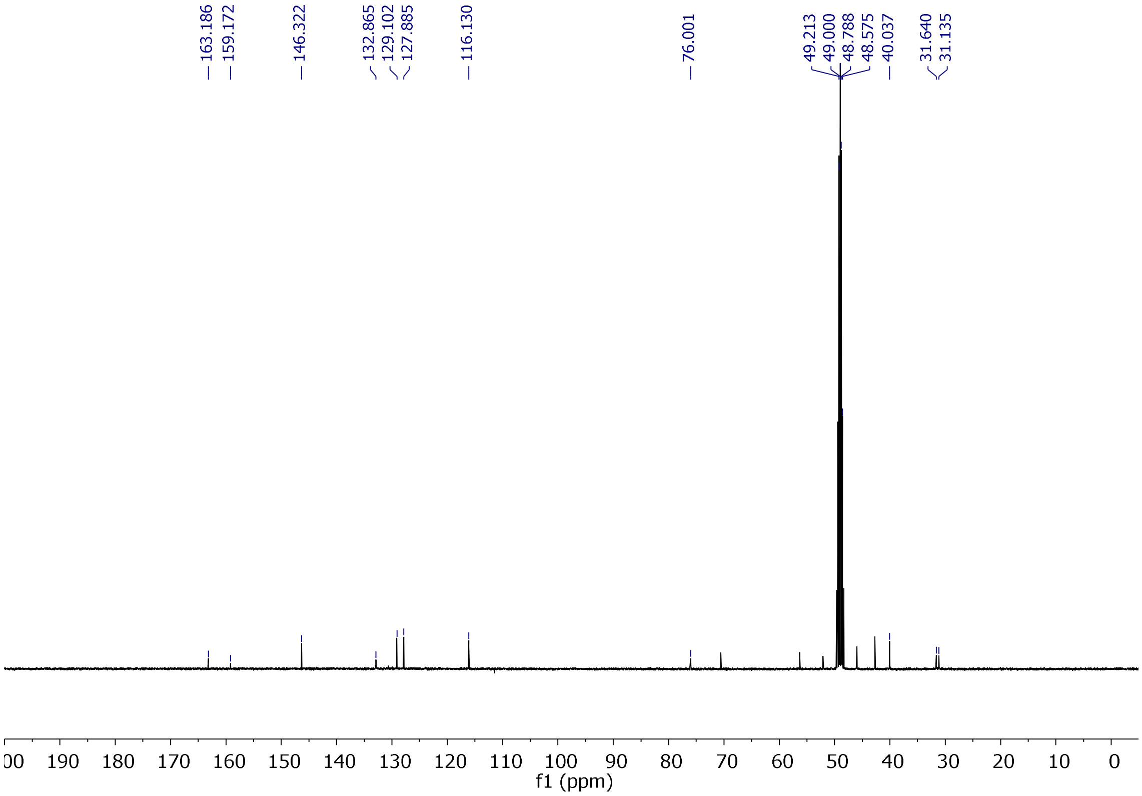
163.186
159.172
146.322
132.865
129.102
127.885
116.130
76.001
49.213
49.000
48.788
48.575
40.037
31.640
31.135
f1 (ppm)

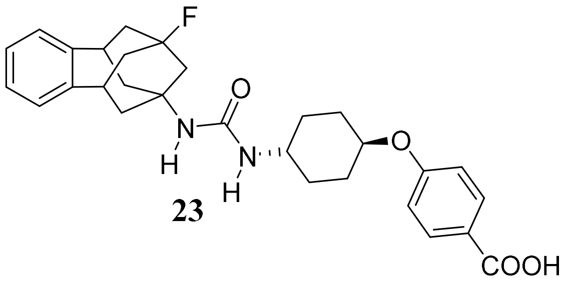
F
O
N
H
COOH

23

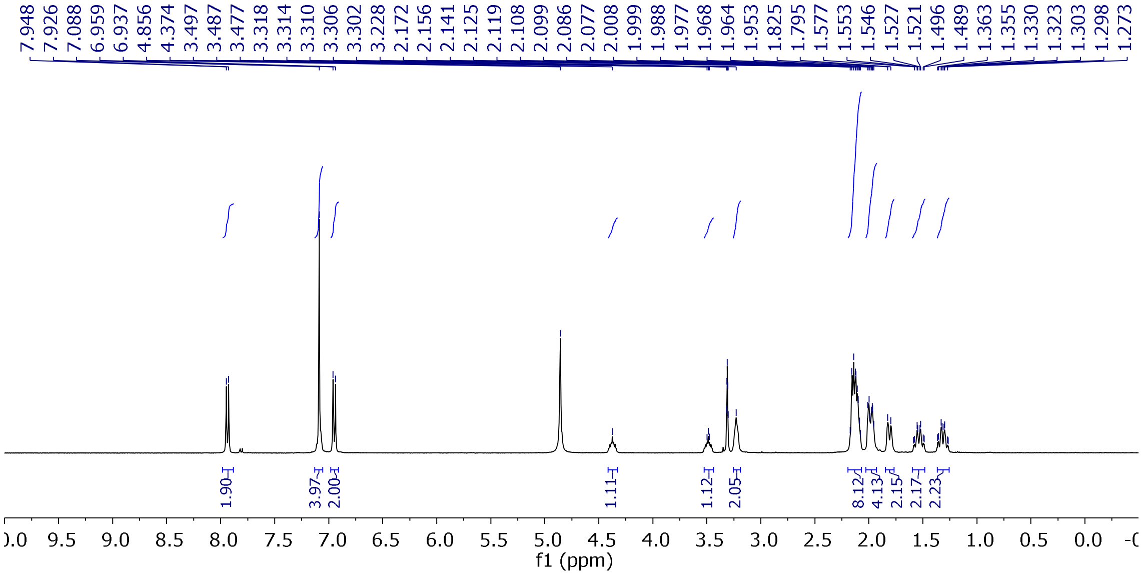
7.948
7.926
7.088
6.959
6.937
4.856
4.374
3.497
3.487
3.477
3.318
3.314
3.310
3.306
3.302
3.228
2.172
2.156
2.141
2.125
2.119
2.108
2.099
2.086
2.077
2.008
1.999
1.988
1.977
1.968
1.964
1.953
1.825
1.795
1.577
1.553
1.546
1.527
1.521
1.496
1.489
1.363
1.355
1.330
1.323
1.303
1.298
1.273
1.90
3.97
2.00
1.11
1.12
2.05
8.12
4.13
2.15
2.17
2.23
9.5 9.0 8.5 8.0 7.5 7.0 6.5 6.0 5.5 5.0 4.5 4.0 3.5 3.0 2.5 2.0 1.5 1.0 0.5 0.0
f1 (ppm)

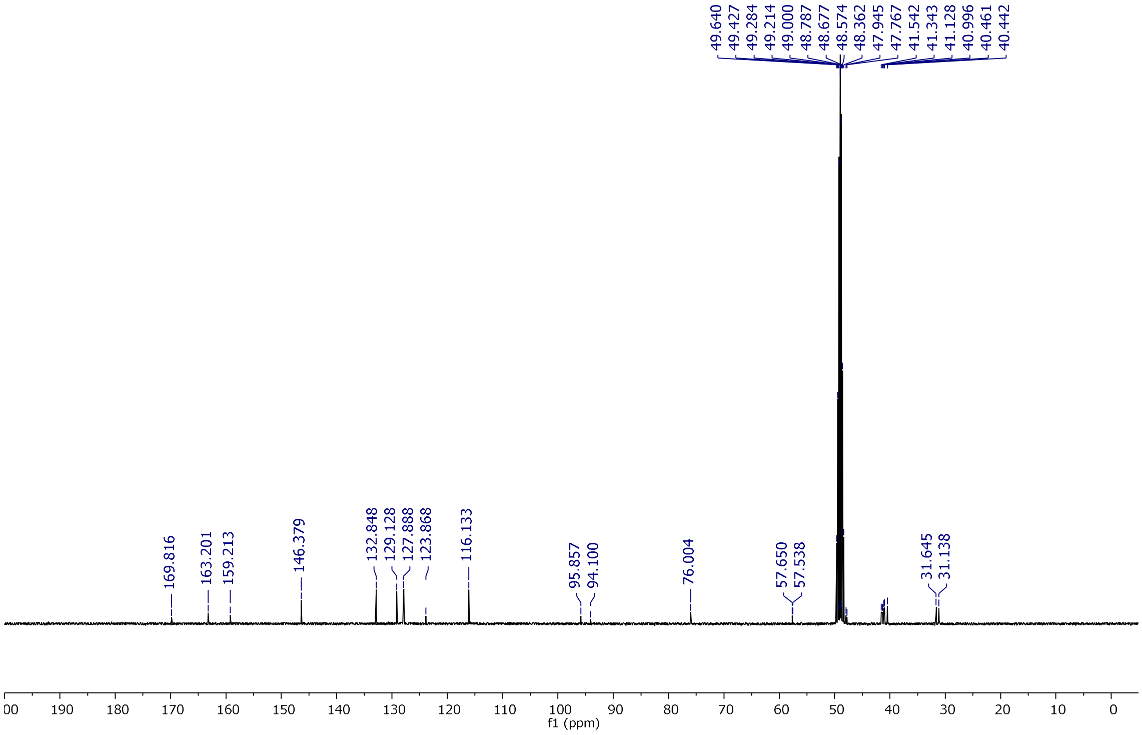
49.640
49.427
49.284
49.214
49.000
48.787
48.677
48.574
48.362
47.945
47.767
41.542
41.343
41.128
40.996
40.461
40.442
169.816
163.201
159.213
146.379
132.848
129.128
127.888
123.868
116.133
95.857
94.100
76.004
57.650
57.538
31.645
31.138
190 180 170 160 150 140 130 120 110 100 90 80 70 60 50 40 30 20 10 0
f1 (ppm)

Compound	Molecular Formula	Calculated			Found		
		C	H	N	C	H	N
**9**	$C_{24}H_{25}F_3N_2S$	66.96	5.85	6.51	66.79	5.95	6.37
**11**	$C_{24}H_{27}NO_2 \cdot 0.15\ H_2O$	79.15	7.56	3.85	79.34	7.92	3.48
**13**	$C_{23}H_{23}F_3N_2O$	68.99	5.79	7.00	68.94	5.92	6.71
**14**	$C_{22}H_{21}F_3N_2O_2 \cdot 0.1\ H_2O$	65.37	5.29	6.93	65.18	5.31	6.73
**16**	$C_{24}H_{33}N_3O_2 \cdot 0.25$ EtOAc	71.91	8.45	10.06	71.73	8.43	10.27
**17**	$C_{23}H_{31}N_3O_3$	69.49	7.86	10.57	69.47	7.92	10.38
**18**	$C_{22}H_{29}N_3O_3 \cdot 0.95\ H_2O$	65.96	7.77	10.49	66.25	7.67	10.13
**20**	$C_{30}H_{36}N_2O_4 \cdot 1.5\ H_2O$	69.88	7.62	5.43	69.53	7.37	5.10
**21**	$C_{29}H_{34}N_2O_5 \cdot 0.5\ CH_2Cl_2$	66.06	6.58	5.21	66.20	6.43	5.17
**22**	$C_{29}H_{33}ClN_2O_4 \cdot 1\ CH_3OH$	66.59	6.89	5.18	66.85	6.62	4.91
**23**	$C_{29}H_{33}FN_2O_4 \cdot 0.45\ CH_3OH$	69.77	6.92	5.53	69.68	6.79	5.57

Table S1: Elemental analysis data.

HPLC trace for compound **22**

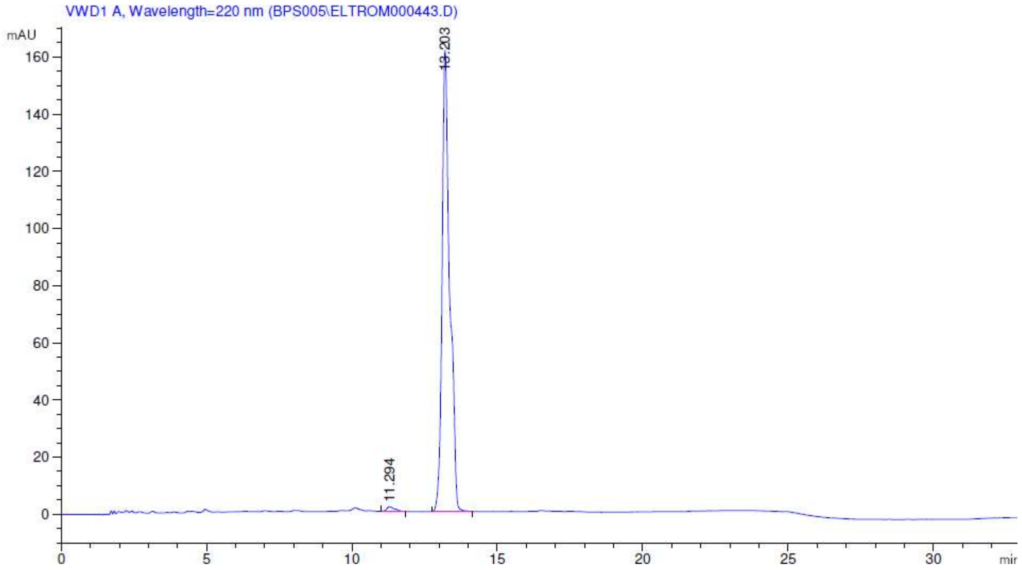

Signal 1: VWD1 A, Wavelength=220 nm

Peak #	RetTime [min]	Type	Width [min]	Area mAU *s	Height [mAU]	Area %
1	11.294	BB	0.2786	33.87679	1.70337	1.1300
2	13.203	BB	0.2636	2964.20068	161.49048	98.8700
Totals :				2998.07747	163.19384	

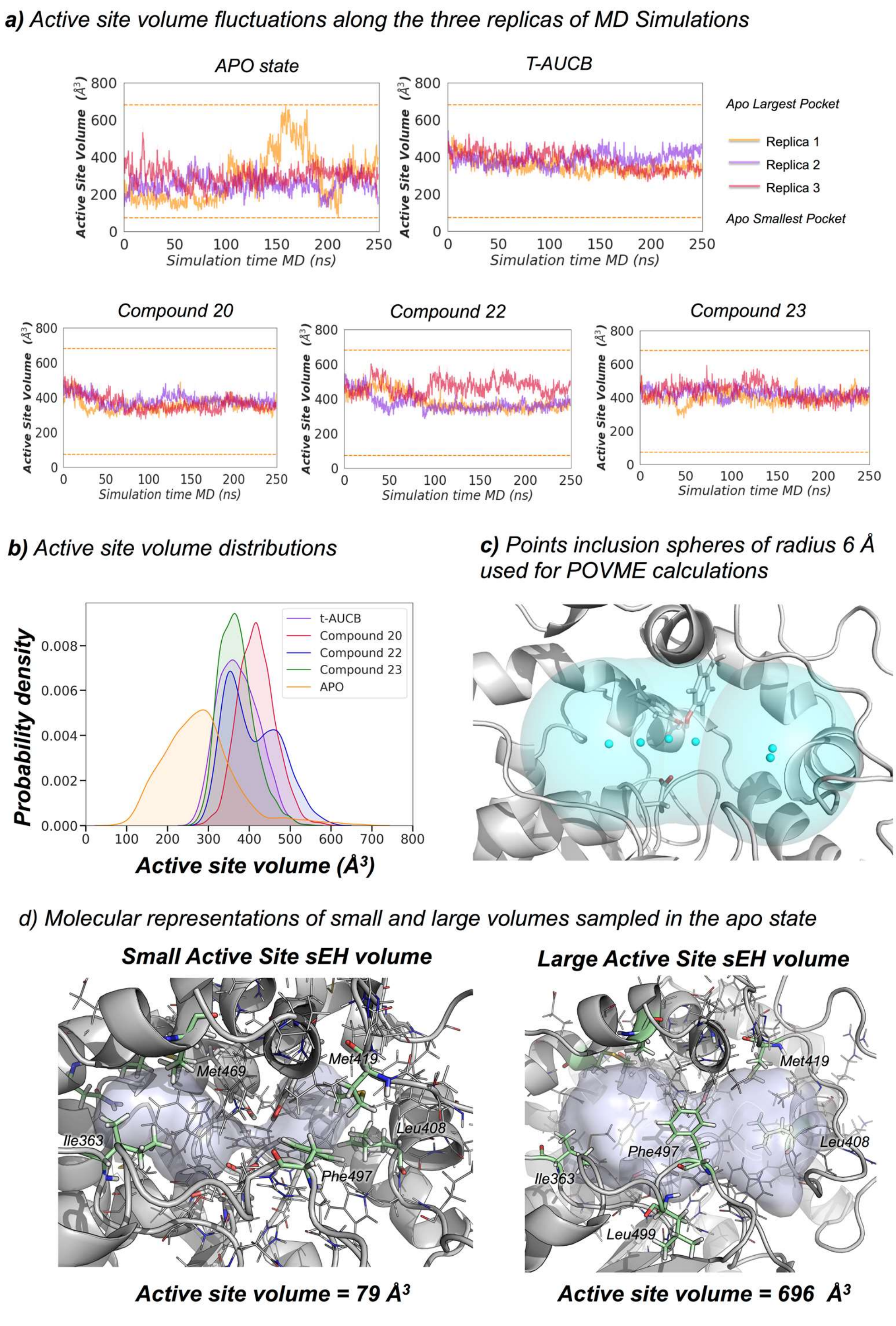

Figure S1: a) Representation of the active site volume fluctuations in the different MD replicas and systems considered calculated using the using the POcket Volume Measurer.[1] b) Probability density of the active site volumes for the apo state simulations (orange), and inhibitor bound simulations: *t*-AUCB (purple), **20** (red), **22** (blue), and **23** (green). c) Visualization of the 6 Å inclusion spheres used for POVME active site volume calculations. d) Representative MD snapshots displaying either small (left panel) or large active site sEH volumes (right panel) are shown. The most important residues affecting the volume are shown in sticks and labelled.

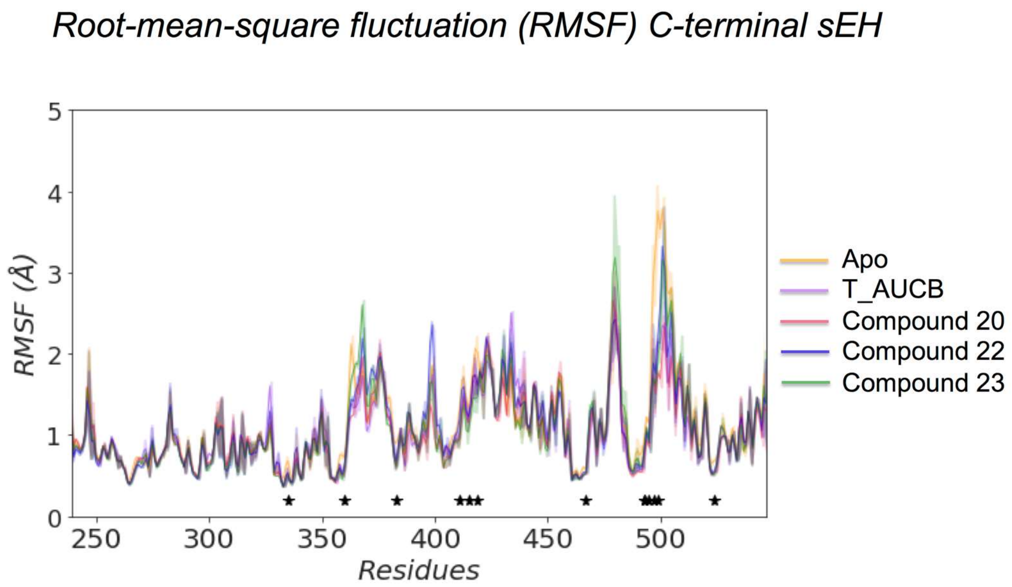

Figure S2: Root Mean Square Fluctuation (RMSF, in Å) of the C-terminal region of sEH either in the apo (orange line), *t*-AUCB bound (purple), compound **20** (red), **22** (blue), and **23** (green). Stars mark the sEH residues that occupy the active site region.

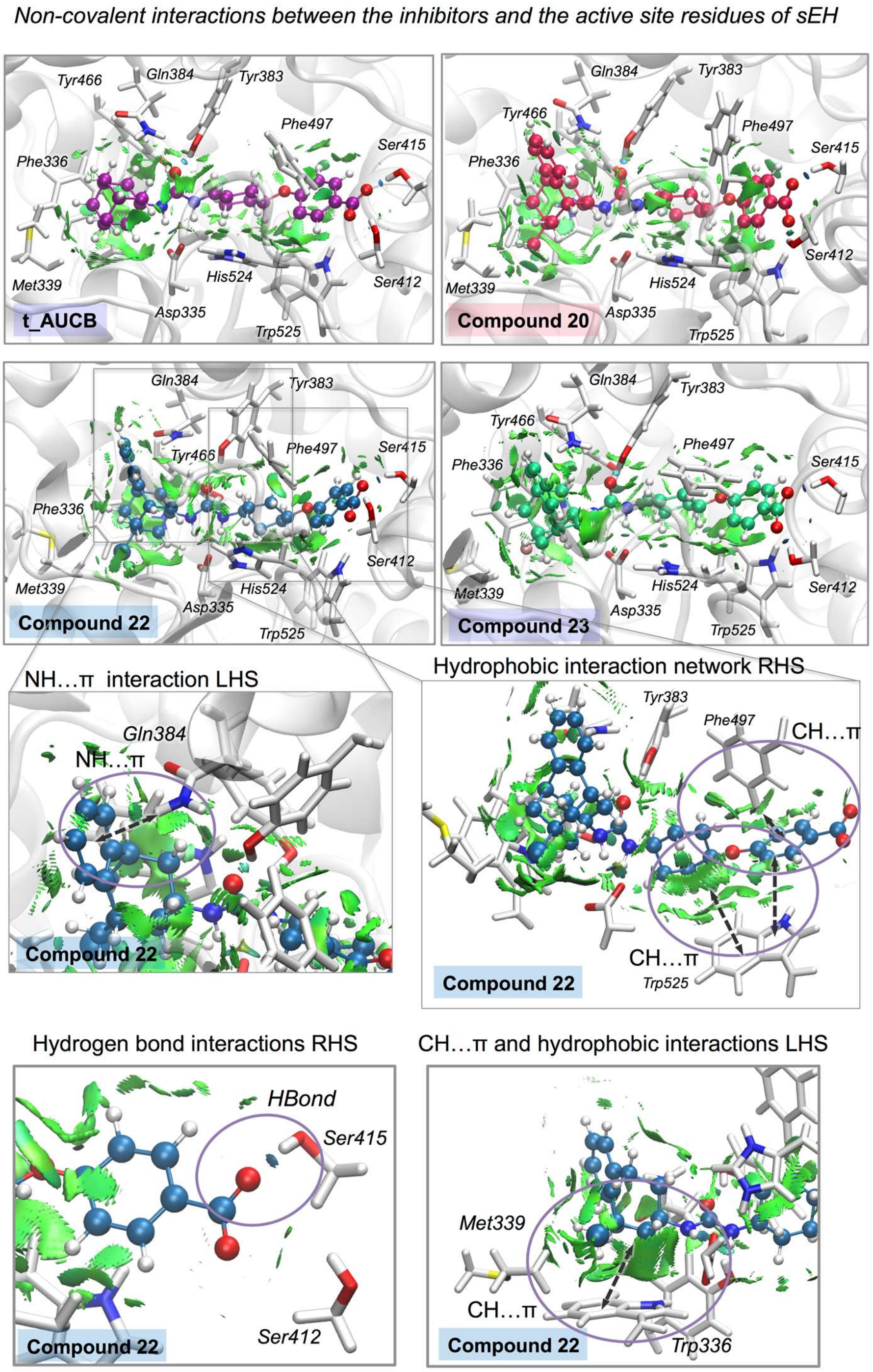

Figure S3: Representation of the noncovalent interactions (computed with NCIplot) at the active site of soluble epoxide hydrolase active site in the presence of compounds *t*-AUCB, **20**, **22**, and **23**. The weak interactions are shown as green surfaces, hydrogen-bonds are depicted in blue, and repulsive interactions in red. The interaction surface of the NH…π interation between the side chain of Gln384 and the aromatic ring of the benzohomoadamantane scaffold of compound **22** is highlighted. The network of hydrogen bonds, hydrophobic and CH…π interactions in both LHS and RHS pockets in the presence of compound **22** are depicted. The non-covalent interactions are calculated for the representative structures of the most populated cluster obtained from the MD simulations.

Analysis of active site water molecules in MD simulations

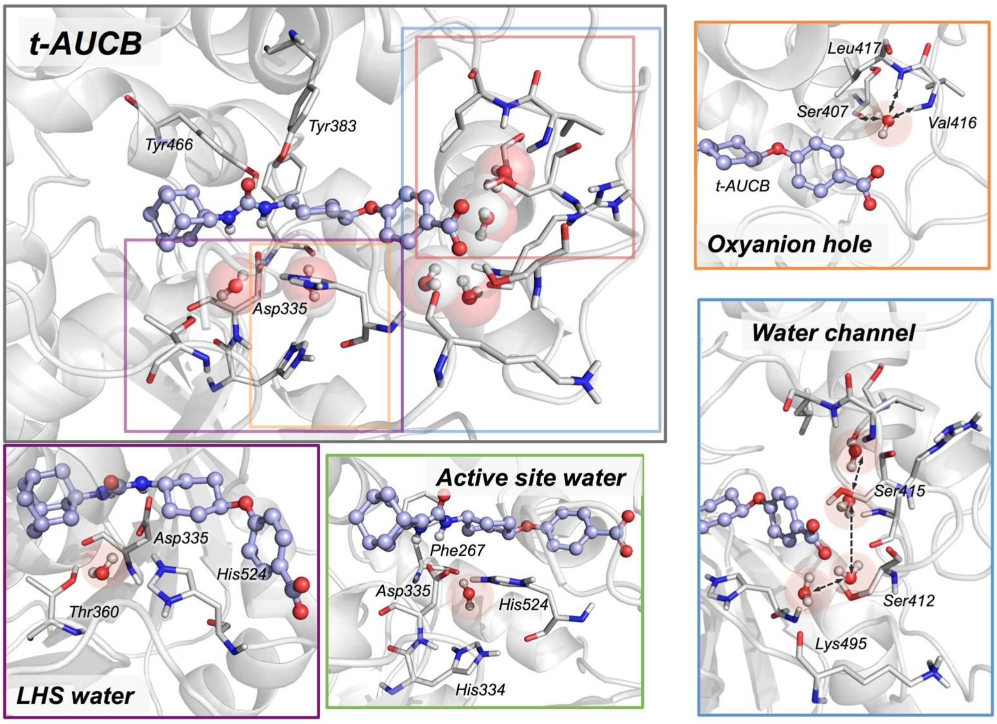

Figure S4: Representative MD snapshots displaying the water occupation in the RHS pocket in the presence of *t*-AUCB, oxyanion hole region, as well as the network of water channels are additionally provided. Representative MD snapshots showing the positioning of two water molecules: one occupying the RHS of the catalytic Asp335 (green panel), while the other one is positioned on the LHS (purple panel).

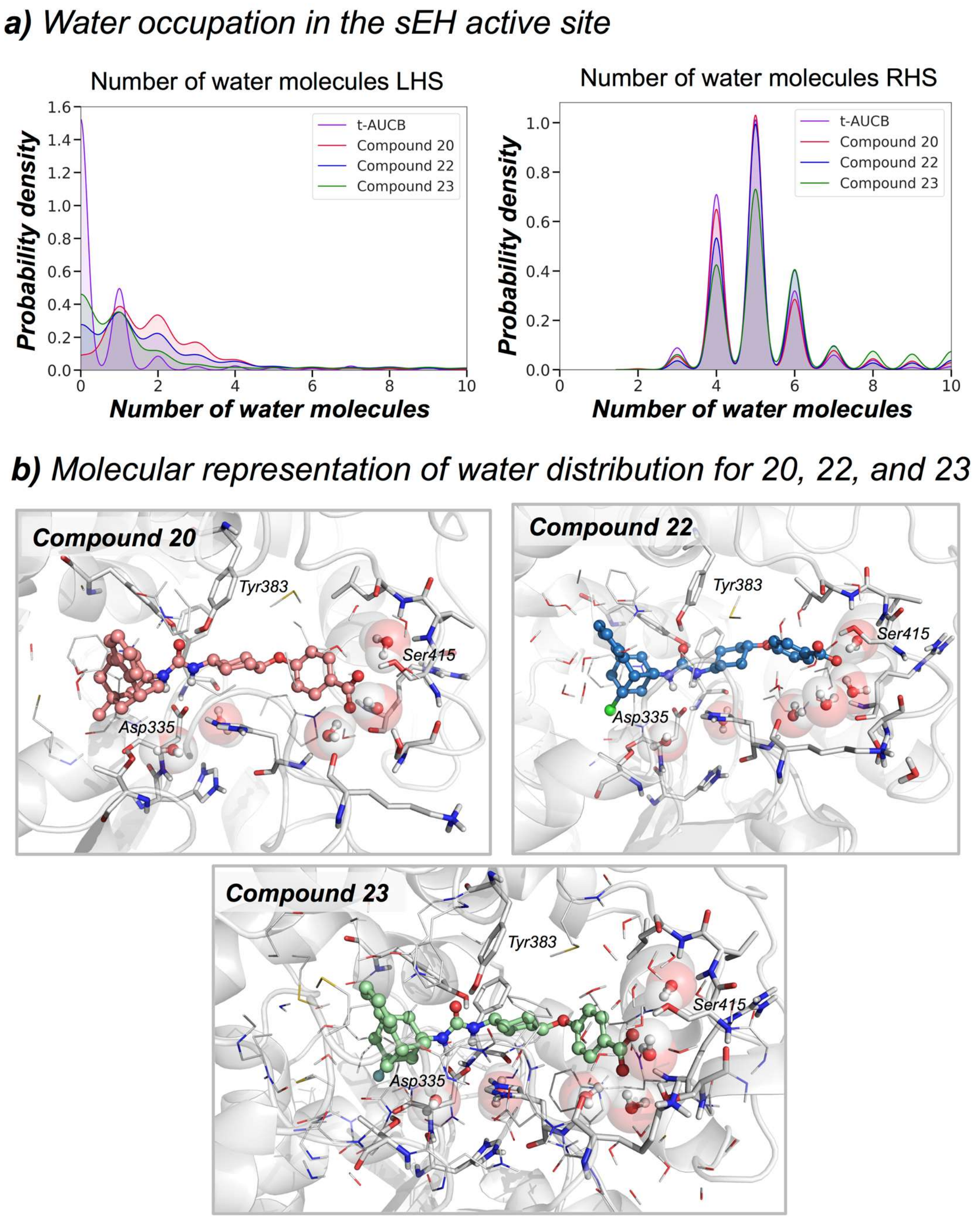

Figure S5: Representation of the normalized kernel density plot of the water distribution in the active site LHS and RHS pocket in the presence of *t*-AUCB (purple), **20** (red), **22** (blue), and **23** (green). We monitored the presence of water molecules through visual inspection of MD trajectories and using the *watershell* function of cpptraj MD analysis program.[2] Using *watershell*, we calculated the number of water molecules in the first solvation shell (using a distance cutoff of 3.4 Å) for both RHS (carboxylate group) and LHS (adamantane) pockets along the MD simulations. The average number of water molecules in the RHS pocket is 5.0 ± 1.4 (*t*-AUCB), 5.3 ± 1.8 (compound **20**), 5.6 ± 2.1 (compound **22**), and 6.1 ± 2.5 (compound **23**), respectively. The average number of water molecules in the LHS pocket is 1.0 ± 2.6 (*t*-AUCB), 2.8 ± 3.6 (compound **20**), 2.7 ± 3.9 (compound **22**), and 2.5 ± 4.5 (compound **23**).

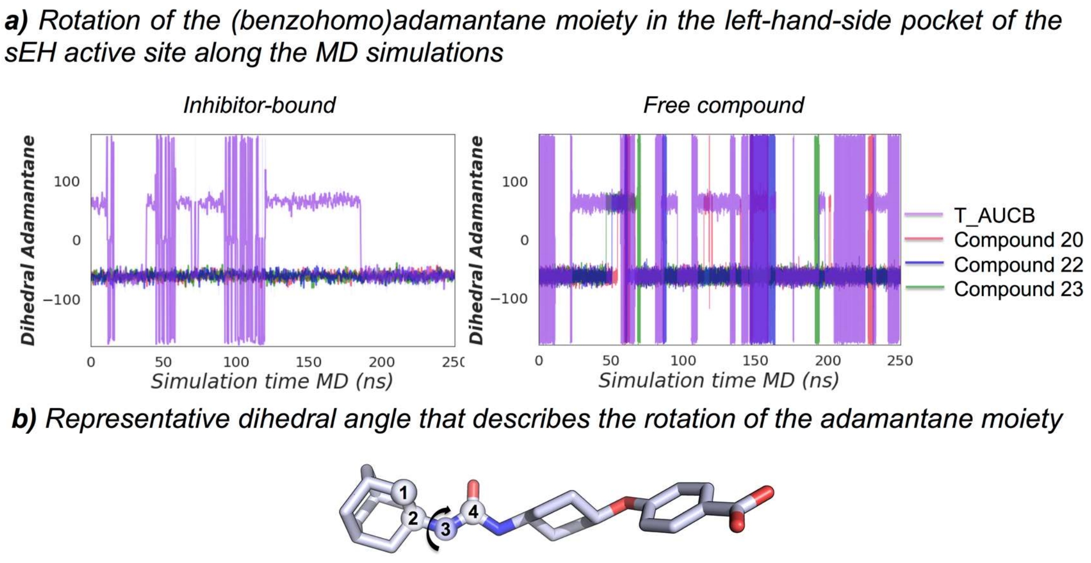

Figure S6: a) Plot of the dihedral angle that describes the rotation of the benzohomoadamantane moiety in the left-hand-side pocket of the sEH active site along the MD simulations (left) and when the compounds are free in the solvent (right). *t*-AUCB is shown in purple, compound **20** in red, compound **22** in blue, and compound **23** in green. b) Molecular representation of the dihedral angle used to monitor the rotation of the (benzohomo)adamantane scaffold.

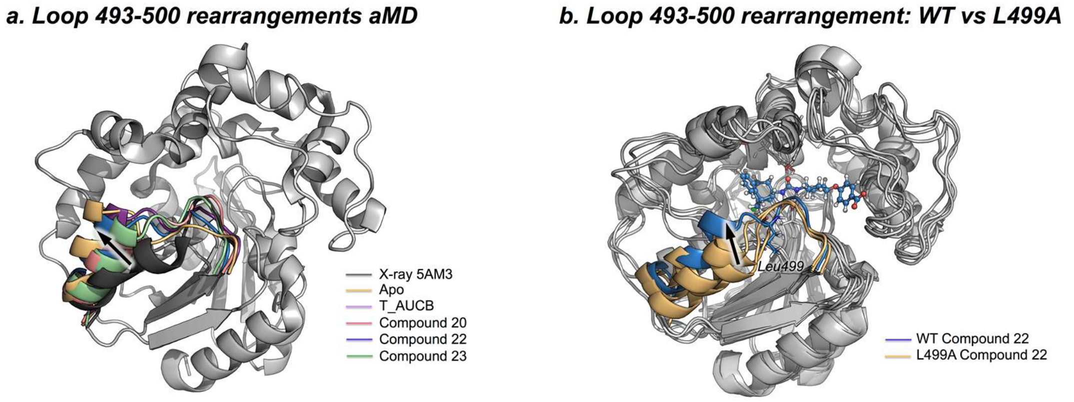

Figure S7: a) Overlay of the X-ray (in black), and representative snapshot from the apo state aMD simulations (yellow structure), and inhibitor-bound: *t*-AUCB (purple), and compounds **20** (red), **22** (blue), and **23** (green) simulations. The aMD parameters are calculated as described previously.[3] For *t*-AUCB, the boost potential applied to all dihedrals of the system is obtained from an energy threshold of 8101.08 kcal/mol and an alpha parameter value of 382.90 while a boost potential corresponding to an energy threshold of -217851.72 kcal/mol and alpha parameter of 12217.28 were applied to all atoms of the system. For **20**, the boost potential applied to all dihedrals of the system is obtained from an energy threshold of 8106.07 kcal/mol and an alpha parameter value of 382.90 while a boost potential corresponding to an energy threshold of -217635.12 kcal/mol and alpha parameter of 12218.88 were applied to all atoms of the system. For **22**, the boost potential applied to all dihedrals of the system is obtained from an energy threshold of 8101.1 kcal/mol and an alpha parameter value of 382.90 while a boost potential corresponding to an energy threshold of -217682.60 kcal/mol and alpha parameter of 12218.40 were applied to all atoms of the system. For **23**, the boost potential applied to all dihedrals of the system is obtained from an energy threshold of 8114.27 kcal/mol and an alpha parameter value of 382.90 while a boost potential corresponding to an energy threshold of -217735.60 kcal/mol and alpha parameter of 12218.40 were applied to all atoms of the system. B) Overlay of representative conventional MD snapshots for WT sEH (in blue), and three independent replicas of L499A variant (in yellow) showing the displacement of the loop 493-400 region.

Time	ID	Total Concentration (ng/mL)	Mean (ng/mL)	SD (ng/mL)
0 h	Mouse 1	0	0	0
	Mouse2	0		
	Mouse3	0		
0.25 h	Mouse 1	787	767.5	27.6
	Mouse2	5400[a]		
	Mouse3	748		
0.5 h	Mouse 1	1470	1610.0	475.7
	Mouse2	2140		
	Mouse3	1220		
1 h	Mouse 1	1000	970.3	345.5
	Mouse2	1300		
	Mouse3	611		
2 h	Mouse 1	475	397.3	112.8
	Mouse2	449		
	Mouse3	268		
4 h	Mouse 1	0	51.9	45.4
	Mouse2	71.3		
	Mouse3	84.3		
6 h	Mouse 1	21.7	37.3	14.3
	Mouse2	40.2		
	Mouse3	49.9		
24 h	Mouse 1	0	0	0
	Mouse2	0		
	Mouse3	0		

Table S2: Mean of concentrations of **20** in mouse plasma at different times after ip administration at 3 mg/Kg. [a]This mouse was excluded for mean and SD calculations because it was considered as a significant outlier $P<0.05$.

Time	ID	Total Concentration (ng/mL)	Mean (ng/mL)	SD (ng/mL)
0 h	Mouse 1	0	0	0
	Mouse2	0		
	Mouse3	0		
0.25 h	Mouse 1	151	354.7	241.9
	Mouse2	291		
	Mouse3	622		
0.5 h	Mouse 1	1350	1616.7	325.9
	Mouse2	1520		
	Mouse3	1980		
1 h	Mouse 1	600	1446.7	1063.3
	Mouse2	1100		
	Mouse3	2640		
2 h	Mouse 1	2440.0	3583.3	1089.9
	Mouse2	2640.0		
	Mouse3	5670.0		
4 h	Mouse 1	1240.0	1516.7	387.9
	Mouse2	1960.0		
	Mouse3	1350.0		
6 h	Mouse 1	1160	1141.3	398.3
	Mouse2	734.0		
	Mouse3	1530		
24 h	Mouse 1	181.0	104.6	71.0
	Mouse2	40.6		
	Mouse3	92.3		

Table S3: Mean of concentrations of **22** in mouse plasma at different times after ip administration at 3 mg/kg.

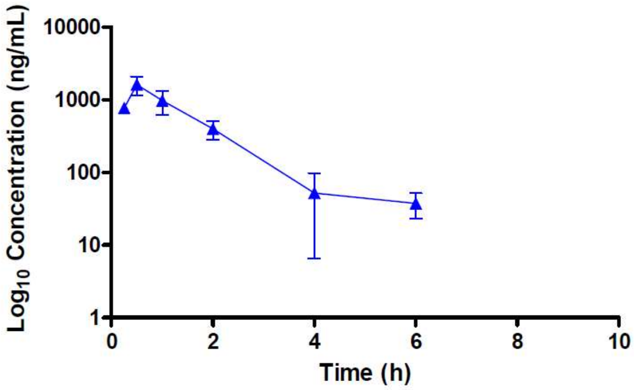

Figure S8: Plasma concentration (scale log 10) *vs* time for ip administration at 3 mg/kg of compound **20**.

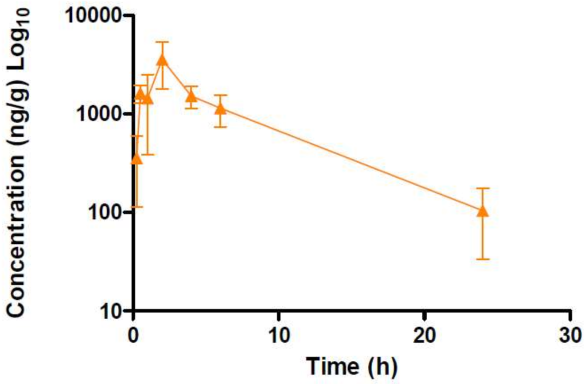

Figure S9: Plasma concentration (scale log 10) *vs* time for ip administration at 3 mg/kg of compound **22**.

Control Group	Sample 1	Sample 2	Sample 3	Sample 4	Sample 5
Parenchymal atrophy	0	0	0	0	0
Vacuolar degeneration of cells	0	0	0	0	0
Edema	0	0	0	0	0
Hemorrhage	0	0	0	0	0
Mononuclear inflammatory cells	0	0	0	0	0
Polimorfonuclear inflammatory cells	0	0	0	0	0
Necrosis	0	0	0	0	0
Total	0	0	0	0	0

Table S4: Histologic scoring of pancreatic tissues of control group.

Cerulein Group	Sample 1	Sample 2	Sample 3	Sample 4	Sample 5
Parenchymal atrophy	2	2	3	1	2
Vacuolar degeneration of cells	1	2	2	1	2
Edema	2	1	2	2	2
Hemorrhage	0	0	0	0	0
Mononuclear inflammatory cells	1	1	2	1	1
Polimorfonuclear inflammatory cells	1	2	3	1	2
Necrosis	1	0	2	1	0
Total	8	8	14	7	9

Table S5: Histologic scoring of pancreatic tissues of mice treated with cerulein.

Cerulein + 22 (0.1 mg/Kg)	Sample 1	Sample 2	Sample 3	Sample 4	Sample 5
Parenchymal atrophy	3	1	0	1	3
Vacuolar degeneration of cells	1	0	0	0	2
Edema	2	3	0	1	2
Hemorrhage	0	0	0	0	0
Mononuclear inflammatory cells	1	0	0	0	0
Polimorfonuclear inflammatory cells	1	2	0	1	3
Necrosis	2	1	0	0	0
Total	10	7	0	3	11

Table S6: Histologic scoring of pancreatic tissues of mice treated with cerulein and **22** at 0.1 mg/kg.

Cerulein + 22 (0.3 mg/Kg)	Sample 1	Sample 2	Sample 3	Sample 4	Sample 5
Parenchymal atrophy	1	0	1	1	2
Vacuolar degeneration of cells	0	0	1	0	1
Edema	1	1	1	1	0
Hemorrhage	0	0	0	0	0
Mononuclear inflammatory cells	0	0	0	0	0
Polimorfonuclear inflammatory cells	1	0	1	1	1
Necrosis	0	0	0	0	0
Total	3	1	4	3	4

Table S7: Histologic scoring of pancreatic tissues of mice treated with cerulein and **22** at 0.3 mg/kg.

REFERENCES

1- Durrant, J. D.; Votapka, L.; Sorensen, J.; Amaro, R. E. POVME 2.0: An enhanced tool for determining pocket shape and volume characteristics. *J. Chem. Theory Comput.* **2014**, *10*, 5047–5056.

2- Roe, D. R.; Cheatham, T. E., III. PTRAJ and CPPTRAJ: Software for processing and analysis of molecular dynamics trajectory data. *J. Chem. Theory Comput.* **2013**, *9*, 3084–3095.

3- Hamelberg, D.; de Oliveira, C. A. F.; McCammon, J. A. Sampling of slow diffusive conformational transitions with accelerated molecular dynamics. *J. Chem. Phys.* **2007**, *127*, 155102.
